# Benzo[*b*]naphtho[2,1-*d*]furans and 2-Phenylnaphthalenes from *Streblus usambarensis*

**DOI:** 10.1021/acs.jnatprod.3c00051

**Published:** 2023-04-12

**Authors:** Carolyne Chepkirui, Fozia Ali Adem, Anastasia Rudenko, Yukino Gütlin, Albert Ndakala, Solomon Derese, Andreas Orthaber, Catarina Bourgard, Abiy Yenesew, Máté Erdélyi

**Affiliations:** † Department of Chemistry, University of Nairobi, P.O. Box 30197, 00100 Nairobi, Kenya; ‡ Department of Physical and Biological Sciences, Kabarak University, Private Bag-20157, Nakuru, Kenya; ⊥ Department of Chemistry and Molecular Biology, University of Gothenburg, and Centre for Antibiotic Resistance Research (CARe) at the University of Gothenburg, SE-405 30 Gothenburg, Sweden; § Department of Chemistry - Ångström, Uppsala University, SE-751 20 Uppsala, Sweden; ∥ Department of Chemistry - BMC, Uppsala University, SE-752 37 Uppsala, Sweden

## Abstract

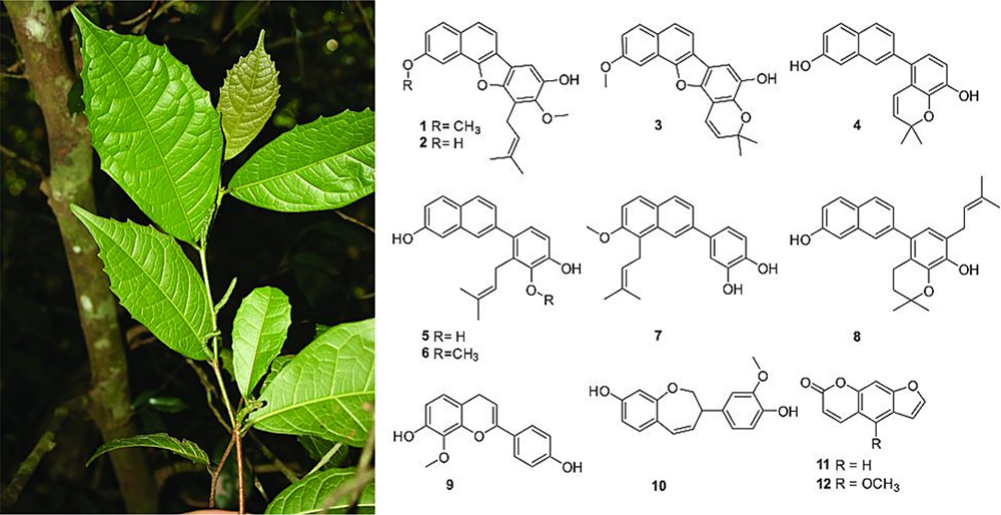

Three new benzo[*b*]naphtho[2,1-*d*]furans, usambarins A–C (**1**–**3**), five new 2-phenylnaphthalenes, usambarins D–H (**4**–**8**), a new flavan (**9**), and a new phenyl-1-benzoxepin (**10**) as well as two known compounds (**11** and **12**) were isolated from the extract of the stem and roots of *Streblus usambarensis* (Moraceae). The structures were deduced using NMR spectroscopic and mass spectrometric analyses, and those of compounds **1** and **4** were confirmed by X-ray crystallography. Usambarin D (**4**) demonstrated moderate antibacterial activity (MIC 9.0 μM) against *Bacillus subtilis*, while none of the tested compounds were effective against *Escherichia coli*.

*Streblus* (Moraceae) is a genus of small deciduous shrubs with approximately 25 species found in tropical India, Malaysia, South Africa, Thailand, and the Philippines. Some of these species are in ethnomedical use in the treatment of leprosy,^1^ dysentery,^2^ filariasis,^3^ toothache,^4^ inflammation,^5^ and cancer,^6^ and some were reported to contain cardiac glycosides, coumarins, flavonoids, and lignans^6−9^ that have anticancer,^6,7,9,10^ antiparasitic,^6,11^ and antibacterial properties.^6,7,12−14^*Streblus usambarensis* is an evergreen shrub with a smooth brown bark that has not yet been phytochemically studied. It is found in the coastal region of Kenya, Tanzania, Guinea, south of Nigeria, and Mozambique.^15^ We report herein the isolation of 10 new (**1**–**10**) and two known compounds (**11** and **12**) from its stem and roots and the evaluation of their antibacterial and cytotoxic activities.
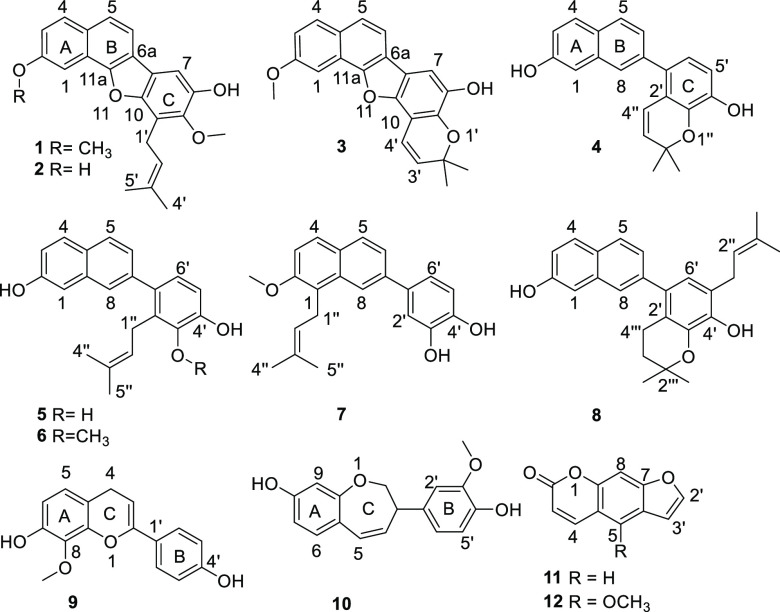


Chromatographic separation of the CH_2_Cl_2_/MeOH (1:1) extracts of the roots of *S. usambarensis* on silica gel followed by purification on Sephadex LH-20, preparative TLC, and preparative HPLC led to the isolation of three new compounds (**1**–**3**) and bergaptol (**11**).^16^ Investigation of its stem extract provided seven new compounds (**4**–**10**) and bergapin (**12**)^17^ (Figure 1).

**Figure 1:**
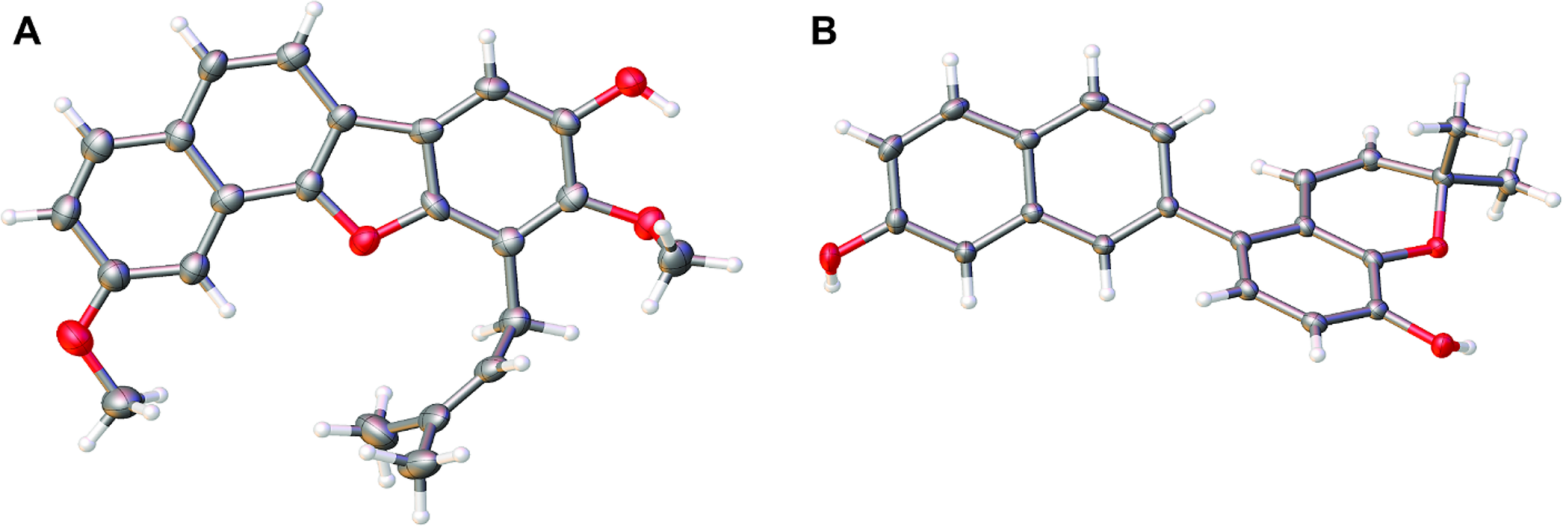
Solid-state structures of (A) usambarins A (**1**) and (B) D (**4**); thermal ellipsoid plots at 50% probability levels.

Compound **1** was obtained as a white solid and was given the molecular formula C_23_H_22_O_4_ based on HRESIMS analysis (*m*/*z* 363.1596 [M + H]^+^, calcd 363.1591, Figure S8, Supporting Information). Its NMR data (Table 1 and Figures S1–S7, Supporting Information) suggested a benzo[*b*]naphtho[2,1-*d*]furan skeleton, substituted with hydroxy, a γ,γ-dimethylallyl, and two methoxy substituents. Its ring A showed an AMX spin system with protons at δ_H_ 8.00 (d, *J* = 8.9 Hz, H-4), 7.59 (d, *J* = 2.6 Hz, H-1), and 7.23 (dd, *J* = 8.9 Hz, 2.6 Hz, H-3), with the corresponding carbons resonating at δ_C_ 98.7 (C-1), 117.9 (C-3), and 130.3 (C-4). One of the OMe groups (δ_H_ 3.97) was placed at C-2 of this ring based on its HMBC correlation with δ_C_ 157.9 (C-2) (Table 1, Figure S5, Supporting Information) and by its NOESY correlation with H-1 (δ_H_ 7.59) and H-3 (δ_H_ 7.23) (Figure S6, Supporting Information). Its ring B contains *ortho*-coupled protons (*J* = 8.4 Hz) at δ_H_ 7.78 (H-5) and δ_H_ 7.89 (H-6) with the corresponding carbons resonating at δ_C_ 122.8 (C-5) and δ_C_ 116.1 (C-6). Its ring C is trisubstituted with a hydroxy, a methoxy, and a γ,γ-dimethylallyl group with a single aromatic proton appearing as a singlet at δ_H_ 7.38 (H-7, δ_C_ 103.9). The hydroxy group (δ_H_ 9.35) was placed at C-8 (δ_C_ 147.3) based on its HMBC correlation (Table 1, Figure S5, Supporting Information) with C-7 (δ_C_ 103.9) and C-8 (δ_C_ 147.3) and the γ,γ-dimethylallyl group at C-10 (δ_C_ 118.9) based on the correlation of δ_H_ 3.71 (H_2_-1′) with C-9 (δ_C_ 145.5) and C-10 (δ_C_ 118.9). This unprecedented skeleton was confirmed by X-ray crystallography (Figure 1). The compound crystallizes in the monoclinic space group *P*21/*c* with one molecule in the asymmetric unit. The aromatic core is nearly coplanar (average deviation from the least-squares plane = 0.026 Å). Short O–H···H distances (2.218(5) Å) of equivalent C8-OH moieties give rise to a 1-D herringbone-like hydrogen-bonded network. Based on the above spectroscopic and crystallographic data, this new compound, usambarin A (**1**), was characterized as 2,9-dimethoxy-10-(3-methylbut-2-en-1-yl)naphtho[1,2-*b*]benzofuran-8-ol.

**Table 1:** NMR Spectroscopic Data (600 MHz, DMSO-*d*_6_) for Usambarin A (**1**)

position	δ_C_, type	δ_H_ (*J* in Hz)	HMBC^a^
1	98.7 CH	7.59 d (2.6)	2, 3, 4a, 11a
2	157.9 C		
3	117.9 CH	7.23 dd (8.9, 2.6)	1,2, 4a
4	130.3 CH	8.00 d (8.9)	2, 4a, 5, 11b
4a	127.3 C		
5	122.8 CH	7.78 d (8.4)	4, 4a, 6a, 6, 11a, 11b
6	116.1 CH	7.89 d (8.4)	4a, 5, 6b, 11a
6a	119.8 C		
6b	119.3 C		
7	103.9 C	7.38 s	6b, 8, 9
8	147.3 C		
9	145.5 C		
10	118.9 C		
10a	147.8 C		
11a	150.7 C		
11b	121.6 C		
1′	23.3 CH_2_	3.71d (7.5)	2′, 3′, 9, 10, 10a
2′	122.1 CH	5.35 m	1′, 4′, 5′
3′	131.5 C		
4′	17.7 CH_3_	1.97 m	2′, 5′
5′	24.9 CH_3_	1.69 d (1.6)	2′, 4′
2-OMe	55.3 CH_3_	3.97 s	2
8-OH		9.35 s	7, 8, 9
9-OMe	60.5 CH_3_	3.83 s	9

a HMBC correlations, optimized for 6 Hz, are from stated proton(s) to the indicated carbon.

Compound **2** was isolated as a white solid. Its HRESIMS spectrum (Figure S19, Supporting Information) exhibited a protonated molecular ion [M + H]^+^ at *m*/*z* 349.1440 (calcd 349.1434) corresponding to the molecular formula C_23_H_22_O_4_. Its ^1^H and ^13^C NMR data (Table 2, Figure S12–S18, Supporting Information) together with its 2D spectra showed similar spectroscopic features to those of **1**, except for its C-2 substituent being a hydroxy instead of a methoxy group in **1**. This was indicated by the similar chemical shift of C-2 in **1** (δ_C_ 157.9) and **2** (δ_C_ 157.3) and by the 2-OMe signal (δ_H_ 3.97, δ_C_ 55.3) of **2** missing in the spectra of **1**. Based on the above spectroscopic evidence, this new compound, usambarin B (**2**), was characterized as 9-methoxy-10-(3-methylbut-2-en-1-yl)naphtho[1,2-*b*]benzofuran-2,8-diol.

**Table 2:** NMR Spectroscopic Data (500 MHz, CD_3_OD) for Usambarin B (**2**)

position	δ_C_, type	δ_H_*m* (*J* in Hz)	HMBC^a^
1	103.2 CH	7.59 d (2.4)	3
2	157.3 C		
3	118.8 CH	7.10 dd (8.8, 2.5)	1, 4a
4a	128.8 C		
4	131.3 CH	7.84 d (8.8)	2, 5
5	123.9 CH	7.63 d (8.5)	4, 4a, 6a, 6b
6a	124.0 C		
6b	121.1 C		
6	116.0 CH	7.68 d (8.4)	4a, 11a, 11b
7	104.4 CH	7.29 s	6b, 8, 9
8	148.3 C		
9	146.7 C		
10a	150.0 C		
10	120.6 C		
11a	152.7 C		
11b	121.6 C		
1′	24.6 CH_2_	3.75 d (7.4)	2′, 3′, 9, 10, 10a
2′	123.4 CH	5.44 m	
3′	133.1 C		
4′	26.0 CH_3_	1.73	2′, 4, 5′
5′	18.2 CH_3_	1.99 d (1.4)	2′, 3′, 4′
9-OMe	61.6 CH_3_	3.89 s	9

a HMBC correlations, optimized for 6 Hz, are from stated proton(s) to the indicated carbon.

Compound **3** was isolated as a white solid from the roots of *S. usambarensis*. HRESIMS (Figure S28, Supporting Information) indicated a protonated molecular ion [M + H]^+^ peak at *m*/*z* 347.1283 (calcd 347.1278), which along with the NMR data (Table 3, Figures S21–S27, Supporting Information) established the molecular formula C_23_H_22_O_4_. Its UV (λ_max_ 270, 350 nm) and NMR data were similar to those of compounds **1** and **2**, suggesting it to also have a naphtho[1,2-*b*]benzofuran skeleton. Rings A and B of **3** showed highly similar spectroscopic features to compounds **1** and **2** and are thus similarly substituted, whereas its ring C holds a pyran ring instead of the methoxy and the γ,γ-dimethylallyl groups attached at C-9 and C-10 of **1** and **2**, respectively. The only aromatic proton (δ_H_ 7.36, δ_C_ 104.6) of ring C of **3** was assigned to H-7, based on its HMBC correlations to C-6b (δ_C_ 120.5), C-8 (δ_C_ 138.7), C-9 (δ_C_ 141.8), C-10 (δ_C_ 106.8, weak), and C-10a (δ_C_ 146.1). The HMBC spectrum (Table 3, Figure S25, Supporting Information) further supported the placement of a hydroxy group at C-8 (δ_C_ 138.7), as the corresponding signal at δ_H_ 5.45 (8-OH) shows cross-peaks to C-8 (δ_C_ 138.7) and C-9 (δ_C_ 141.8). The HMBC correlation of δ_H_ 7.06 (H-4′) to C-9 (δ_C_ 141.7) and C-10 (δ_C_ 106.8) indicated the location of the pyran ring at C-9/C-10. Based on the above spectroscopic evidence, this new compound, usambarin C (**3**), was characterized as 11-methoxy-3,3-dimethyl-3*H*-naphtho[2′,1′:4,5]furo[2,3-*f*]chromen-5-ol.

**Table 3:** NMR Spectroscopic Data (500 MHz, CDCl_3_) for Usambarin C (**3**)

position	δ_C_, type	δ_H_ (*J* in Hz)	HMBC^a^
1	99.1 CH	7.67 s	2, 4, 11a, 11b
2	158.3 C		
3	118.2 CH	7.16 dd (9.0, 2.6)	1, 2
4	130.2 CH	7.85 d (8.9)	1, 2, 3, 5
4a	117.8 C		
5	123.0 CH	7.64 d (8.3)	4, 6a, 6b
6a	127.8 C		
6b	120.5 C		
6	115.8 CH	7.73 d (8.3)	6a, 6b, 11a, 11b
7	104.6 CH	7.36 s	6b, 8, 9, 10, 10a
8	138.7 C		
9	141.8 C		
10 a	146.1 C		
10	106.8 C		
11a	151.6 C		
11b	122.4 C		
2′	78.0 C		
3′	130.8 CH	5.81 d (9.9)	2′, 4, 4′, 8, 2′-Me
4′	116.7 CH	7.06 d (9.8)	2′, 4, 8, 9, 10a, 2′-Me
2′-Me_2_	27.9 CH_3_	1.56 d (2.0)	3′, 4′
2-OMe	55.7 CH_3_	4.03 s	2
8-OH		5.45 s	7, 8, 9

a HMBC correlations, optimized for 6 Hz, are from stated proton(s) to the indicated carbon.

Compound **4** was isolated as a white solid from the stem of *S. usambarensis* and was given the molecular formula C_23_H_22_O_4_ based on the observation of the protonated molecular ion peak [M + H]^+^ at *m*/*z* 319.1334 (calcd 319.1329, Figure S36, Supporting Information). Its NMR data (Table 4, Figures S29–S35, Supporting Information) suggested it to have a 2-phenylnaphthalene skeleton. Ring A of thenaphthol moiety showed an AXY spin system with protons resonating at δ_H_ 7.78 (dd, *J* = 8.5, 3.4 Hz, H-4; δ_C_ 129.7 for C-4), 7.16 (d, *J* = 2.5 Hz, H-1 δ_C_ 109.7 for C-1), and 7.11 (dd, *J* = 8.5, 1.8 Hz, H-3; δ_C_ 117.9 for C-3) and a hydroxy group at C-2 (δ_C_ 153.9). Ring B also possesses three mutually coupled aromatic protons at δ_H_ 7.78 (dd, *J* = 8.5, 3.4 Hz, H-5), 7.59 (br s, H-8), and 7.30 (dd, *J* = 8.5, 1.8 Hz, H-6), with the corresponding carbon atoms resonating at δ_C_ 127.6 (C-5), 126.2 (C-6), and 126.8 (C-8), and it is linked to the phenol moiety, ring C, via its C-7 (δ_C_ 138.1). Ring C has *ortho*-coupled (*J* = 8.3 Hz) aromatic protons at δ_H_ 6.87 (H-5′) and δ_H_ 6.89 (H-6′) and is substituted with a hydroxy and a 2,2-dimethylpyrano group. The hydroxy group (δ_H_ 5.55) is located at C-4′ (δ_C_ 144.2), based on its HMBC correlations (Table 4, Figure S33, Supporting Information) to C-3′ (δ_C_ 139.6), C-4′ (δ_C_ 144.2), and C-5′ (δ_C_ 122.4). The pyran ring was placed at C-2′/C-3′ based on the HMBC correlations of H-4″ (δ_H_ 6.40) to C-2′ (δ_C_ 119.2) and C-3′ (δ_C_ 139.6). The structure of this compound, which possesses a new skeleton, was corroborated by single-crystal X-ray crystallographic analysis (Figure 1). The compound crystallizes in the centrosymmetric triclinic space group *P*1̅ with one molecule in the asymmetric unit. The two aromatic subunits are folded with respect to each other by 18.3(1)°. A linear hydrogen-bonded network (*d*(O···H) = 2.201(1) Å) involves both hydroxy groups. Based on the above spectroscopic and crystallographic data, this new compound, usambarin D (**4**), was characterized as 5-(7-hydroxynaphthalen-2-yl)-2,2-dimethyl-2*H*-chromen-8-ol.

**Table 4:** NMR Spectroscopic Data (500 MHz, CDCl_3_) for Usambarin D (**4**)

position	δ_C_, type	δ_H_ (*J* in Hz)	HMBC^a^
1	109.7 CH	7.16 d (2.5)	2, 3, 4, 8
2	153.9 C		
3	117.9 CH	7.11 dd (8.5, 1.8)	1, 4
4	129.7 CH	7.78 dd (8.5, 3.4)	1, 2, 4a, 5
4a	127.9 C		
5	127.6 CH	7.78 d (8.5, 3.4)	4, 4a, 5
6	126.2 CH	7.30 dd (8.5, 1.8)	1′, 6
7	138.1 C		
8a	134.6 C		
8	126.8 CH	7.59 s	1, 1′, 6, 8a,
1′	131.5 C		
2′	119.2 C		
3′	139.6 C		
4′	144.2 C		
5′	122.4 CH	6.90 d (8.3)	1′, 3′, 4′
6′	114.6 CH	6.87 d (8.3)	2′, 4′, 7
2″	76.5 C		
3″	130.5 CH	5.61 d (10.1)	2′, 2″, 2″-Me,
4″	121.1 CH	6.40 d (10.1)	1′, 2′, 3′, 2″, 3″
2″Me	27.9 CH_3_	1.52 s	2″, 2″Me, C-3″
2″Me2	27.9 CH_3_	1.52 s	
2-OH		5.13	2, 3, 4
4′-OH		5.55 s	3′, 4′, 5′

a HMBC correlations, optimized for 6 Hz, are from stated proton(s) to the indicated carbon.

Compound **5** was isolated as a white solid and was assigned the molecular formula C_21_H_20_O_3_ based on ESIMS analysis (*m*/*z* 319.2 [M – H]^−^, Figure S47, Supporting Information) and NMR data (Table 5, Figures S40–S46, Supporting Information). The NMR data further suggested the compound to have a 2-phenylnaphthalene skeleton. Its rings A and B showed highly similar NMR spectroscopic features to those of compound **4**, revealing them to be identical to those of **4**. The NMR data of its ring C showed an analogous substitution pattern to **4**, with *ortho*-coupled (*J* = 8.2 Hz) protons at δ_H_ 6.83 (H-5′) and 6.86 (H-6′); however, it is substituted with a γ,γ-dimethylallyl moiety and two hydroxy groups (4′-OH δ_H_ 5.43, and 3′-OH δ_H_ 5.55) instead of the 2,2-dimethylpyrano ring and only one hydroxy group of **4**. The γ,γ-dimethylallyl substituent was positioned at C-2′ (δ_C_ 125.4) based on the HMBC (Table 5, Figure S44, Supporting Information) cross-peaks of CH_2_-1″ (δ_H_ 3.36) with C-2′ (δ_C_ 125.4) and C-3′ (δ_C_ 142.4). The placement of the hydroxy groups at C-3′ (δ_C_ 142.4) and C-4′ (δ_C_ 144.0) was in agreement with the chemical shifts of the oxygenated carbons of this ring, confirmed by the HMBC correlations of 3′-OH (δ_H_ 5.55) to C-2′ (δ_C_ 125.4), C-3′ (δ_C_ 142.4), and C-4′ (δ_C_ 144.0) and of 4′-OH (δ_H_ 5.43) to C-3′ (δ_C_ 142.4), C-4′ (δ_C_ 144.0), and C-5′ (δ_C_ 122.8) and by the NOE correlation of CH_2_-2″ (δ_H_ 5.29) with 3′-OH (δ_H_ 5.55). Based on the above spectroscopic data, this new compound, usambarin E (**5**), was characterized as 4-(7-hydroxynaphthalen-2-yl)-3-(3-methylbut-2-en-1-yl)benzene-1,2-diol.

**Table 5:** NMR Spectroscopic Data (500 MHz, CDCl_3_) for Usambarin E (**5**)

position	δ_C_, type	δ_H_ (*J* in Hz)	HMBC^a^
1	109.7 CH	7.14 d (2.6)	2, 3, 4a, 5
2	153.8 C		
3	117.8 CH	7.10 dd (8.8, 2.6)	4
4	129.8 CH	7.76 dd (8.6, 3.0)	3, 8, 8a
4a	139.9 C		
5	127.5 CH	7.76 dd (8.6, 3.0)	1, 6, 8a
6	126.2 CH	7.24 d (1.8)	1′, 8
7	134.6 C		
8	126.8 CH	7.55 s	4, 4a, 6, 7
8a	127.8 C		
1′	134.9 C		
2′	125.4 C		
3′	142.4 C		
4′	144.0 C		
5′	122.8 CH	6.83 d (8.2)	1′, 3′
6′	112.9 CH	6.86 d (8.2)	2′, 4′
1″	27.7 CH_2_	3.36 d (6.8)	2′, 3′, 2″, 3″
2″	122.2 CH	5.29 m	4″, 5″
3″	135.7 C		
4″	25.9 CH_3_	1.68 s	2″, 3″
5″	18.1 CH_3_	1.76 s	2″, 3″
2-OH		5.00 s	1, 2, 3
3′-OH		5.55 s	2′, 3′, 4′
4′-OH		5.43 s	3′, 4′, 5′

a HMBC correlations, optimized for 6 Hz, are from stated proton(s) to the indicated carbon.

Compound **6** provided an HRESIMS analysis ([M + H]^+^*m*/*z* 335.1647, calcd 335.1642, Figure S56, Supporting Information) that suggested the molecular formula C_23_H_22_O_4_. Its ^1^H and ^13^C NMR data, along with its 2D spectra (Table 6, Figures S49–S55, Supporting Information), showed similar spectroscopic features to those observed for **5**, except for a methoxy substituent at C-3′ in its ring C instead of a hydroxy substituent in compound **5**. The ^13^C NMR chemical shift of this methoxy group (δ_C_ 61.4) is deshielded, which is diagnostic for di-*ortho*-substituted aromatic rings.^18^ The position of this methoxy substituent was further corroborated by the HMBC of 5′-OMe (δ_H_ 3.86) to C-3′ (δ_C_ 145.8) and the NOE of 5′-OMe (δ_H_ 3.86) to CH_2_-1″ (δ_H_ 3.33) and CH-2″ (δ_H_ 5.08). Based on the above spectroscopic evidence, this new compound, usambarin F (**6**), was characterized as 7-(4-hydroxy-3-methoxy-2-(3-methylbut-2-en-1-yl)phenyl)naphthalen-2-ol.

**Table 6:** NMR Spectroscopic Data (500 MHz, CDCl_3_) for Usambarin F (**6**)

position	δ_C_, type	δ_H_ (*J* in Hz)	HMBC^a^
1	109.7 CH	7.12 d (2.5)	2, 3, 8a
2	153.8 C		
3	117.7 CH	7.10 dd (8.7, 2.6)	1, 2
4	129.7 CH	7.78 dd (8.5, 2.6)	2, 5, 4a
4a	134.6 C		
5	127.4 CH	7.78 d (8.5, 2.6)	1, 6
6	126.2 CH	7.25 m	1′, 5
7	139.9 C		
8a	127.8 C		
8	126.9 CH	7.55 m	1, 1′, 6, 8a
1′	135.7 C		
2′	133.3 C		
3′	145.6 C		
4′	148.5 C		
5′	113.3 CH	6.90 d (8.2)	1′, 3′, 4′,
6′	126.9 C	6.97 d (8.3)	2′, 4′, 7
1″	26.9 CH_2_	3.33 dt (6.6,1.3)	1′, 2′, 3′, 2″, 3″
2″	123.5 CH	5.08 m	4″, 5″
3″	131.5 C		
4″	25.8 CH_3_	1.58 d (1.4)	2″, 3″, 5″
5″	17.8 CH_3_	1.35 d (1.4)	2″, 3″, 4″
2-OH		5.58 s	
4′-OH		5.30 s	
3′-OMe	61.4	3.86 s	5′

a HMBC correlations, optimized for 6 Hz, are from stated proton(s) to the indicated carbon.

Compound **7** was obtained as a white solid and presented the molecular formula C_22_H_22_O_3_ from HRESIMS analysis (*m*/*z* 335.1647 [M + H]^+^, calcd 335.1642, Figure S65, Supporting Information). Its NMR data (Table 7, Figures S58–S64, Supporting Information) suggested a 2-phenylnaphthalene skeleton, similar to compounds **4**–**6**. In ring A, the ^1^H NMR data (Table 7, Figure S59, Supporting Information) revealed *ortho*-coupled (*J* = 8.9 Hz) protons resonating at δ_H_ 7.26 (H-3) and δ_H_ 7.72 (H-4) with the corresponding carbons at δ_C_ 113.8 (C-3) and 127.4 (C-4). This ring is disubstituted with a methoxy and a γ,γ-dimethylallyl group, the former being positioned at C-2 (δ_C_ 154.7) based on its (δ_H_ 3.95) HMBC correlation (Table 7, Figure S63, Supporting Information) to C-2 (δ_C_ 154.7). The HMBC experiments further indicated the placement of the γ,γ-dimethylallyl substituent at C-1 (δ_C_ 128.5) through the correlation of CH_2_-1″ (δ_H_ 3.83) with C-1 (δ_C_ 128.5), C-2 (δ_C_ 154.7), and C-8a (δ_C_ 133.4). Ring B of compound **7** is similar to those of compounds **4**–**6**, as indicated by its ^1^H and ^13^C NMR data along with its 2D spectra. The aromatic protons of its ring C possess an AMX spin system with protons resonating at δ_H_ 7.25 (d, *J* = 2.2 Hz, H-2′), 7.17 (dd, *J* = 8.2, 2.1, Hz, H-6′), and 6.98 (d, *J* = 8.2 Hz, H-5′) and with the corresponding carbon atoms resonating at δ_C_ 114.8 (C-2′), 115.9 (C-6′), and 120.4 (C-5′), respectively, consistent with disubstitution. These two hydroxy groups were placed at C-3′ (δ_C_ 143.2) and C-4′ (δ_C_ 143.9) based on the HMBC correlations of 3′-OH (δ_H_ 5.25) to C-4′ (δ_C_ 143.9) and C-2′ (δ_C_ 114.8) and of 4′-OH (δ_H_ 5.21) to C-3′ (δ_C_ 143.2) and C-5′ (δ_C_ 115.9) and the NOEs of 3′-OH (δ_H_ 5.25) to H-2′ (δ_H_ 7.25), CH_2_-1″ (δ_H_ 3.83), and H-8 (δ_H_ 8.02) and of 4′-OH (δ_H_ 5.21) to H-5′ (δ_H_ 6.98). Based on the above spectroscopic evidence, this new compound, usambarin G (**7**), was characterized as 4-(7-methoxy-8-(3-methylbut-2-en-1-yl)naphthalen-2-yl)benzene-1,2-diol.

**Table 7:** NMR Spectroscopic Data (500 MHz, CDCl_3_) for Usambarin G (**7**)

position	δ_C_, type	δ_H_ (*J* in Hz)	HMBC^a^
1	128.5 C		
2	154.7 C		
3	113.8 CH	7.25 m	1, 2, 2″
4	127.4 CH	7.72 d (8.9)	2, 4a, 5
5	129.1 CH	7.81 d (8.5)	1′, 4, 4a
5a	133.4 C		
6	123.0 CH	7.52 dd (8.5, 1.8)	5, 6, 8
7	138.3 C		
8a	133.4 C		
8	121.4 CH	8.04 s	1, 6, 7
1′	135.5 C		
2′	114.8 CH	7.25 m	1′, 5′, 6′
3′	143.2 C		
4′	143.9 C		
5′	115.9 CH	6.98 d (8.2)	4′
6′	120.4 CH	7.17 dd, (8.2, 2.1)	1′, 3′, 2′
1″	24.3 CH_2_	3.83 d (6.8)	2, 2″, 3″
2″	123.5 CH	5.23 m	4″, 5″
3″	131.6 C		
4″	18.3 CH_3_	1.91 d (1.3)	2″, 3″
5″	25.9 CH_3_	1.69 d (1.3)	2″, 3″
2-OMe	57.0	3.95 s	
3′-OH		5.25 m	2′, 4′
4′-OH		5.21 m	3′, 5′

a HMBC correlations, optimized for 6 Hz, are from stated proton(s) to the indicated carbon.

Compound **8** was isolated as a white solid with the molecular formula C_23_H_22_O_4_ based on HRESIMS analysis (*m*/*z* 389.2117 [M + H]^+^, calcd 389.2111, Figure S74, Supporting Information). Its ^1^H and ^13^C NMR data (Table 8, Figures S67–S73, Supporting Information) together with its 2D spectra showed similar spectroscopic features to those of compounds **4**–**6**, indicating an analogous 2-phenylnaphthalene skeleton and identical substitution on its rings A and B. Its ring C is different from those of **4**–**6**, and thus has one aromatic proton appearing as a singlet at δ_H_ 6.75 (H-6′) and a 2,2-dimethyldihydropyrano, a γ,γ-dimethylallyl, and a hydroxy substituent. Its C-3′ (δ_C_ 139.9) and C-4′ (δ_C_ 144.5) are oxygenated, similar to ring C of compounds **4**–**7**. The γ,γ-dimethylallyl substituent was placed at C-5′ (δ_C_ 131.3) based on the HMBC correlations (Table 8, Figure S72, Supporting Information) of H_2_-1″ (δ_H_ 2.96) to C-4′ (δ_C_ 144.5), C-5′ (δ_C_ 131.3), and C-6′ (δ_C_ 112.4) and the NOEs between H-6′ (δ_H_ 6.75) and H-2″ (δ_H_ 5.12). A dihydropyran ring was placed at C2′/C-3′ based on the HMBC correlation of H-4″′ (δ_H_ 2.32) to C-3′ (δ_C_ 139.0) and C-2′ (δ_C_ 119.7), and the hydroxy group at C-4′ based on the HMBC correlation of 4′-OH with C-3′ (δ_C_ 139.0), C-4′(δ_C_ 144.5), and C-6′(δ_C_ 112.4). It was further confirmed by the NOEs of H-8 (δ_H_ 7.45) to H-4″′ (δ_H_ 2.32). Based on the spectroscopic evidence, this compound, usambarin H (**8**), was characterized as 5-(7-hydroxynaphthalen-2-yl)-2,2-dimethyl-7-(3-methylbut-2-en-1-yl)chroman-8-ol.

**Table 8:** NMR Spectroscopic Data (500 MHz, CDCl_3_) for Usambarin H (**8**)

position	δ_C_, type	δ_H_ (*J* in Hz)	HMBC^a^
1	109.6 CH	7.10 d (2.0)	2, 3
2	153.7 C		
3	117.7 CH	7.13 d (9.5)	2
4	129.8 CH	7.78 dd (8.5, 2.3)	2, 4a, 5
4a	134.8 C		
5	127.7 CH	7.78 dd (8.5, 2.3)	1, 4a, 6
6	126.5 CH	7.11 d (5.8)	1′, 5
7	138.5 C		
8a	127.9 C		
8	127.2 CH	7.45 d (1.6)	1, 1′, 8a, 6
1′	132.6 C		
2′	119.7 C		
3′	139.0 C		
4′	144.5 C		
5′	131.3 C		
6′	112.4 CH	6.75 s	1′, 1″, 4′, 3′
1″	32.0 CH_2_	2.96 d (7.4)	2″, 3″, 6′, 5′
2″	123.9 CH	5.12 m	
3″	131.6 C		
4″	17.7 CH_3_	1.33 s	3″, 5″
5″	25.9 CH_3_	1.62 d (1.6)	2″, 3″, 4″
2″′	74.8 C		
3″′	33.2 CH_2_	1.68 t (6.8)	2′, 2″′, 4″′, 2″′Me
4″′	21.9 CH_2_	2.32 m	1′, 2′, 2″′, 3′, 3″′
2″′-Me_2_	27.0 CH_3_	1.34 s	2″′, 3″′
2″′-Me	27.0 CH_3_	1.34 s	2″′
2-OH		4.94 s	
4′-OH		5.60 s	3′, 4′, 6′

a HMBC correlations, optimized for 6 Hz, are from stated proton(s) to the indicated carbon.

Compound **9** was isolated as a white solid from the stem of *S. usambarensis* and was given the molecular formula of C_16_H_14_O_4_ based on HRESIMS (*m*/*z* 271.0970 [M + H]^+^, calcd 271.0965, Figure S83, Supporting Information) analysis. Its NMR (Table 9, Figures S76–S82, Supporting Information) data suggested a flavan-2-ene skeleton with its ring A having *ortho-*coupled (*J* = 8.3 Hz) protons resonating at δ_H_ 6.97 (H-5) and δ_H_ 6.72 (H-6) and the corresponding carbons appearing at δ_C_ 114.0 (C-5) and 113.0 (C-6). This ring is substituted at C-7 (δ_C_ 145.9) and C-8 (δ_C_ 132.4) with methoxy (δ_H_ 3.89; δ_C_ 60.1) and hydroxy groups. The deshielding of the methoxy carbon (δ_C_ 60.1) is typical of di-*ortho*-substitution, and hence is consistent with its placement at C-8, rather than at C-6 or C-7. The placement of 8-OMe was confirmed by the HMBC correlation of the methoxy protons (δ_H_ 3.89) with C-8 (δ_C_ 132.4) and the NOEs of 7-OH (δ_H_ 9.06) to H-6 (δ_H_ 6.72) and of 8-OMe (δ_H_ 3.89) to H-2′/6′ (δ_H_ 7.10). Ring B showed the presence of an AA′XX′ spin system, appearing at δ_H_ 7.10 (H-2′/6′) and 6.71 (H-3′/5′) with their carbon atoms resonating at δ_C_ 129.6 (C-2′/6′) and 115.2 (C-3′/5′) with a hydroxy placed at C-4′ (δ_C_ 155.9). The ring C protons resonate at δ_H_ 6.35 (br d, *J* = 1.0 Hz, H-3) and δ_H_ 3.95 (br s, CH_2_-4), and the corresponding carbons at 102.9 (C-3) and 33.1 (C-4). The C-2 to C-1′ connection is confirmed by the NOE of H-3 (δ_H_ 6.35) to H-2′/6′ (δ_H_ 7.10). Based on the spectroscopic evidence, this new flav-2-ene, usambarin J (**9**), was characterized as 2-(4-hydroxyphenyl)-8-methoxy-4*H*-chromen-7-ol.

**Table 9:** NMR Spectroscopic Data (600 MHz, DMSO-*d*_6_) for Usambarin J (**9**)

position	δ_C_, type	δ_H_ (*J* in Hz)	HMBC^a^
2	157.1 C		
3	102.9 CH	6.35 br d (1.0)	2, 4a, 8a
4	33.1 CH	3.95 s	1′, 2, 3, 8
4a	122.3 C		
5	114.1 CH	6.97 d (8.3)	3, 4a, 8, 8a
6	113.0 CH	6.72 d (8.3)	4a, 7, 8
7	145.9 C		
8a	147.0 C		
8	132.4 C		
1′	127.6 C		
2′/6′	129.6 CH	7.10 AA′	3′, 4′, 5′, 6′
3′/5′	115.2 CH	6.71 XX′	1′, 5′
4′	155.9 C		
7-OH		9.06 s	5, 6, 7, 8
4-′OH		9.26 s	3′, 4′, 5′
8-OMe	60.1	3.89 s	8

a HMBC correlations, optimized for 6 Hz, are from stated proton(s) to the indicated carbon.

Compound **10** was obtained as a white solid. Its HREIMS spectrum was compatible with the molecular formula C_23_H_22_O_4_ (*m*/*z* 285.1127 [M + H]^+^, calcd 285.1121, Figure S92, Supporting Information). Its NMR data (Table 10, Figures S85–S91, Supporting Information) were typical of a phenyl-1-benzoxepin derivative.^19^ The ring A has an AMX spin system with protons resonating at δ_H_ 7.11 (d, *J* = 8.3 Hz, H-6), 6.52 (dd, *J* = 8.4, 2.6 Hz, H-7), and 6.47 (d, *J* = 2.6 Hz, H-9) and the corresponding carbons at δ_C_ 133.8 (C-6), 110.1 (C-7), and 107.0 (C-9) and a hydroxy substituent connected to C-8 (δ_C_ 155.7) based on its (δ_H_ 4.75) HMBC cross-peaks to C-7 (δ_C_ 110.1), C-8 (δ_C_ 155.7), and C-9 (δ_C_ 107).

**Table 10:** NMR Spectroscopic Data (500 MHz, CDCl_3_) for Usambarin K (**10**)

position	δ_C_, type	δ_H_ (*J* in Hz)	HMBC^a^
2	75.4 CH_2_	4.29 ddd (11.7, 3.2, 1.0)	1′, 4, 9a
		4.14 dd (11.8, 6.7)	1′, 4, 9a
3	49.9 CH	3.90 dd (5.5, 3.2)	
4	130.7 CH	5.87 dd (11.8, 4.0)	1′, 2, 3, 5a
5a	120.0 C		
5	128.1 CH	6.40 dd (11.8, 2.0)	3, 6, 9a,
6	134.1 CH	7.11 d (8.3)	5, 8, 9a,
7	110.1 CH	6.52 dd (8.4, 2.6)	5a, 8, 9
8	155.7 C		
9a	160.5 C		
9	107.0 CH	6.47 d (2.6)	5a, 7, 8, 9a
1′	133.1 C		
2′	110.9 CH	6.72 d (2.0)	1′, 3, 3′, 4′, 6′
3′	146.6 C		
4′	144.8 C		
5′	114.5 CH	6.87 d (8.0)	1′, 3′, 4′
6′	121.3 CH	6.75 dd (8.0, 2.0)	2′, 3, 4′
3′-OH		5.52 s	2′, 3′, 4′
8-OH		4.75 s	7, 8, 9
4′-OMe	56.1 CH_3_	3.84 s	4′

a HMBC correlations, optimized for 6 Hz, are from stated proton(s) to the indicated carbon.

Ring B is disubstituted with hydroxy and methoxy groups and has an AXY spin system with protons resonating at δ_H_ 6.87 (d, *J* = 8.0 Hz, H-5′), 6.75 (dd, *J* = 2.0, 8.0 Hz, H-6′), and 6.72 (d, *J* = 2.0 Hz, H-2′) and the corresponding carbons at δ_C_ 121.3 (C-6′), 114.3 (C-5′), and 110.8 (C-2′). The HMBC cross-peaks of 4′-OH (δ_H_ 5.52) to C-3′ (δ_C_ 146.6), C-4′ (δ_C_ 144.8), and C-5′ (δ_C_ 114.5) suggested the placement of the hydroxy substituent at C-4′ (δ_C_ 144.8). The location of the methoxy group (δ_H_ 3.84 and δ_C_ 56.1) at C-3′ (δ_C_ 146.6) was established by the HMBC correlation of its protons to C-3′ (δ_C_ 146.6) and its NOE correlation to H-2′ (δ_H_ 6.72). HMBC correlation of the signal at δ_H_ 6.72 (H-2′) with C-3 (δ_C_ 49.9) confirmed the connection of ring B to ring C. Ring C possesses two sp^3^-hybridized carbons, C-2 (δ_C_ 75.5) and C-3 (δ_C_ 49.9), and two sp^2^-hybridized ones, C-4 (δ_C_ 130.3) and C-5 (δ_C_ 127.7). Its connection to ring A via the bridging C9a and C5a atoms is revealed by the HMBC correlations of CH_2_-2 (δ_H_ 4.14 and 4.29) to C-9a (δ_C_ 160.5) and of H-5 (δ_H_ 6.40) to C-9a (δ_C_ 160.5) and C-6 (δ_C_ 134.1), whereas to ring B via the C3–C1′ bond by the HMBC cross-peaks of CH_2_-2 (δ_H_ 4.14 and 4.29) to C-1′ (δ_C_ 133.1) and of H-2′ (δ_H_ 6.72) and H-6′ (δ_H_ 6.75) to C-3 (δ_C_ 49.9). The absolute configuration at C-3 was not determined. Based on the above spectroscopic evidence, this new compound, usambarin K (**10**), was characterized as 3-(3-methoxy-4-hydroxy)-2,3-dihydrobenzo[*b*]oxepin-8-ol.

The major constituents of *S*. *usambarensis* were evaluated for antibacterial activity against the Gram-negative *E*. *coli* and the Gram-positive *B*. *subtilis* as well as for cytotoxicity against MCF-7 human breast cancer cells (Figure S85 and Table S3, Supporting Information). Out of the tested compounds (Table S3, Supporting Information), usambarin D (**4**) showed moderate antibacterial activity (MIC = 9.0 μM) against *B. subtilis*, while usambarins A (**1**) and B (**2**) were not toxic (Figure S85, Supporting Information).

In conclusion, 12 natural products including the three new benzo[*b*]naphtho[2,1-*d*]furans **1**–**3**, the five new 2-phenylnaphthalene derivatives **4**–**8**, the new flavan **9**, and the new phenyl-1-benzoxepin **10** were isolated and characterized from the CH_2_Cl_2_/MeOH (1:1) extracts of the stem and roots of *S. usambarensis*. The benzo[*b*]naphtho[2,1-*d*]furan skeletons of **1**–**3** are so far unprecedented in natural products, and the 2-phenylnaphthalene skeletons of **4**–**8** have previously only scarcely been reported.^20,21^ Usambarin D (**4**) showed moderate antibacterial activity against *B. subtilis* with no significant cytotoxicity, while usambarins A (**1**) and B (**2**) were not cytotoxic against MCF-7 human breast cancer cells.

## Experimental Section

### General Experimental Procedures

UV spectra were measured using a Shimadzu UV-1650 PC UV/vis spectrophotometer. NMR spectra were acquired on a Bruker Avance NEO 500 MHz (TXO cryogenic probe) or a 600 MHz (TCI cryogenic probe) spectrometer and were processed using the MestreNova (v14.0.0) software referencing the chemical shifts to the residual solvent signals (CDCl_3_: δ_H_ 7.26, δ_C_ 77.16; CD_3_OD: δ_H_ 3.31, δ_C_ 49.0; DMSO-*d*_6_: δ_H_ 2.50, δ_C_ 39.5). TLC analysis was performed on Merck precoated silica gel 60 F_254_ aluminum plates using UV detection at 254 and 365 nm. Column chromatography was done on silica gel 60 (230–400 mesh) and on Sephadex LH-20 (GE Healthcare). Preparative reversed-phase HPLC separations were performed on a Waters 600E system using Chromulan v. 0.88 (Pikron Ltd.) software and an RP-C8 Kromasil column (250 mm × 25 mm, 5 μm) or on an Interchim Ultra Performance Flash Purification (PF-430) system using Interchim v 5.1d.02 software and the same RP-C8 Kromasil column. HRESIMS spectra were acquired with a Q-TOF LC/MS spectrometer with a lockmass-ESI source (Stenhagen Analysis Lab AB, Gothenburg, Sweden), using a 2.1 × 30 mm, 1.1 μm RP-C18 column and H_2_O/MeCN gradient (5:95 to 95:5, with 0.2% HCOOH). Mass spectra were acquired on a Waters Micromass ZQ Multimode Ionization ESCI using LC-MS in ESI mode, connected to an Agilent 1100 series gradient pump system and an RP-C18 Atlantis T3 column (3.0 × 50 mm, 5 μm), using Milli-Q water/MeCN (5:95 to 95:5, with 1% HCO_2_H, flow rate 0.75 mL/min over 6 min).

### Plant Materials

The stem and roots of *Streblus usambarensis* were collected from the Gondoni forest (4°24′38.2″ S, 39°28′34.5″ E, altitude: 40 m), Kwale County, in July 2016. The plant was authenticated by Mr. Pactrick C. Mutiso of the University Herbarium, Department of Biology, University of Nairobi, where a voucher specimen (PBC 2016/008) was deposited.

### Extraction and Isolation

Ground roots of *S. usambarensis* (900 g) were extracted with CH_2_Cl_2_/MeOH (1:1) at room temperature to yield a crude extract (98.8 g) after concentration. The crude extract was loaded on a silica gel (500 g) column, eluted with *iso*-hexane containing increasing amounts of EtOAc (1% to 80% v/v), and pooled into 21 fractions. Three fractions (RF1, RF2, and RF3) that were eluted with 3% EtOAc were washed separately with *iso*-hexane, giving compound **3** (15 mg) as a white amorphous solid, compound **1** (25 mg) as a colorless solid, and compound **12** (19 mg) as colorless solids, respectively. The fraction that was eluted with 8% EtOAc was separated using prep-HPLC (MeOH/H_2_O, gradient elution 5–95% MeOH), yielding compound **2** (8.0 mg). Ground stems of *S. usambarensis* (965 g) were extracted with CH_2_Cl_2_/MeOH (1:1) at room temperature. The crude extract (91 g) was partitioned between H_2_O and EtOAc. The EtOAc layer was concentrated to give a crude extract (45 g). The EtOAc extract was loaded on a silica gel (500 g) column and eluted with *iso-*hexane containing increasing amounts of EtOAc (1% to 99% v/v). The eluents were then pooled into 24 fractions (SF1–SF24). Two fractions that were eluted with 5% EtOAc were subjected to column chromatography over Sephadex (eluting with CH_2_Cl_2_/MeOH, 1:1) followed by purification on preparative HPLC (MeOH/H_2_O, gradient elution 5–95% MeOH), giving compound **8** (6.5 mg) as a white solid and compound **11** (12.7 mg) as colorless needles. Fractions that were eluted with 8% EtOAc were subjected to column chromatography over Sephadex (CH_2_Cl_2_/MeOH, 1:1), followed by purification on preparative HPLC (MeOH/H_2_O, gradient elution 5–90% MeOH), giving compound **4** (20.5 mg) as white crystals and compound **7** (9 mg) as a white solid. The fraction that was eluted with 8% EtOAc was subjected to Sephadex (eluting with CH_2_Cl_2_/MeOH, 1:1) and further purification on preparative HPLC (MeOH/H_2_O, gradient elution 5–90% MeOH) to provide compound **9** (6.4 mg). The fraction that was eluted with 10% EtOAc was subjected to column chromatography over Sephadex (CH_2_Cl_2_/MeOH, 1:1) and further purified on preparative HPLC (MeOH/H_2_O, gradient elution 5–90% MeOH) to give compound **10** (6.5 mg) as a white paste. The fraction that was eluted with 15% EtOAc was purified by preparative HPLC (MeOH/H_2_O, gradient elution 5–90% MeOH) to give colorless solids of compound **5** (10 mg) and **6** (7.3 mg).

#### Usambarin A (**1**):

white solid (CDCl_3_); UV (MeOH) λ_max_ (log ε) 270 nm (4.0), 310 nm (4.1); ^1^H NMR (Table 1); ^13^C NMR (Table 1); HRESIMS [M + H]^+^*m*/*z* 363.1596 (calcd for C_23_H_23_O_4_, 363.1591).

#### Usambarin B (**2**):

white solid; UV (MeOH) λ_max_ (log ε) 273 nm (4.0), 321 nm (4.0); ^1^H NMR (Table 2); ^13^C NMR (Table 2); HRESIMS [M + H]^+^*m*/*z* 349.1440 (calcd for C_22_H_21_O_4_, 349.1434).

#### Usambarin C (**3**):

white amorphous solid; UV (MeOH) λ_max_ (log ε) 270 nm (4.0), 350 nm (4.1); ^1^H NMR (Table 3); ^13^C NMR (Table 3); HRESIMS [M + H]^+^*m*/*z* 347.1283 (calcd for C_22_H_19_O_4_, 347.1278).

#### Usambarin D (**4**):

white solid; UV (MeOH) λ_max_ (log ε) 230 nm (3.9), 255 nm (3.9); ^1^H NMR (Table 4); ^13^C NMR (Table 4); HREIMS [M + H]^+^*m*/*z* 319.1334 (calcd for C_21_H_19_O_3_, 319.1329).

#### Usambarin E (**5**):

white solid; UV (MeOH) λ_max_ (log ε) 234 nm (3.9), 262 nm 3.9), 290sh nm (4.0); ^1^H NMR (Table 5) Table 4; ^13^C NMR (Table 5); EIMS [M – H]^−^*m*/*z* 319.2 (calcd for C_21_H_21_O_3_, 321.1485).

#### Usambarin F (**6**):

white solid; UV (MeOH), λ_max_ (log ε) 234 nm (3.9); ^1^H NMR (Table 6); ^13^C NMR (Table 6); HREIMS [M + H]^+^*m*/*z* 335.1647 (calcd for C_22_H_23_O_3_, 335.1642).

#### Usambarin G (**7**):

white solid; UV(MeOH), λ_max_ (log ε) 231 nm (3.9), 257 nm (3.9); ^1^H NMR (Table 7); ^13^C NMR (Table 7); HREIMS [M + H]^+^*m*/*z* 335.1647 (calcd for C_22_H_23_O_3_, 335.1642.

#### Usambarin F (**8**):

white solid; ^1^H NMR (Table 8); ^13^C NMR (Table 8); HREIMS [M + H]^+^*m*/*z* 389.2117 (calcd for C_26_H_29_O_3_, 389.2111).

#### Usambarin J (**9**):

white solid; ^1^H NMR (Table 9); ^13^C NMR (Table 9); HREIMS [M + H]^+^*m*/*z* 271.0970 (calcd for C_16_H_15_O_4_, 271.0965).

#### Usambarin K (**10**):

white solid; ^1^H NMR (Table 10); ^13^C NMR (Table 10); HREIMS [M + H]^+^*m*/*z* 285.1127 (calcd for C_17_H_17_O_4_, 285.1121).

### X-ray Diffraction Analysis

Single crystals were obtained by slow solvent evaporation. Single crystals were mounted on a fiber loop and fixated using Fomblin oil. The data were collected at 150(2) K on a Bruker D8 APEX-II equipped with an APEX-II CCD camera using Mo Kα radiation (λ = 0.71073 Å). Data reduction was performed with SAINT,^22^ and absorption corrections for the area detector were performed using SADABS.^23^ Structures were solved by direct methods and refined by least-squares methods on *F*^2^ using the SHELX and the OLEX2^24^ software suits, respectively. Non-hydrogen atoms were refined anisotropically. Hydrogen atoms were constrained in geometrical positions to their parent atoms. The X-ray structure (cif) data of **1** (CCDC 2236402) and **4** (CCDC 2236403) have been deposited with the Cambridge Crystallographic Data Centre. Copies of the data can be obtained, free of charge, on application to the Director, CCDC, 12 Union Road, Cambridge CB2 1EZ, UK (fax: + 44-(0)1223-336033 or e-mail: deposit@ccdc.cam.ac.uk). Further details of the X-ray data acquisition are given in the Supporting Information.

### Antibacterial Assay

The major constituents were assessed for antibacterial assay against *E. coli* and *B. subtilis* following the procedures described by Kalenga et al.^25^ Initially, each sample was dissolved in 100% DMSO to constitute a 1 mg/mL solution and subsequently kept at −20 °C. The culturing of bacterial strains followed the standard protocols described by Muller et al.^17^ with minor modifications. Briefly, bacterial cultures were allowed to grow in Mueller-Hinton broth for 24 h to an optical density (OD) = 0.5 (λ = 540 nm). A 10-fold dilution of the broth bacterial suspension was then performed. The samples were incorporated into the medium to constitute a concentration of 35 μg/mL. A prewarmed (100 μL) medium with the samples as DMSO solution was added into a 96-well microplate, incubated at 37 °C without shaking, for 20–4 h. The resazurin assay for assessing viability was then performed as described by Sarker et al.^26^ Accordingly, an Alamar Blue staining solution (10 μL) was introduced per well continuously for 1 h at a constant temperature of 37 °C. The fluorescence emitted by the viable cells was determined using POLARstar Omega (BMG Labtech, Cape Town, South Africa) set at excitation λ = 540 nm and emission filter λ = 590 nm. As a positive control, a standard antibiotic, ampicillin, was used (Figure S86, Supporting Information), while DMSO was applied as a negative control. The bleed-through between the wells was controlled by leaving an empty well in-between (thus 384-well plates were considered for this purpose). These assays were performed in triplicates. Minimum inhibitory concentrations (MIC) and effective concentrations (EC) were determined using an EC_90_ calculator webtool (AAT Bioquest, Inc.) and the Quest Graph EC_50_.

### Cytotoxicity Assay

MCF-7 cells were used to evaluate the cytotoxic effect of the isolated constituents, following the protocol by Koudokpon et al.^27^ Briefly, the cells were cultured and kept in exponential growth in a modified medium, as described by Umereweneza et al.^28^ PrestoBlue was used to determine the cell viability (Thermo Fisher) for a 24 h incubation period as per the manufacturer’s recommendations. The fluorescence from resorufin was determined and measured using POLAR star Omega (BMG Labtech) set at excitation λ = 540 nm and emission filter λ = 590 nm. Cell viability, EC_90_, and EC_50_ values for each compound were determined as described by Umereweneza et al.^28^

## Data Availability

The original FIDs for compounds **1**–**12** are freely available on Zenodo with DOI: 10.5281/zenodo.7213520.

## Supporting Information Available

NMR and MS data for the isolated compounds and antibacterial and cytotoxicity data (PDF)
X-ray crystallographic data for compound **1** (CIF)
X-ray crystallographic data for compound **4** (CIF)

## Supplementary Material

np3c00051_si_001.pdf

np3c00051_si_002.cif

np3c00051_si_003.cif
