# Supplementary material for: Benzo[b]naphtho[2,1-d]furans and 2-Phenylnaphthalenes from Streblus usambarensis
Source: J Nat Prod. 2023 Apr 12;86(4):1010–8. doi: 10.1021/acs.jnatprod.3c00051 (PMC10152483; doi:10.1021/acs.jnatprod.3c00051)
Supplement: Supplementary file 1 — np3c00051_si_001.pdf [file np3c00051_si_001.pdf]

SUPPORTING INFORMATION

Benzo[*b*]naphtho[2,1-*d*]furans and 2-phenylnaphthalenes from *Streblus usambarensis*

Carolyne Chepkirui, Fozia Ali Adem, Anastasia Rudenko, Yukino Gütlin, Albert Ndakala, Solomon Derese, Andreas Orthaber, Catarina Bourgard, Abiy Yenesew, Máté Erdélyi

Table of Contents

Spectroscopic data of Usambarin A (**1**) S2

Spectroscopic data of Usambarin B (**2**).......... S7

Spectroscopic data of Usambarin C (**3**).......... S12

Spectroscopic data of usambarin D (**4**) S16

Spectroscopic data of Usambarin E (**5**).......... S21

Spectroscopic data of Usambarin F (**6**) S26

Spectroscopic data of Usambarin G (**7**) S31

Spectroscopic data of Usambarin H (**8**) S35

Spectroscopic data of Usambarin J (**9**).......... S40

Spectroscopic data of Usambarin K (**10**) S44

Physical and spectroscopic data S48

Single Crystal X-ray Diffraction Analysis S53

Antibacterial activity and cytotoxicity S55

The original NMR spectra (FID and MNova files) for compounds **1-12** are freely available on Zenodo as 10.5281/zenodo.7213520.

Spectroscopic data of Usambarin A (1)

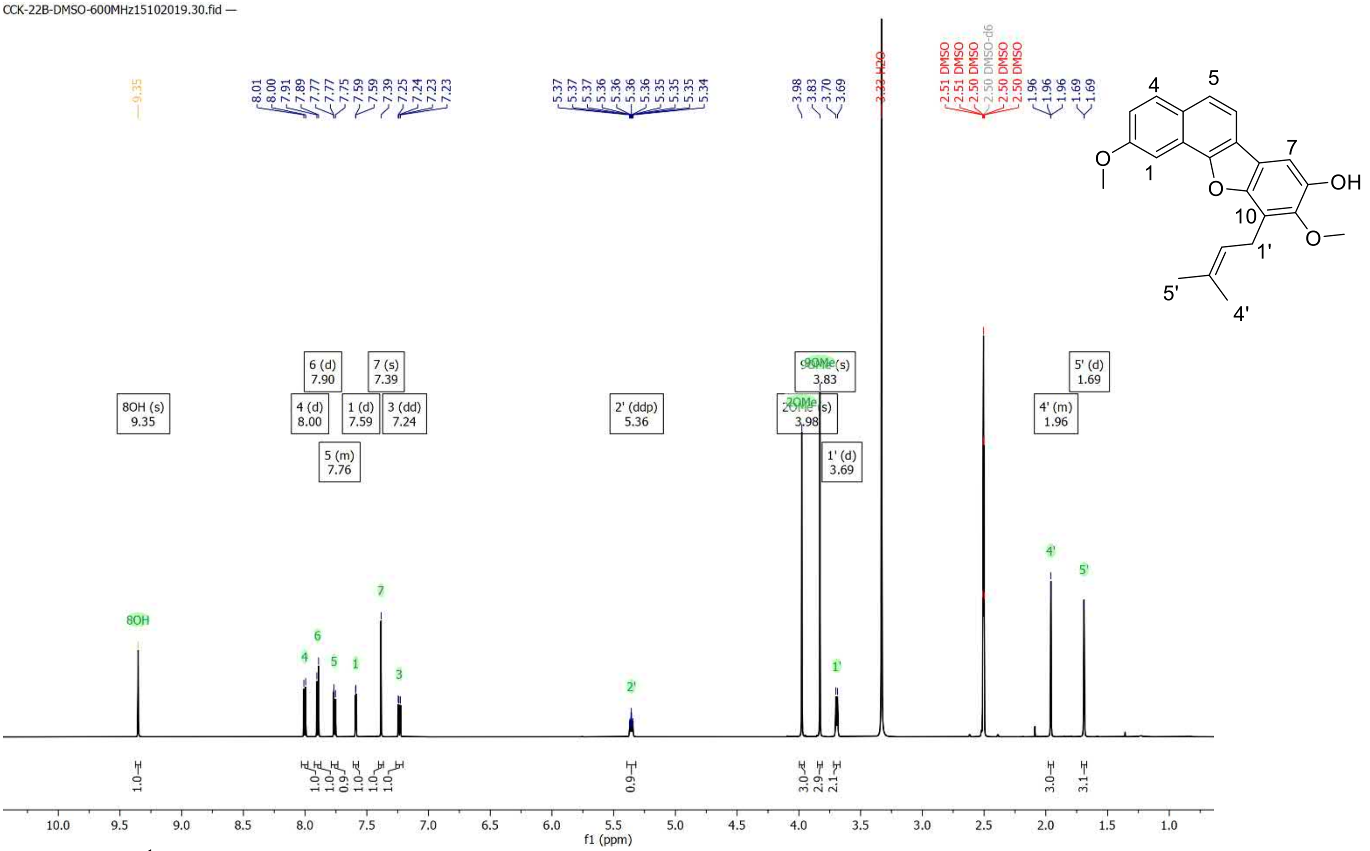

Figure S1. ^{1}H NMR (600 MHz, DMSO, 25°C) spectrum of usambarin A (**1**)

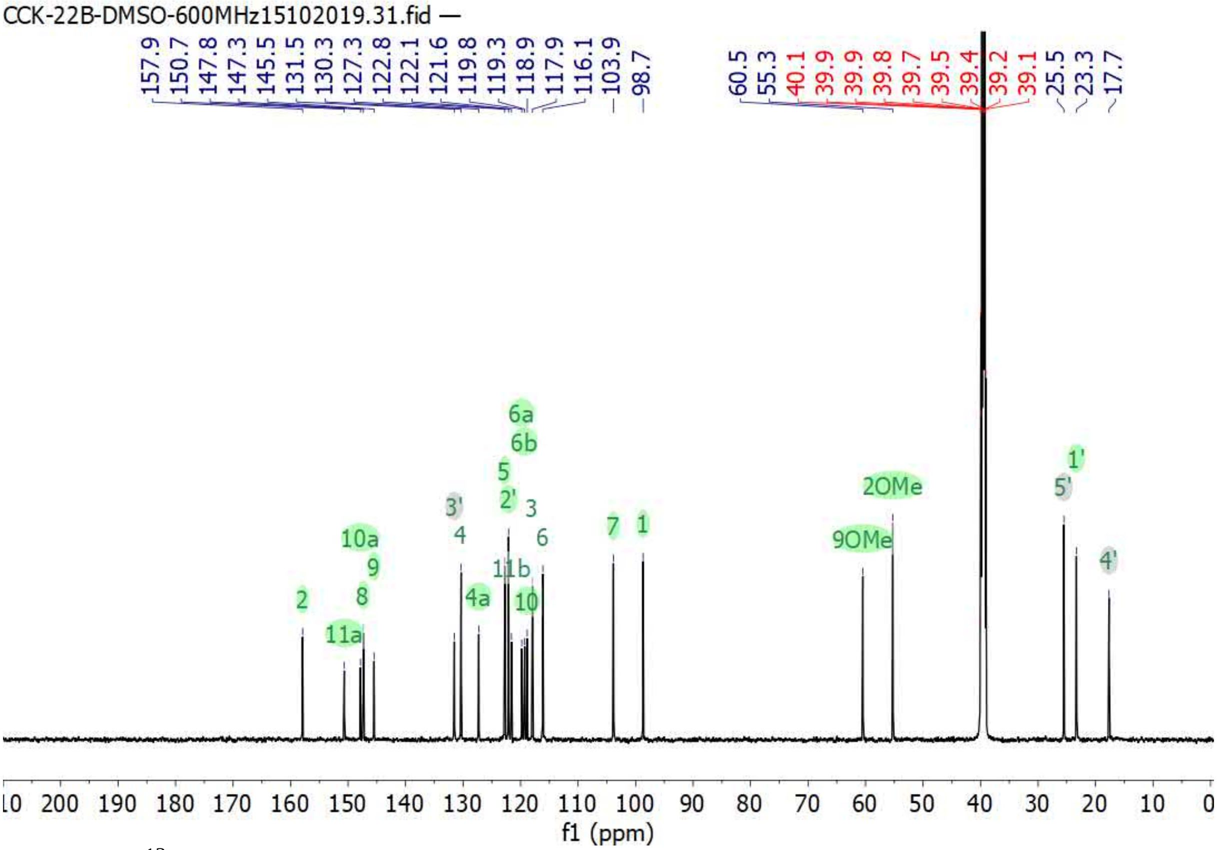

Figure S2. ^{13}C NMR (150 MHz, DMSO, 25°C) spectrum of usambarin A (**1**)

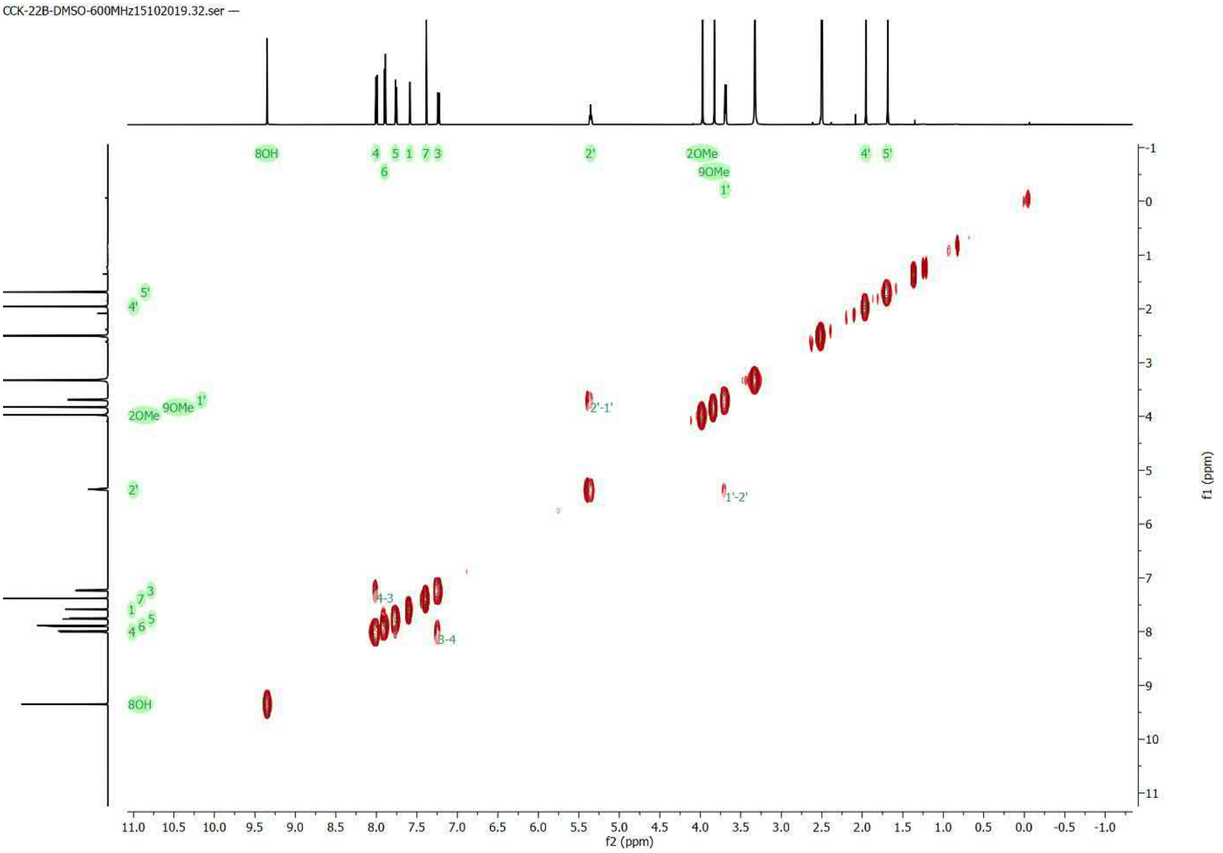

Figure S3. COSY (600 MHz, DMSO, 25°C) spectrum of usambarin A (**1**)

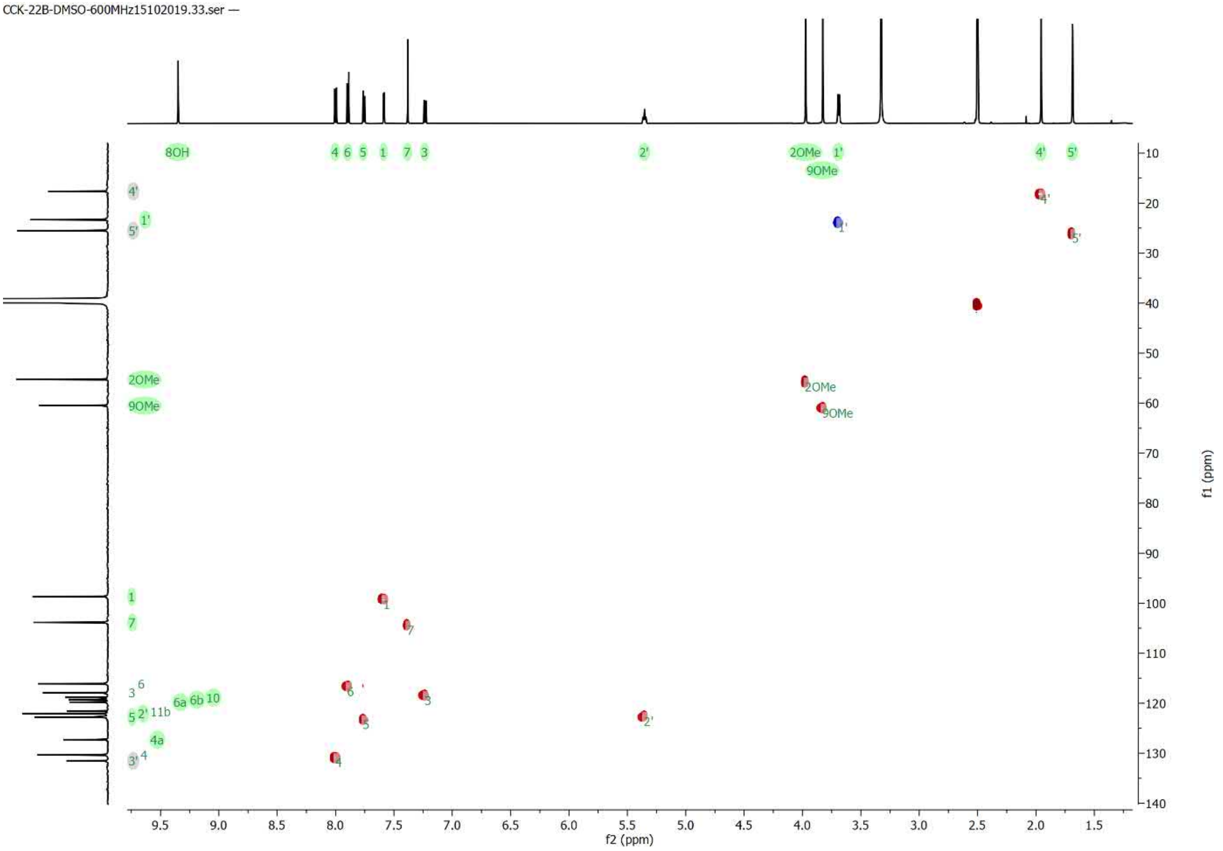

Figure S4.HSQC (600 MHz, DMSO, 25°C) spectrum of usambarin A (**1**)

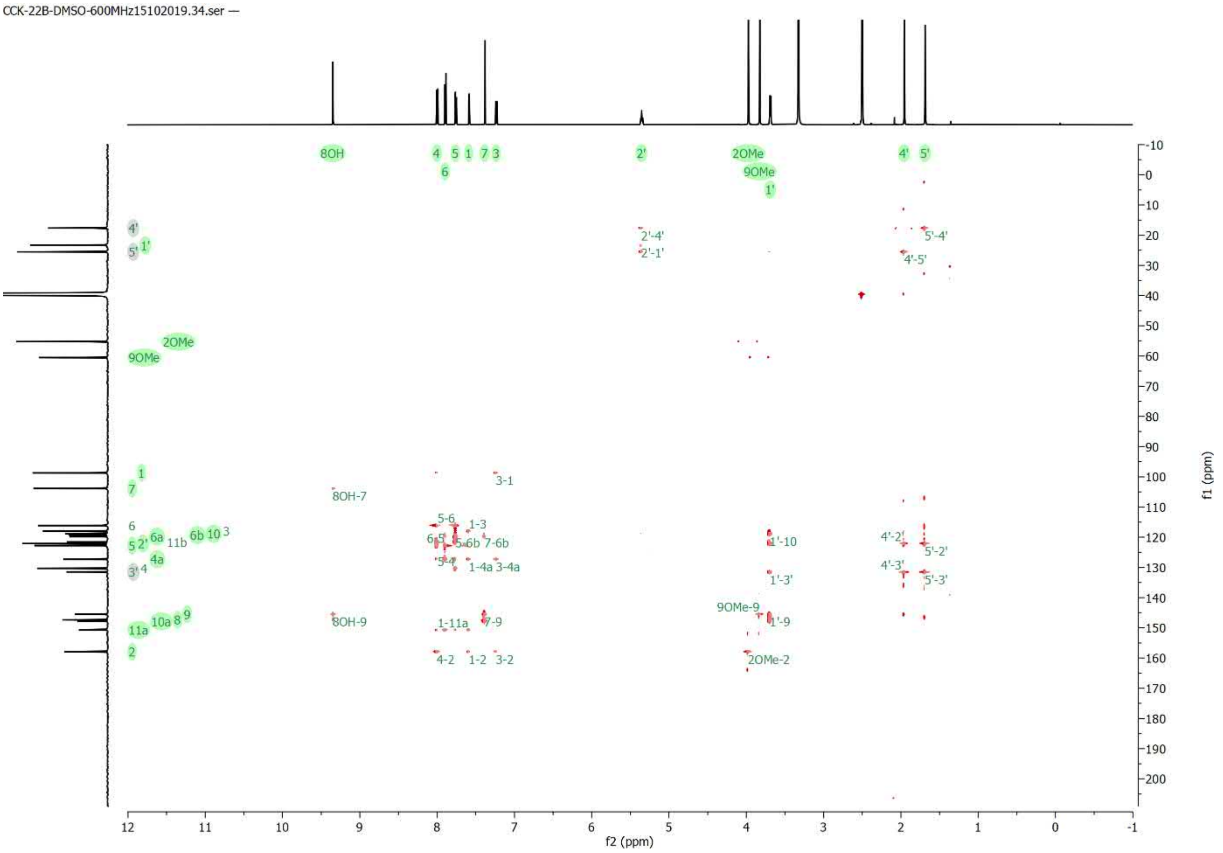

Figure S5.HMBC (600 MHz, DMSO, 25°C) spectrum of usambarin A (**1**)

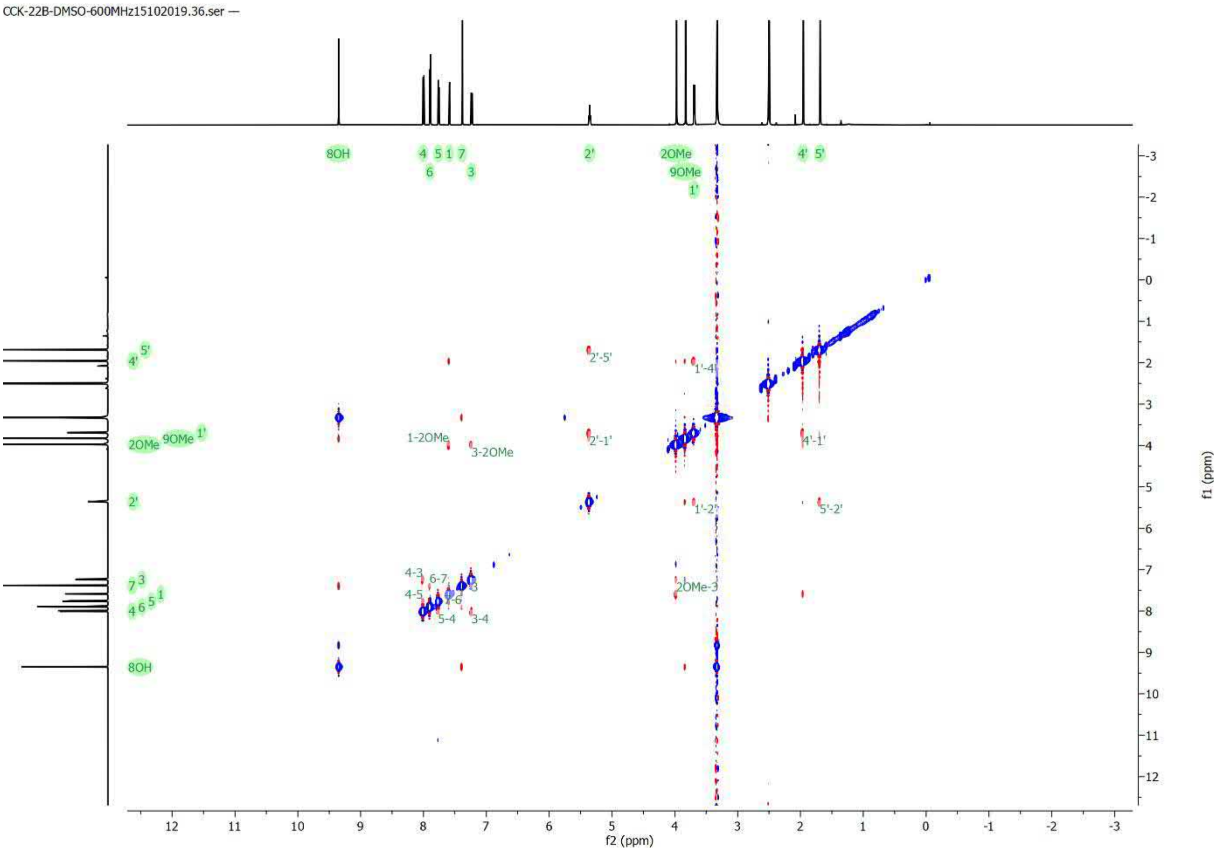

Figure S6. NOESY (600 MHz, DMSO, 25°C) spectrum of usambarin A (**1**)

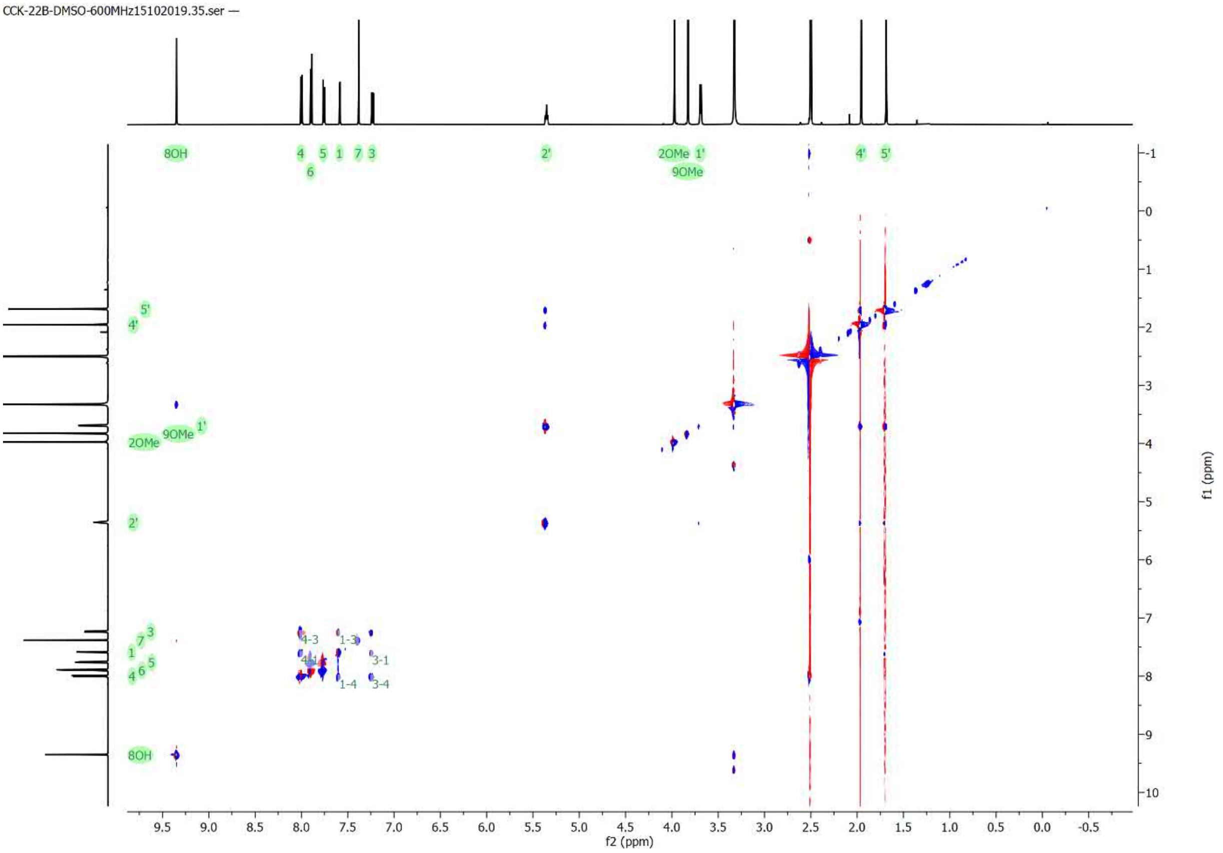

Figure S7.TOCSY (600 MHz, DMSO, 25°C) spectrum of usambarin A (**1**)

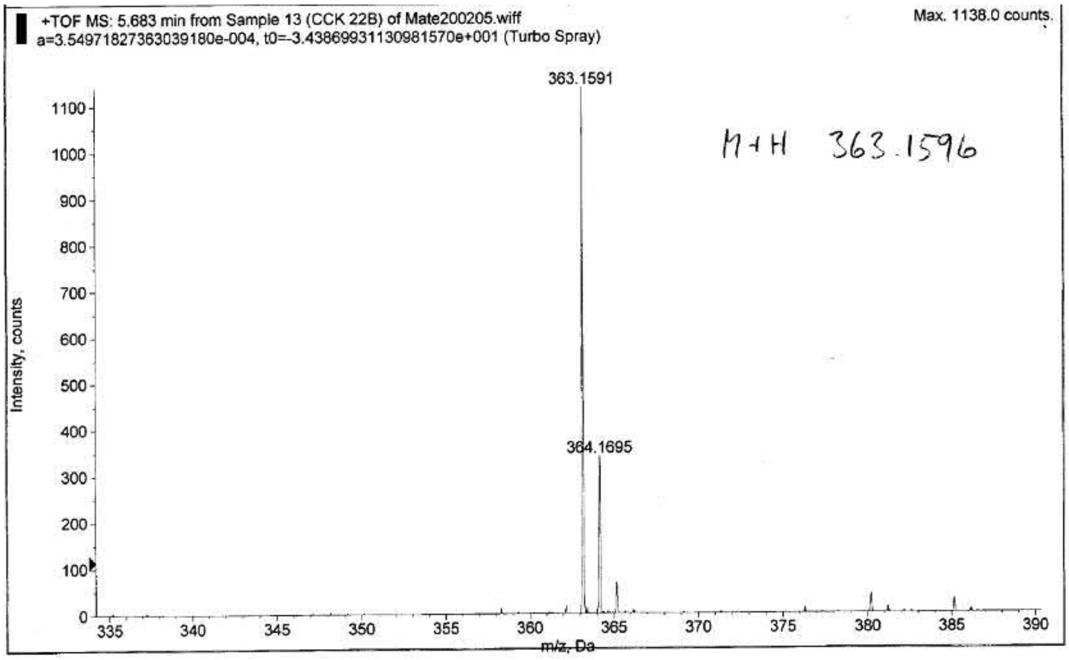

Figure S8. HRMS spectrum of usambarin A (**1**)

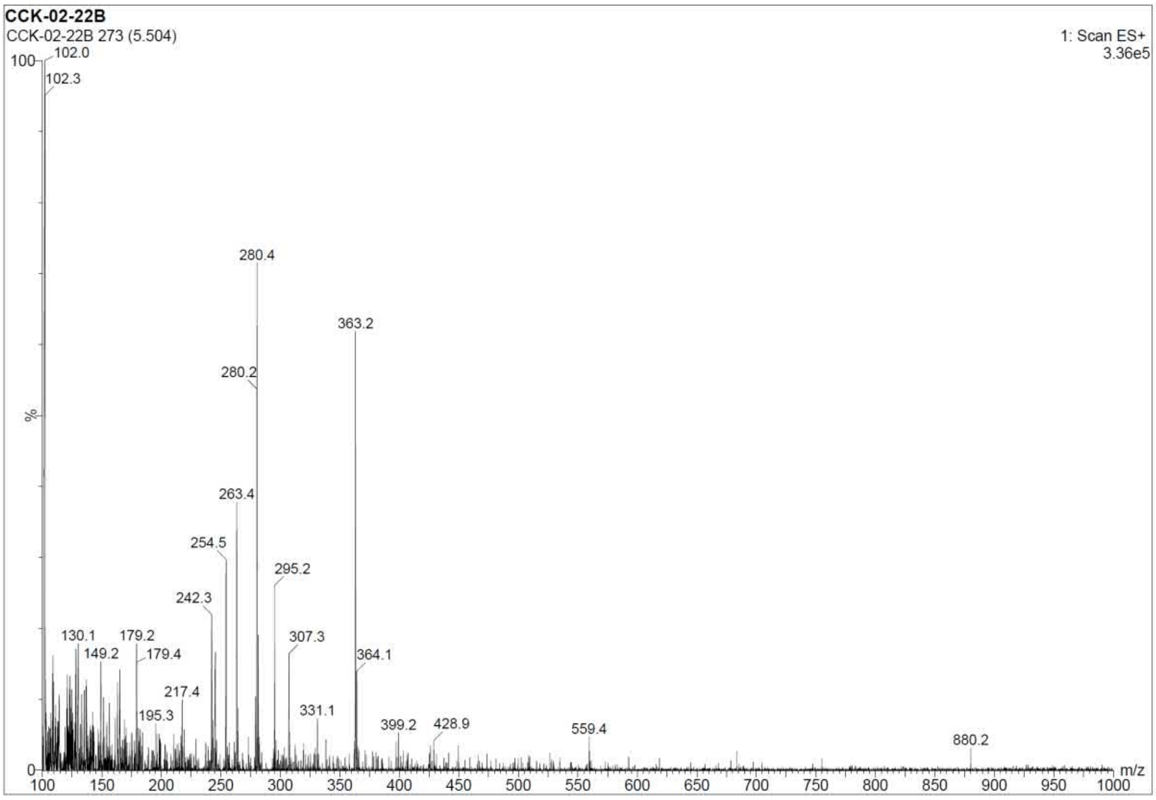

Figure S9. ESI-MS spectrum of usambarin A (**1**)

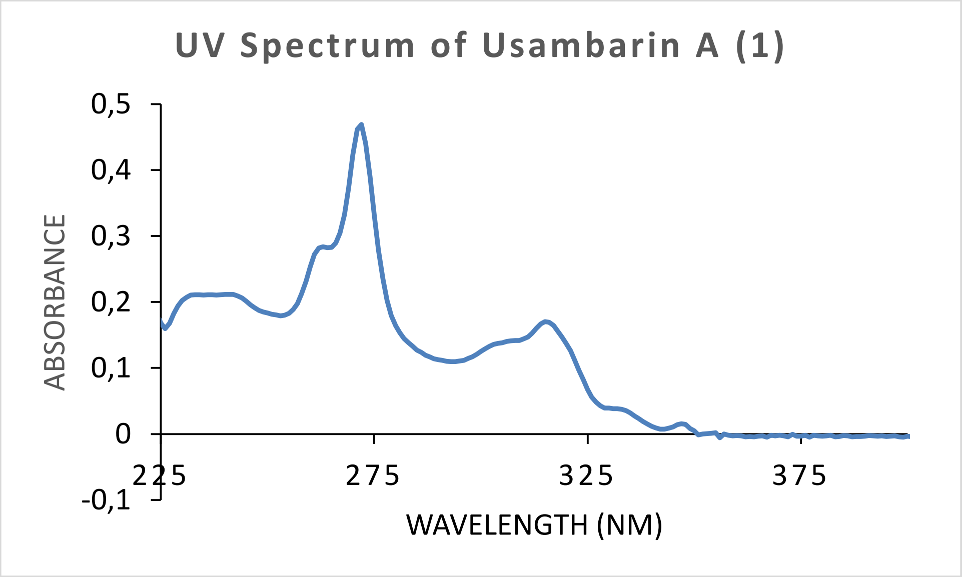

Figure S10. UV spectrum (MeOH) of usambarin A (**1**)

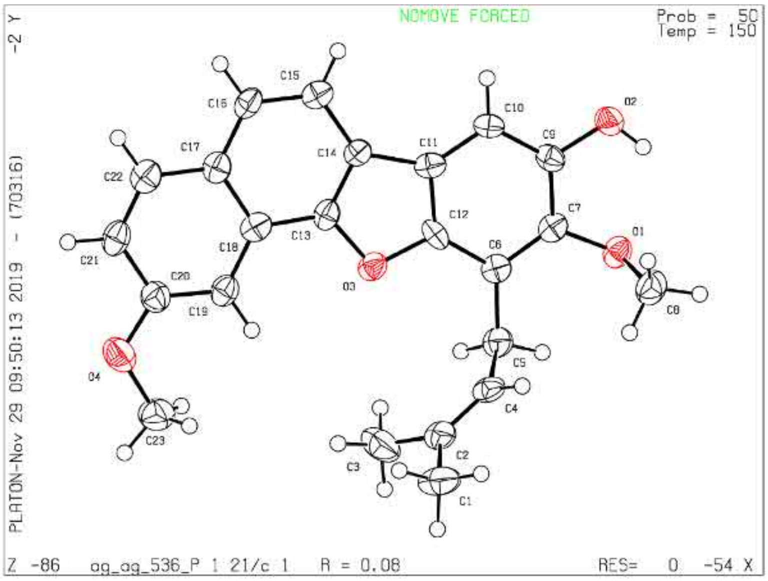

Figure S11. X-ray structure of usambarin A (**1**)

Spectroscopic data of Usambarin B (2)

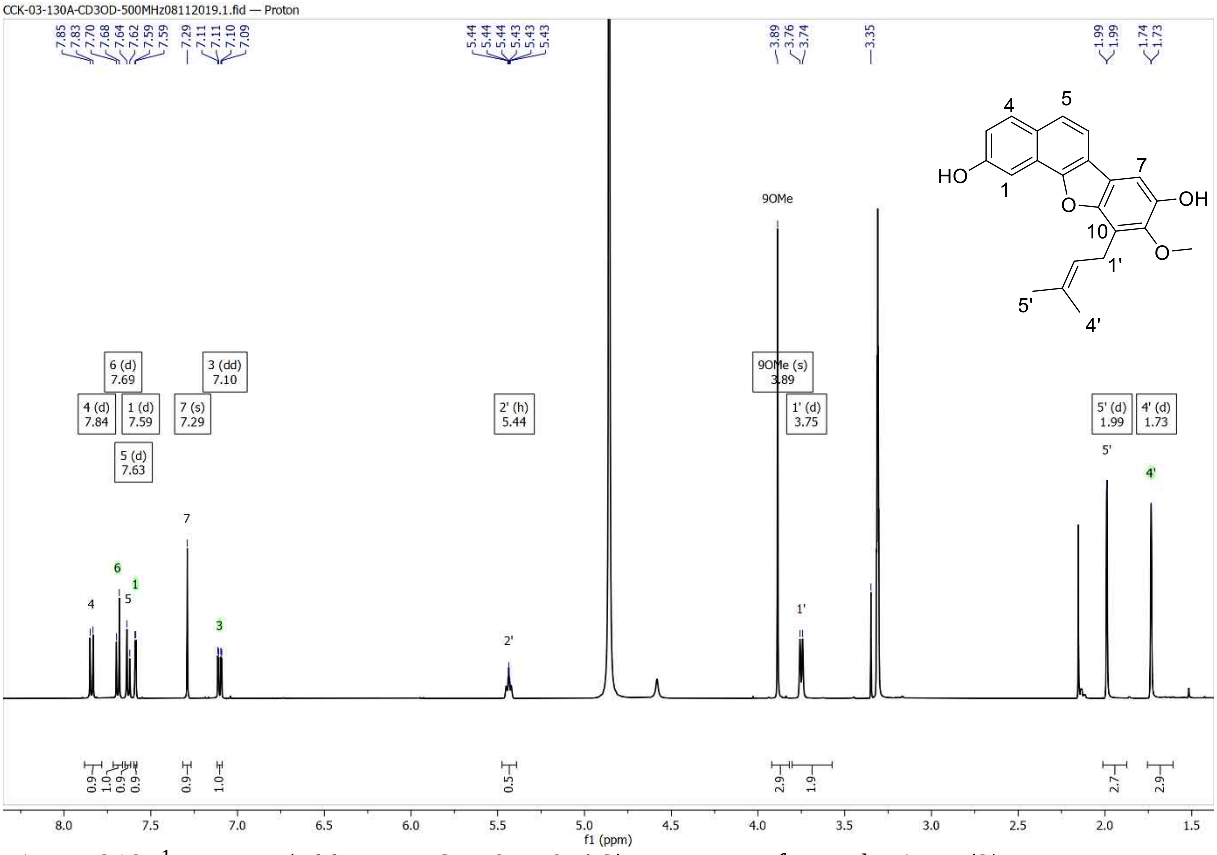

Figure S12. ^{1}H NMR (500 MHz, CD_3OD, 25°C) spectrum of usambarin B (**2**)

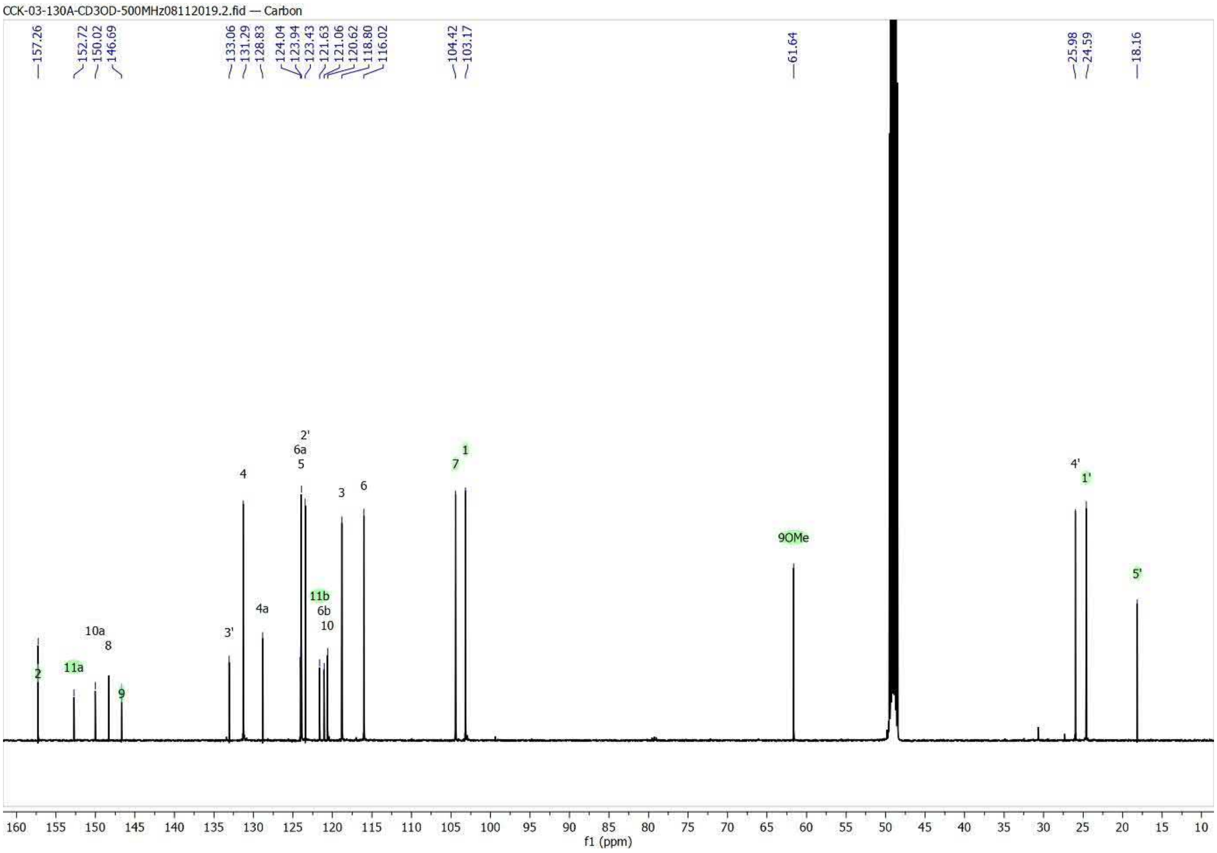

Figure S13. ^{13}C NMR (125 MHz, CD_3OD, 25°C) spectrum of usambarin B (**2**)

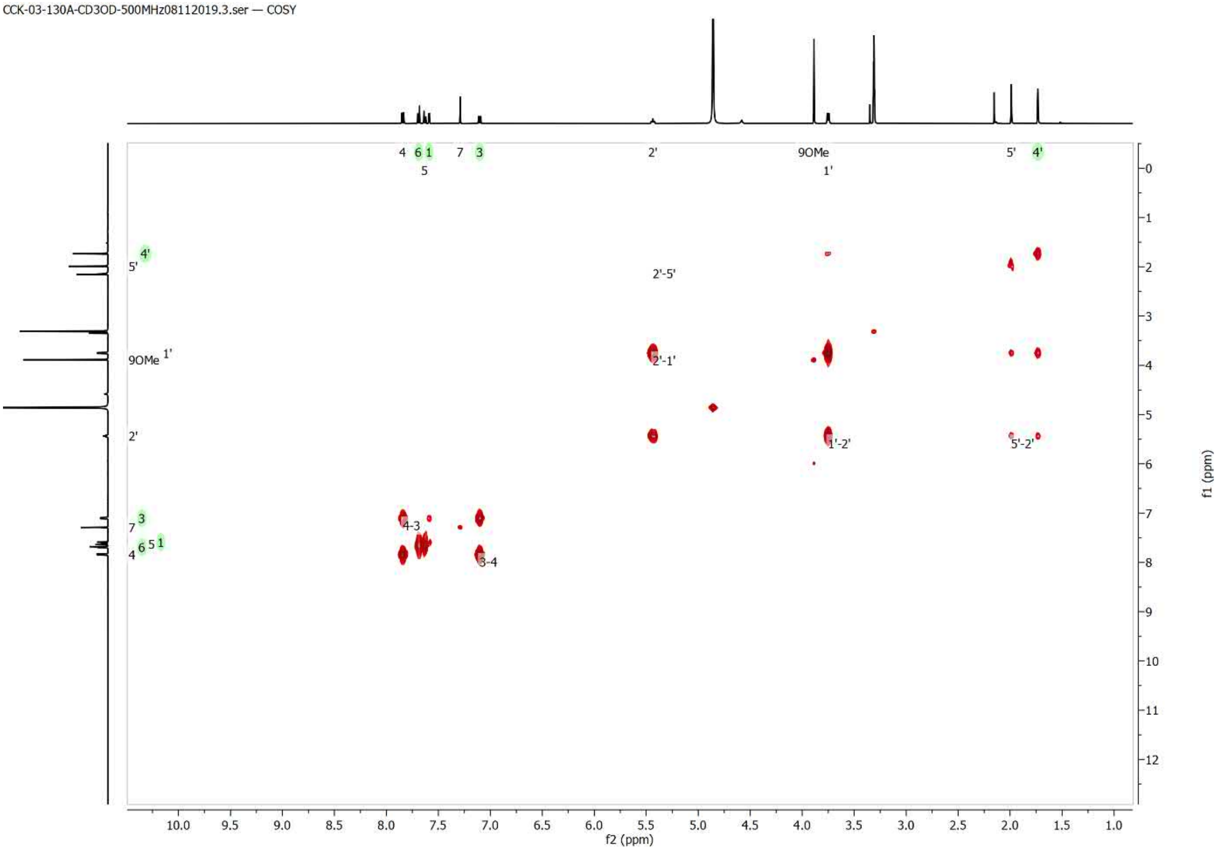

Figure S14.COSY (500 MHz, CD_3OD, 25°C) spectrum of usambarin B (**2**)

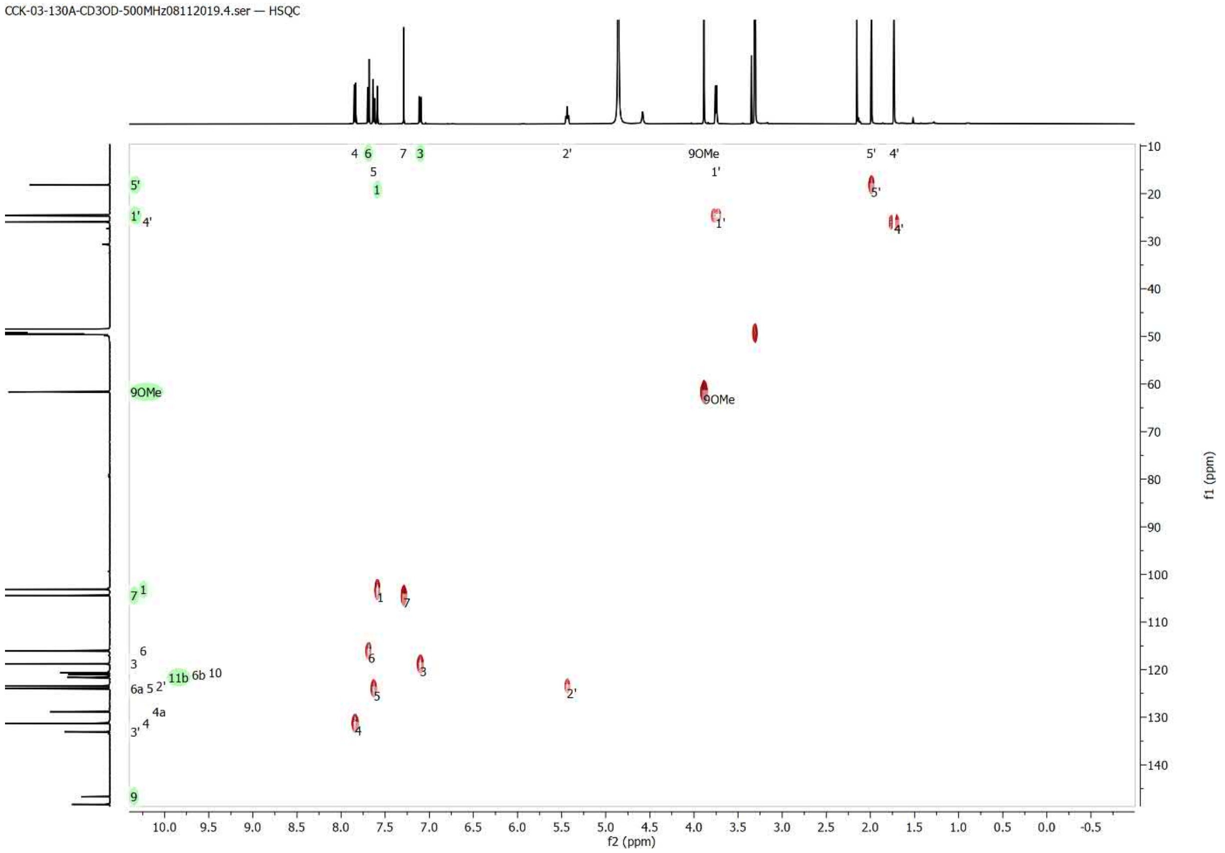

Figure S15. HSQC (500 MHz, CD_3OD, 25°C) spectrum of usambarin B (**2**)

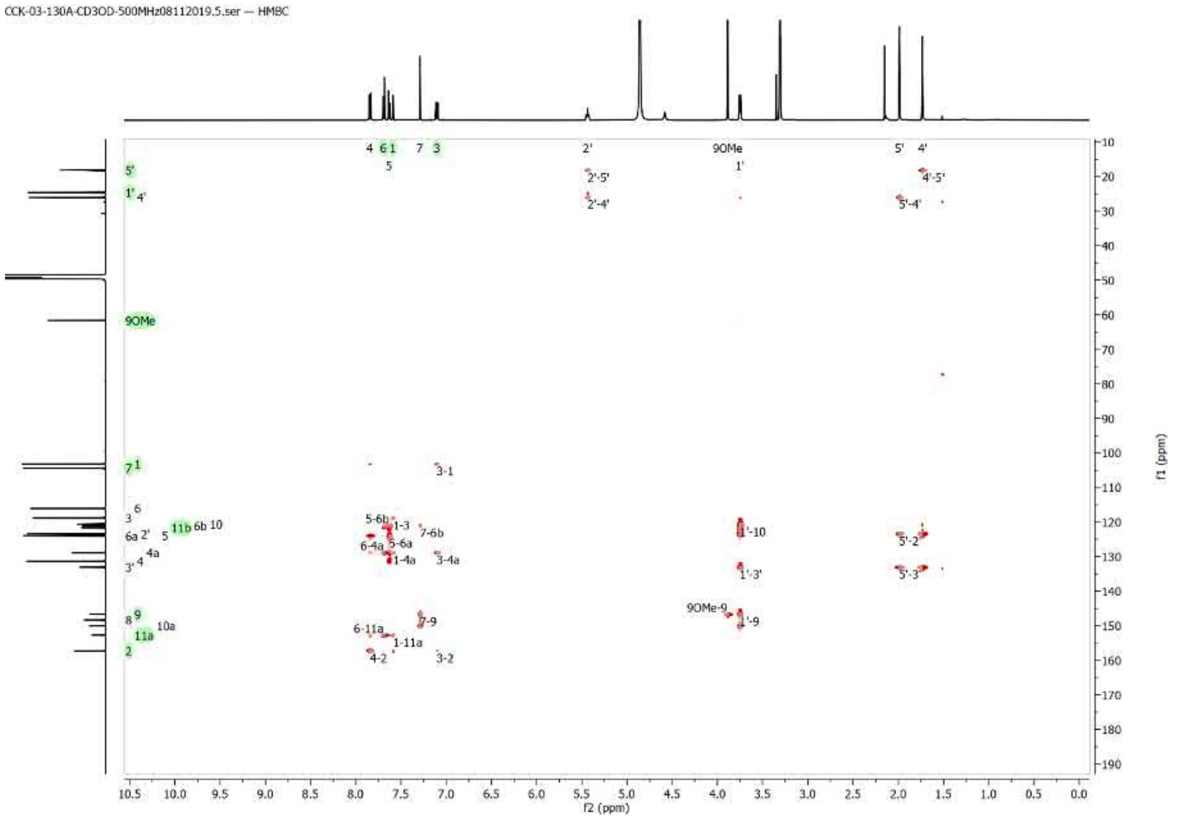

Figure S16. HMBC (500 MHz, CD_3OD, 25°C) spectrum of usambarin B (**2**)

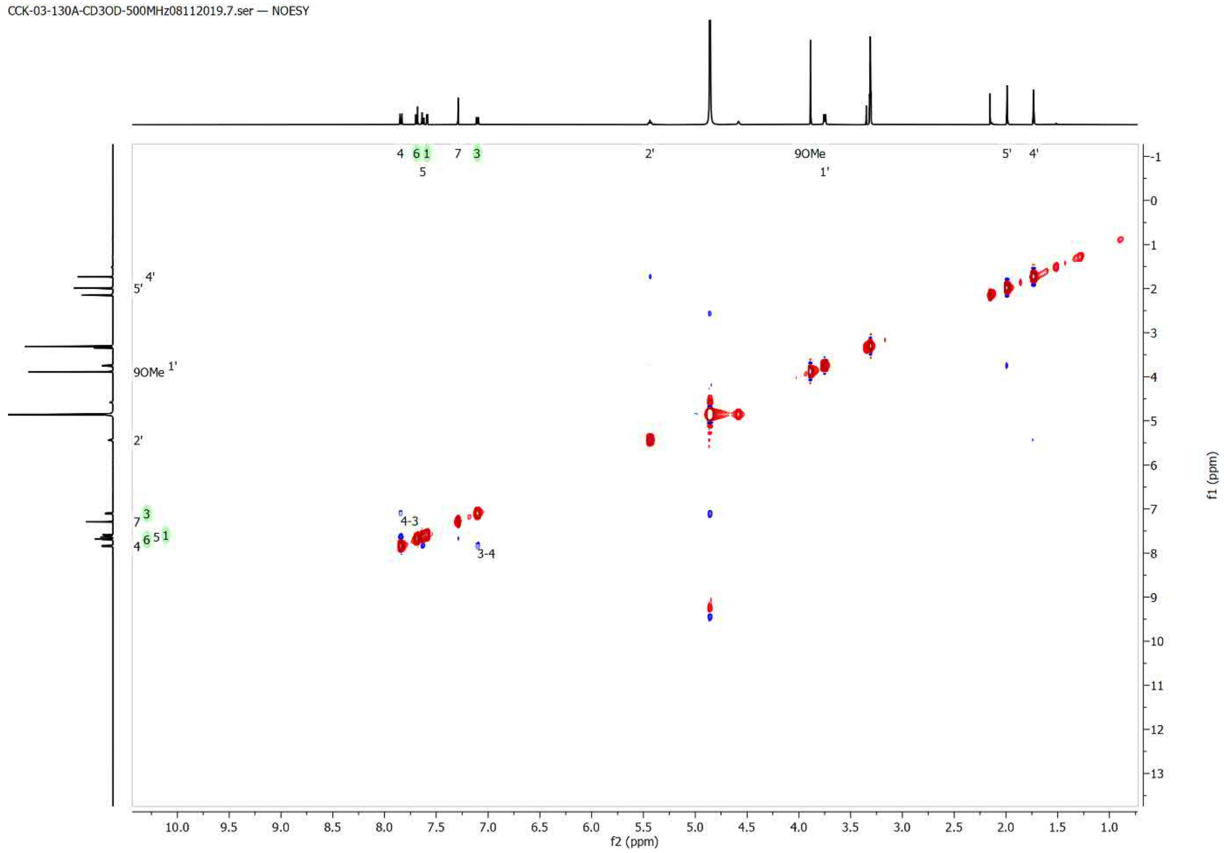

Figure S17. NOESY (500 MHz, CD_3OD, 25°C) spectrum of usambarin B (**2**)

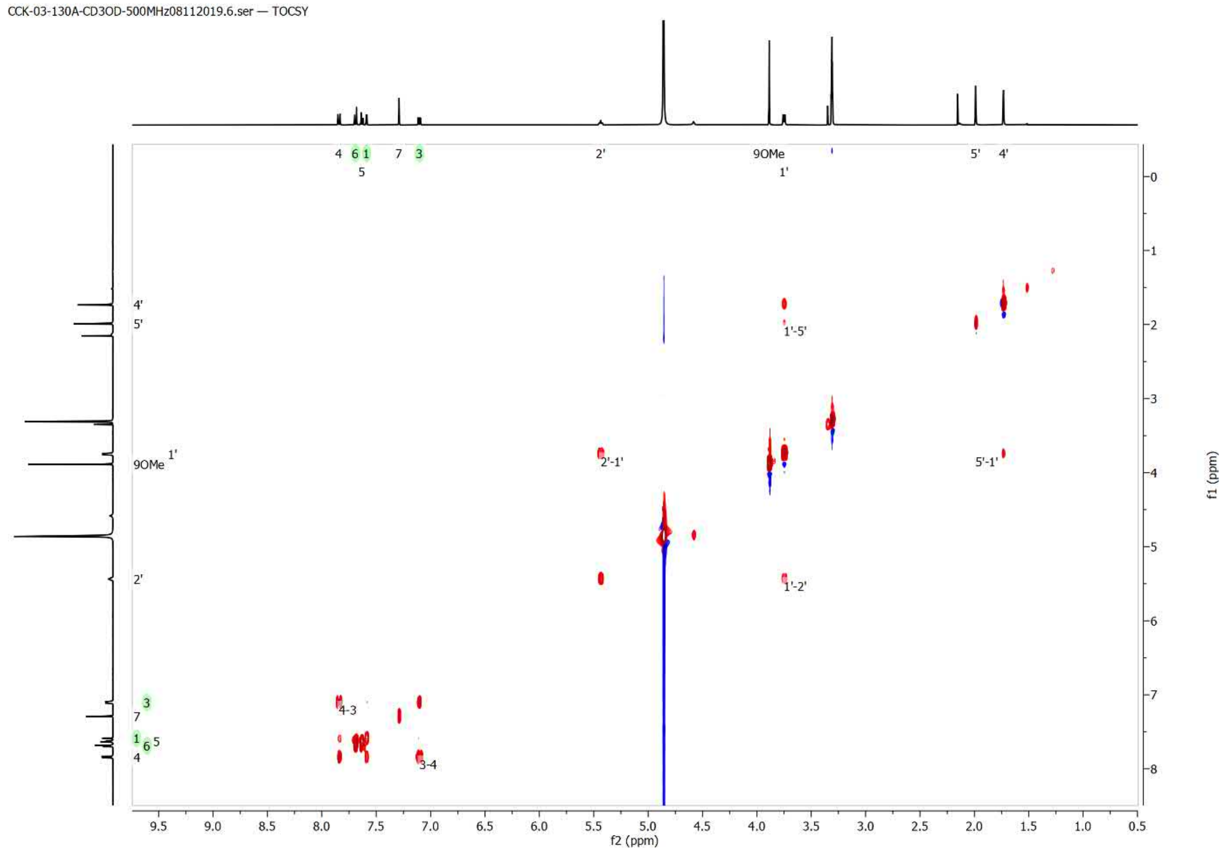

Figure S18.TOCSY (500 MHz, CD_3OD, 25°C) spectrum of usambarin B (**2**)

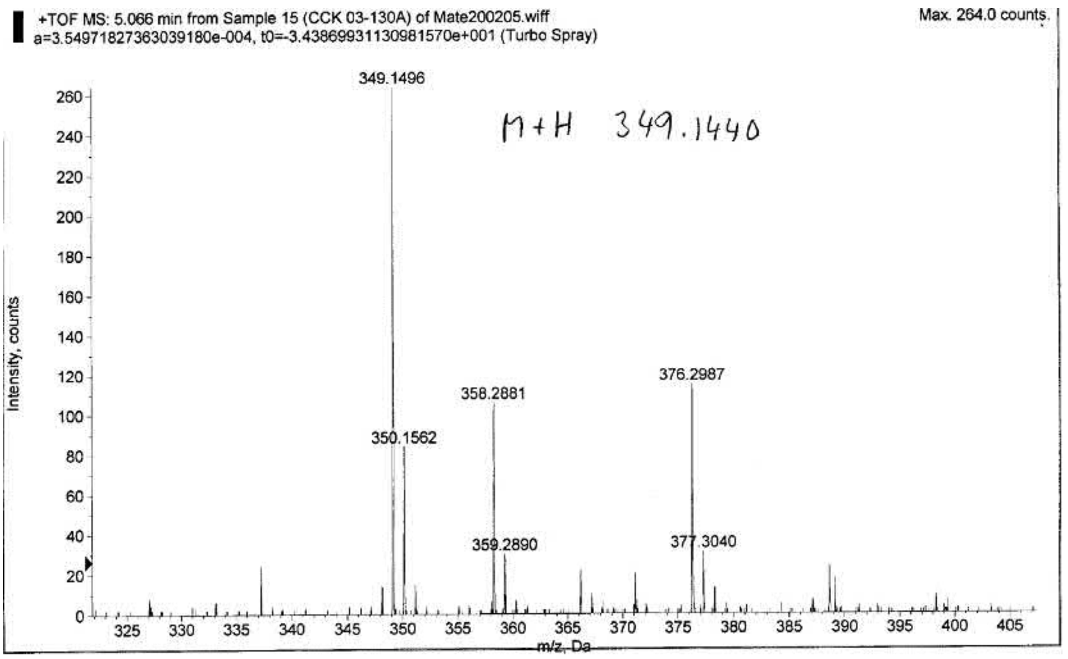

Figure S19 HRMS spectrum of usambarin B (**2**)

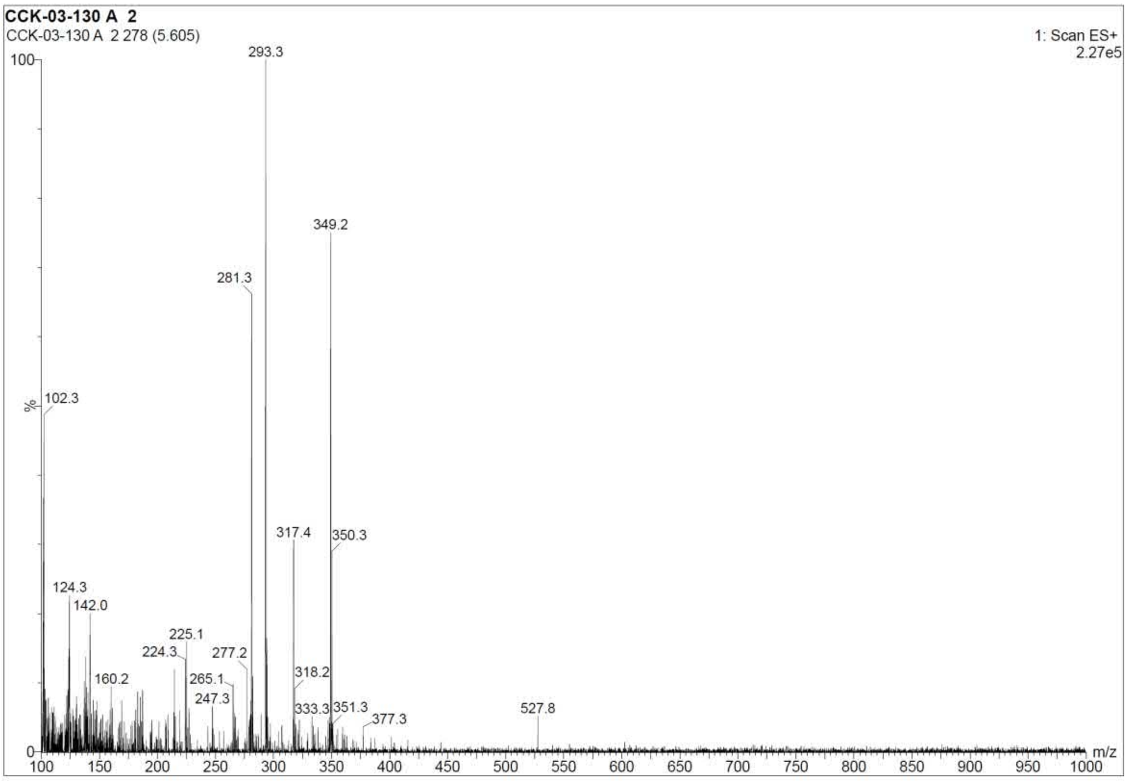

Figure S20. ESI-MS spectrum of usambarin B (**2**)

Spectroscopic data of Usambarin C (3)

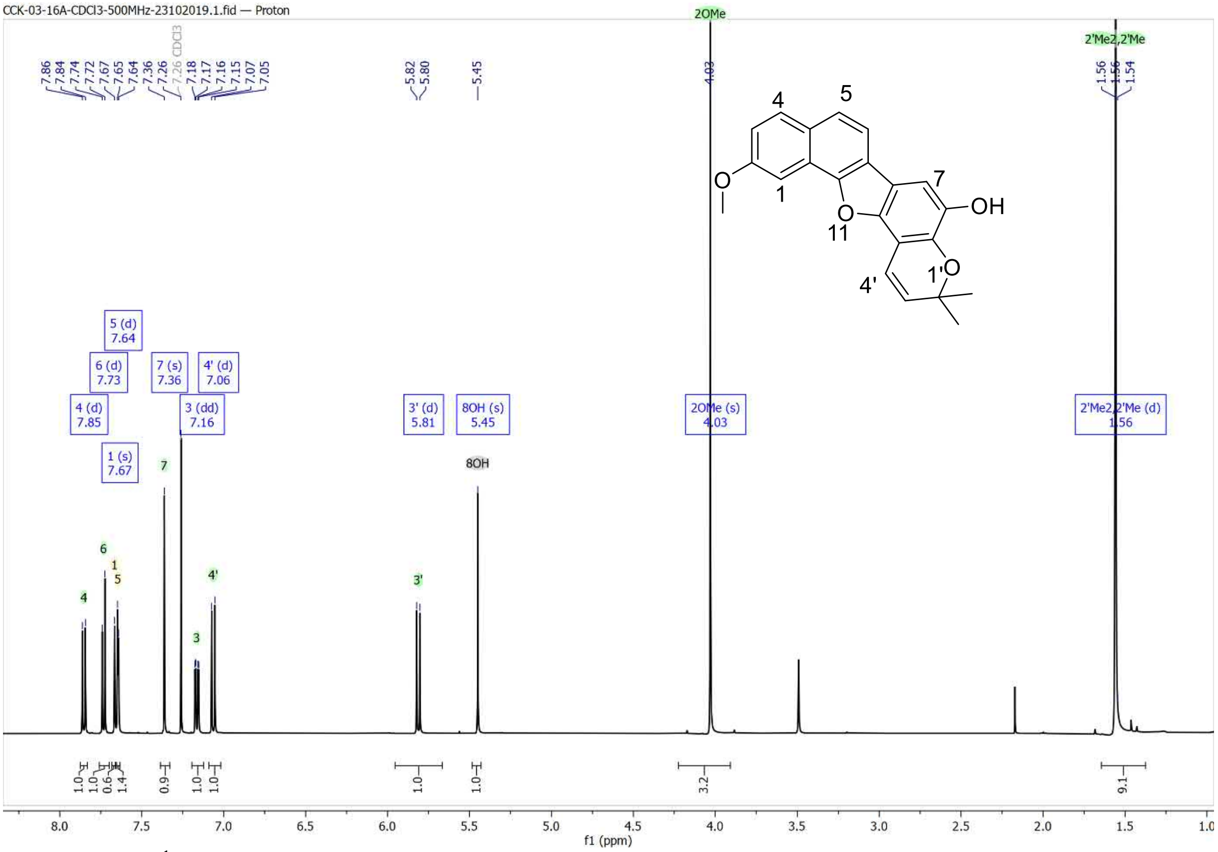

Figure S21. ^{1}H NMR (500 MHz, $CDCl_3$, 25°C) spectrum of usambarin C (**3**)

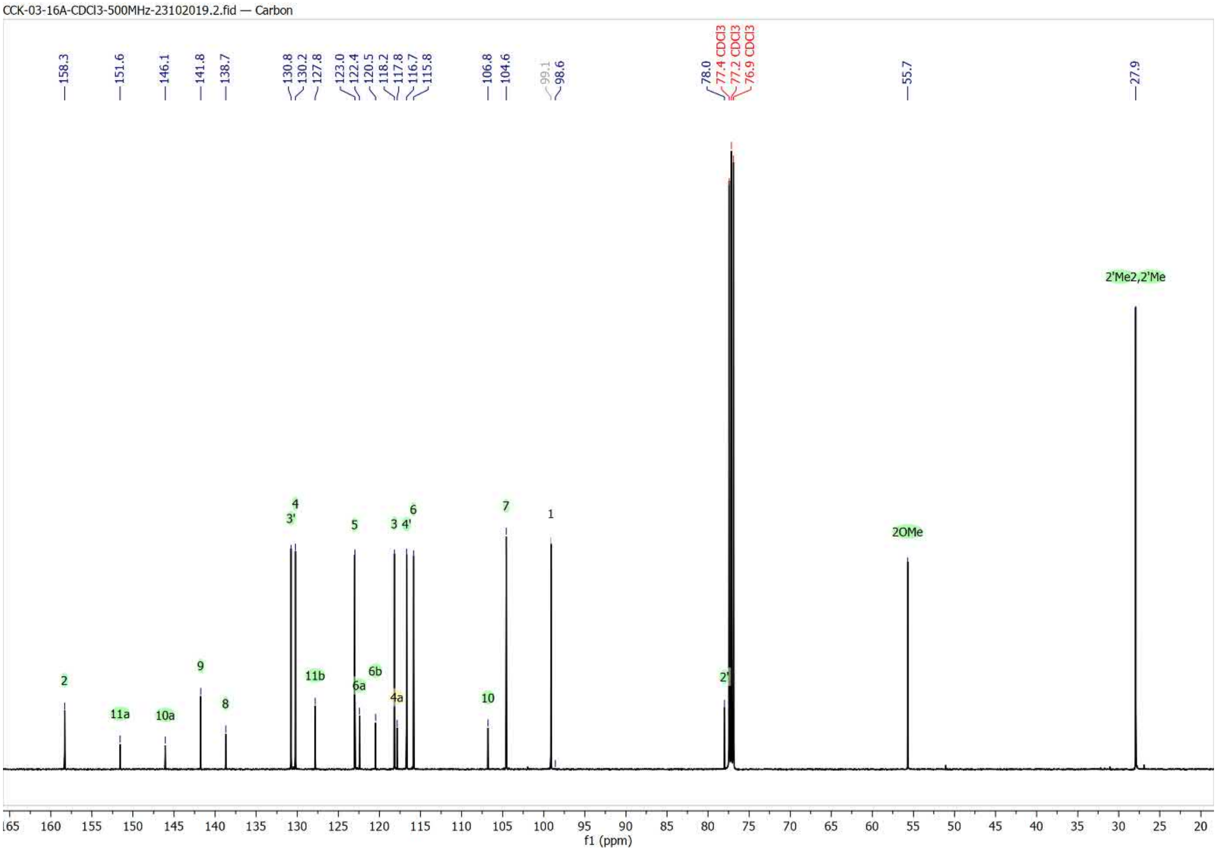

Figure S22. ^{13}C NMR (125 MHz, $CDCl_3$, 25°C) spectrum of usambarin C (**3**)

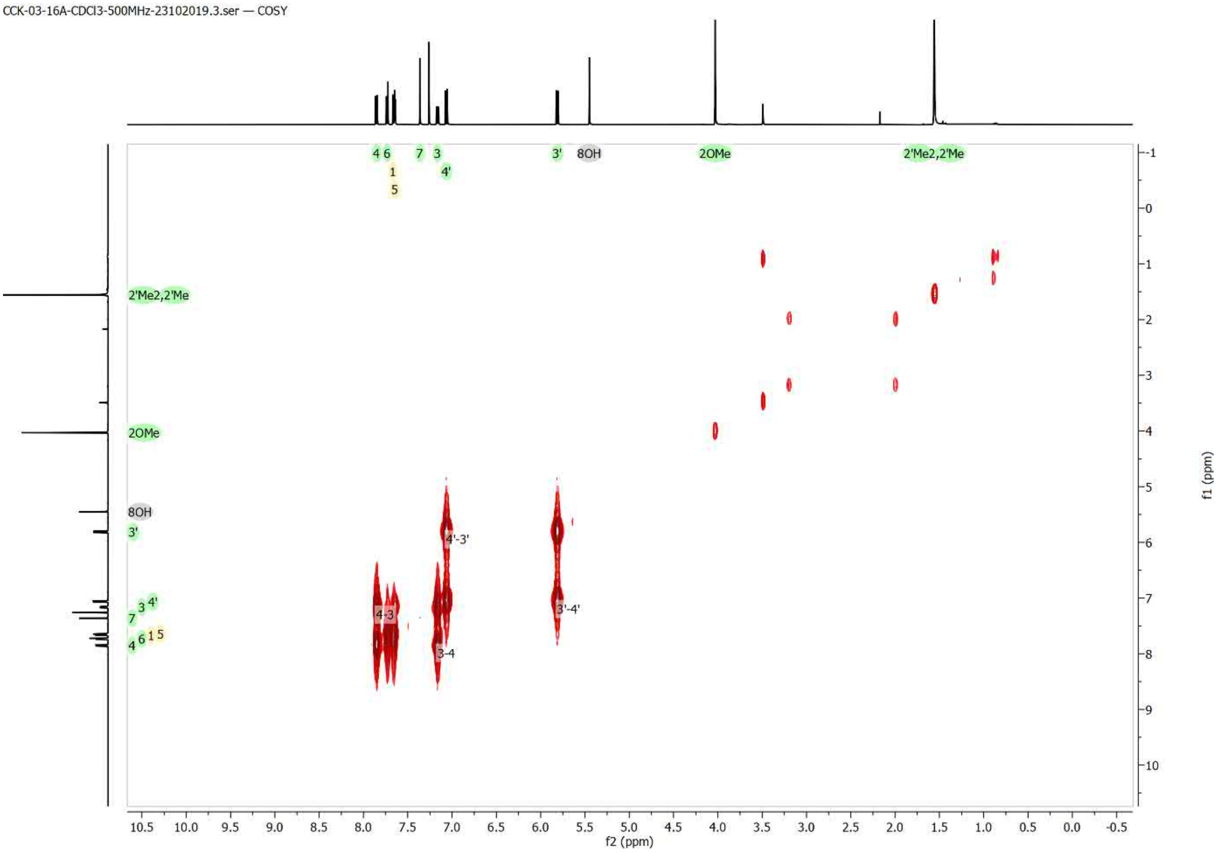

Figure S23. COSY (500 MHz, $CDCl_3$, 25°C) spectrum of usambarin C (**3**)

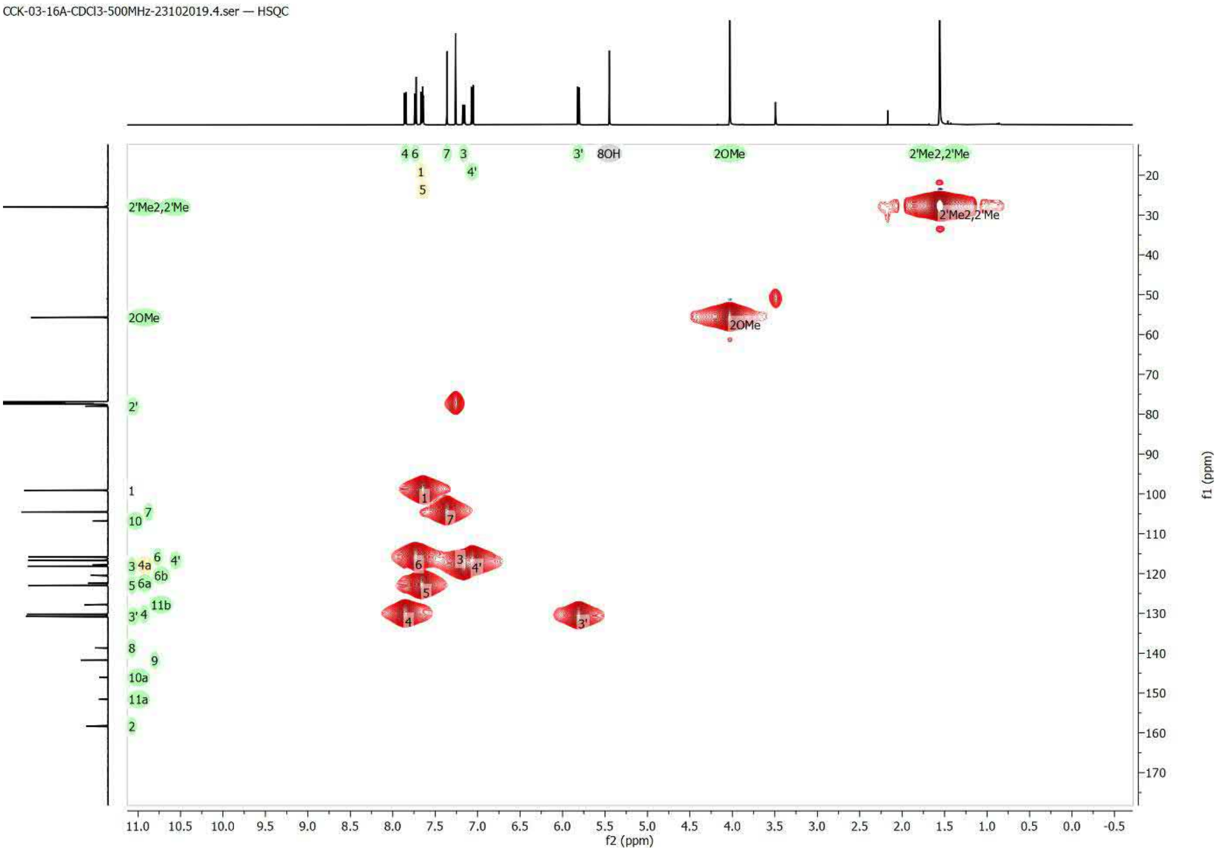

Figure S24. HSQC (500 MHz, $CDCl_3$, 25°C) spectrum of usambarin C (**3**)

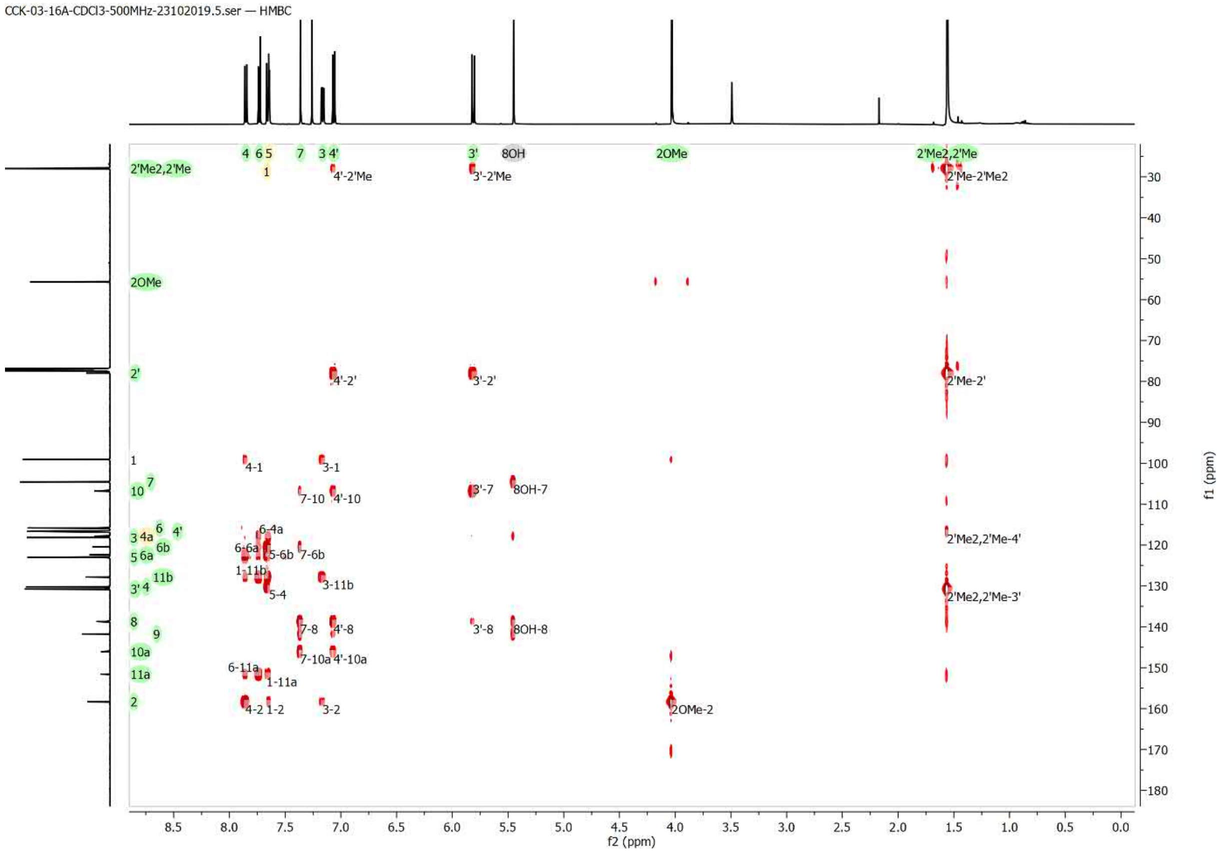

Figure S25. HMBC (500 MHz, $CDCl_3$, 25°C) spectrum of usambarin C (**3**)

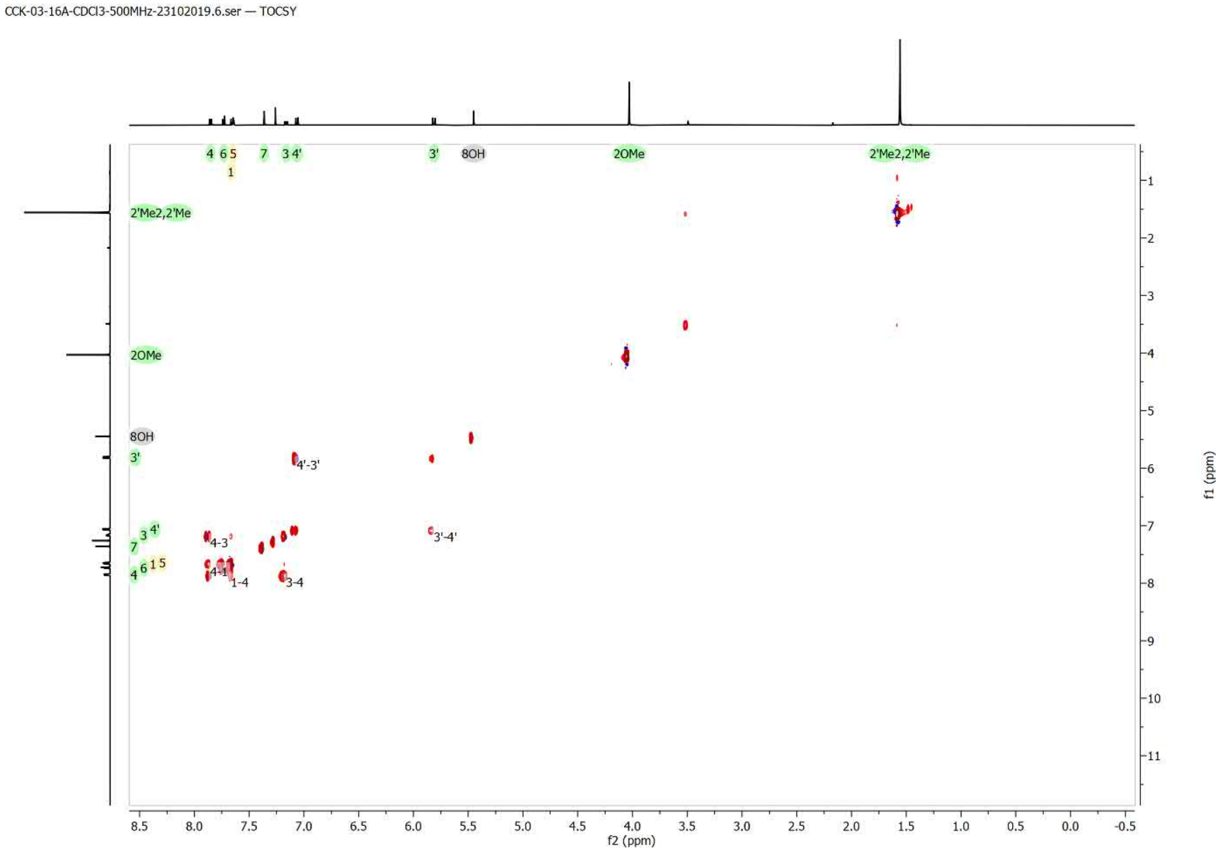

Figure S26**.**TOCSY (500 MHz, $CDCl_3$, 25°C) spectrum of usambarin C (**3**)

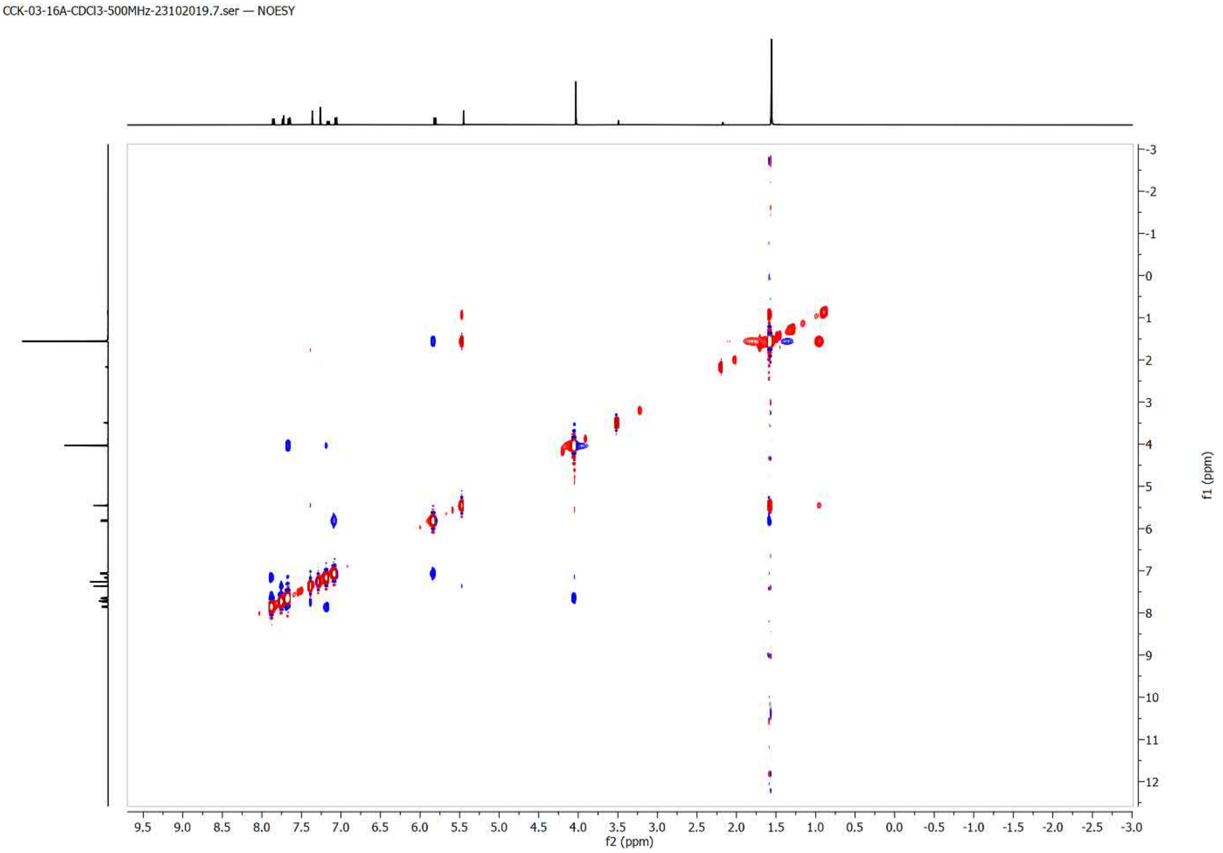

Figure S27 .NOESY (500 MHz, $CDCl_3$, 25°C) spectrum of usambarin C (**3**)

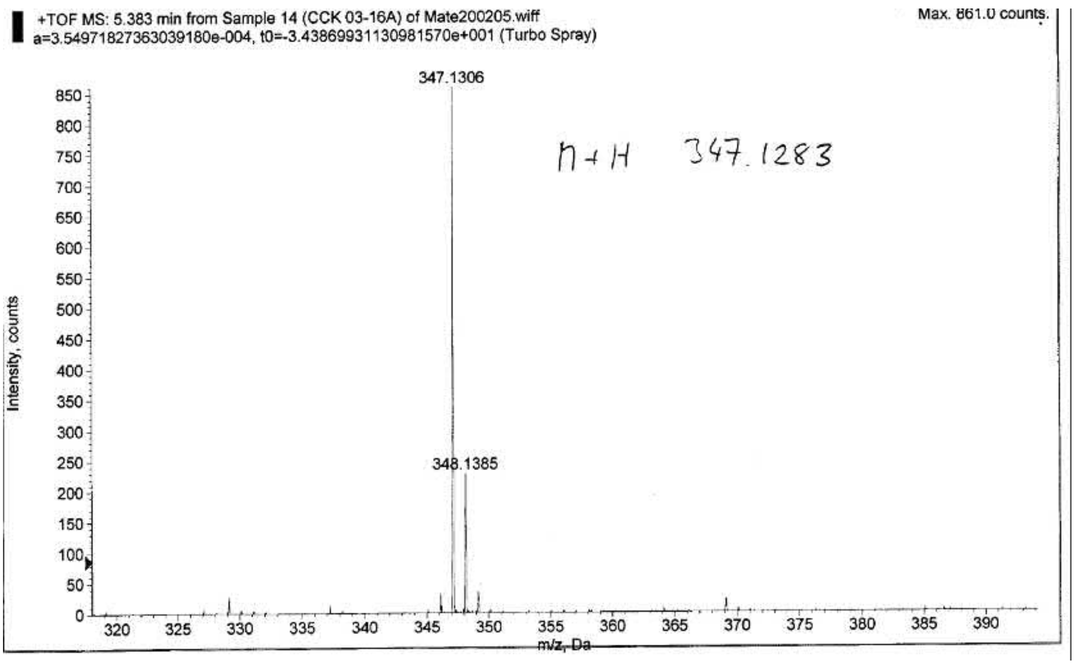

Figure S28. HRMS spectrum of usambarin C (**3**)

Spectroscopic data of usambarin D (4)

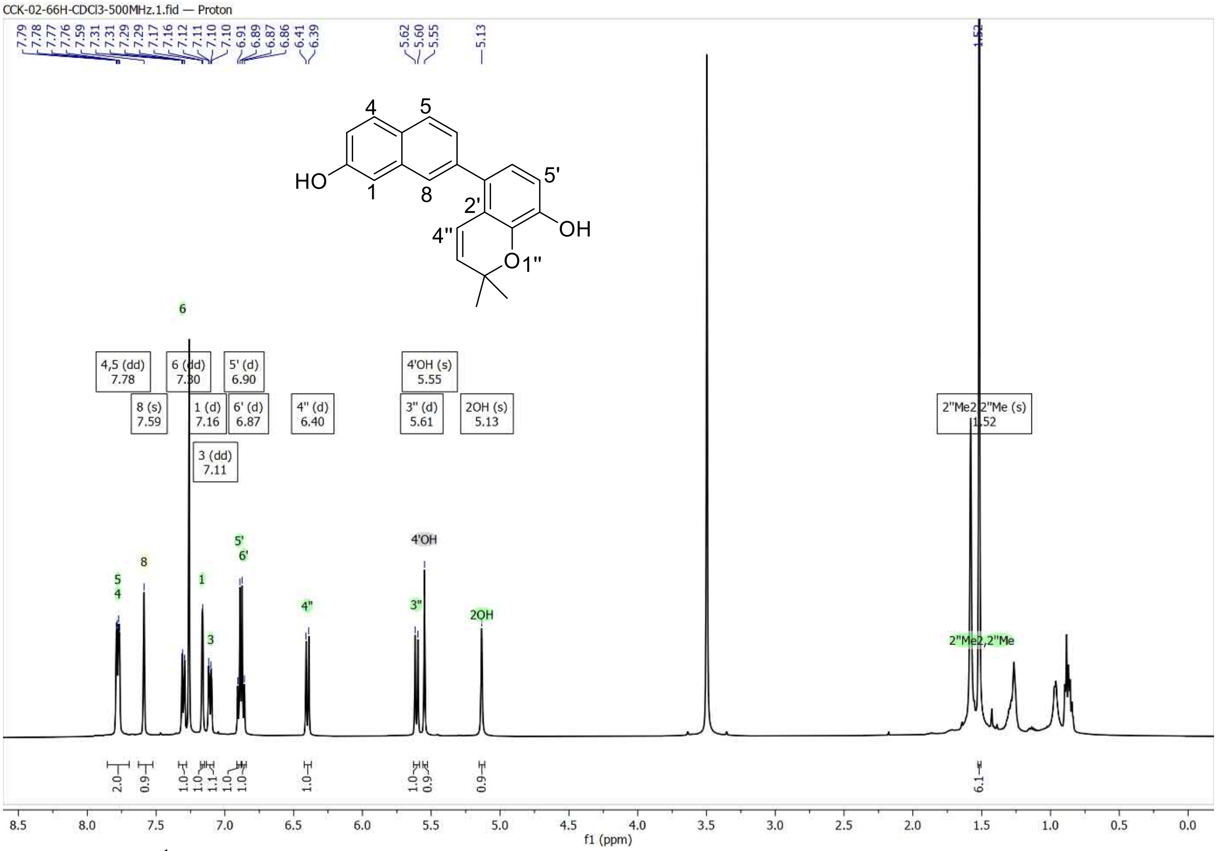

Figure S29. ^{1}H NMR (500 MHz, $CDCl_3$, 25°C) spectrum of usambarin D (**4**)

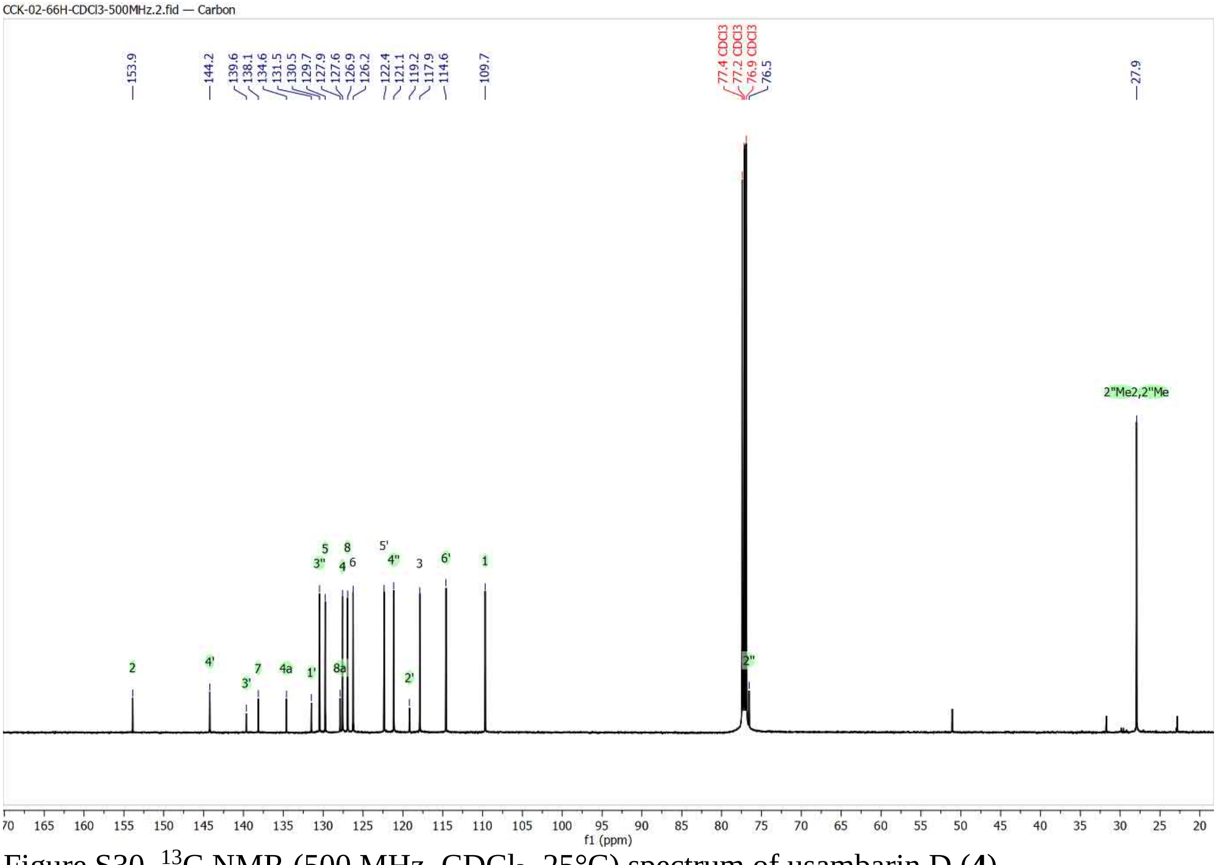

Figure S30. ^{13}C NMR (500 MHz, $CDCl_3$, 25°C) spectrum of usambarin D (**4**)

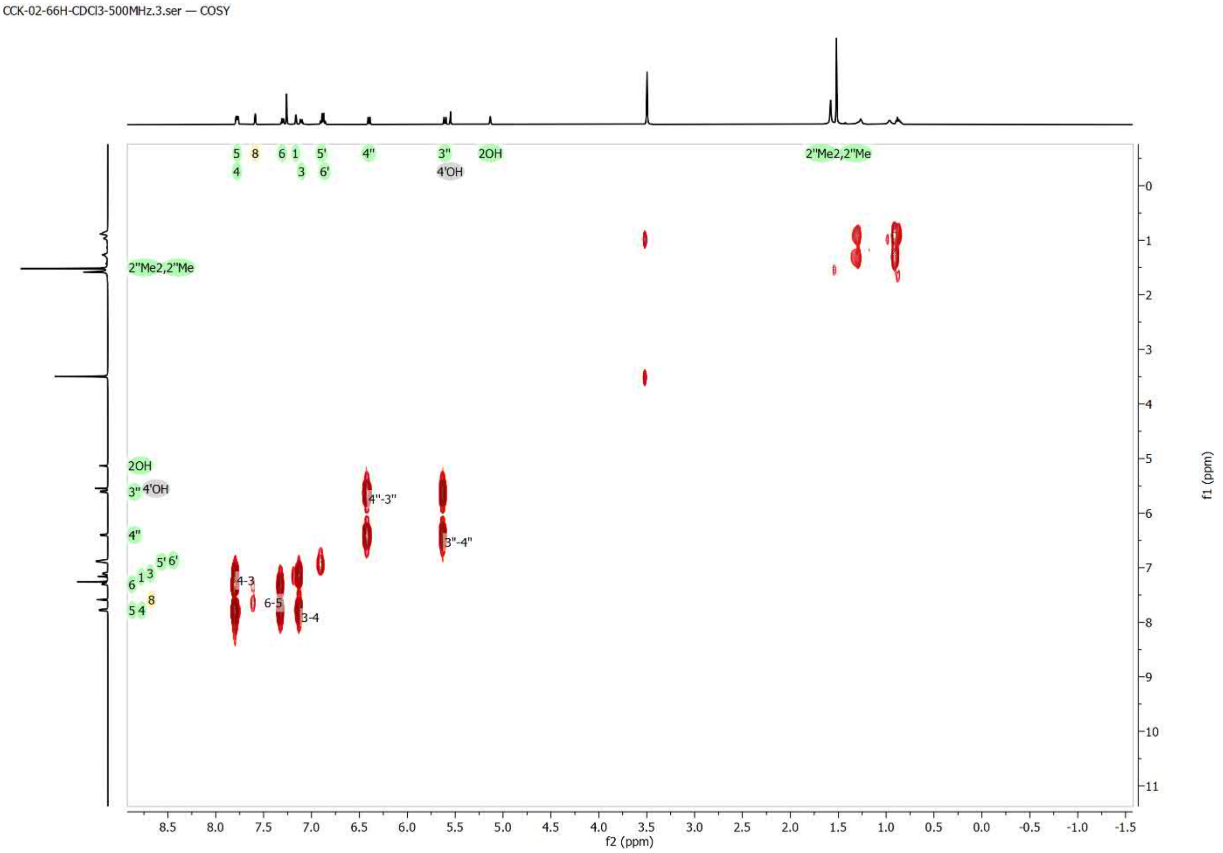

Figure S31. COSY (500 MHz, $CDCl_3$, 25°C) spectrum of usambarin D (**4**)

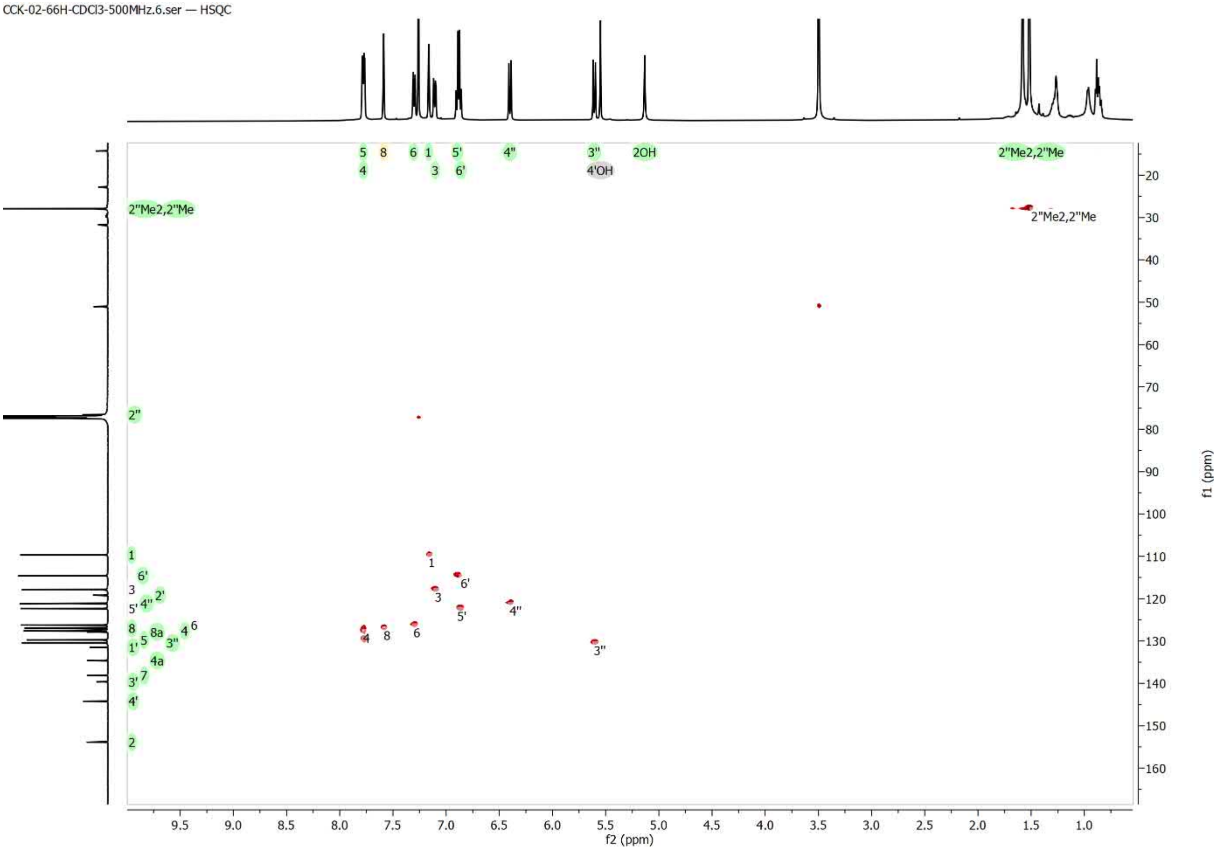

Figure S32. HSQC (500 MHz, $CDCl_3$, 25°C) spectrum of usambarin D (**4**)

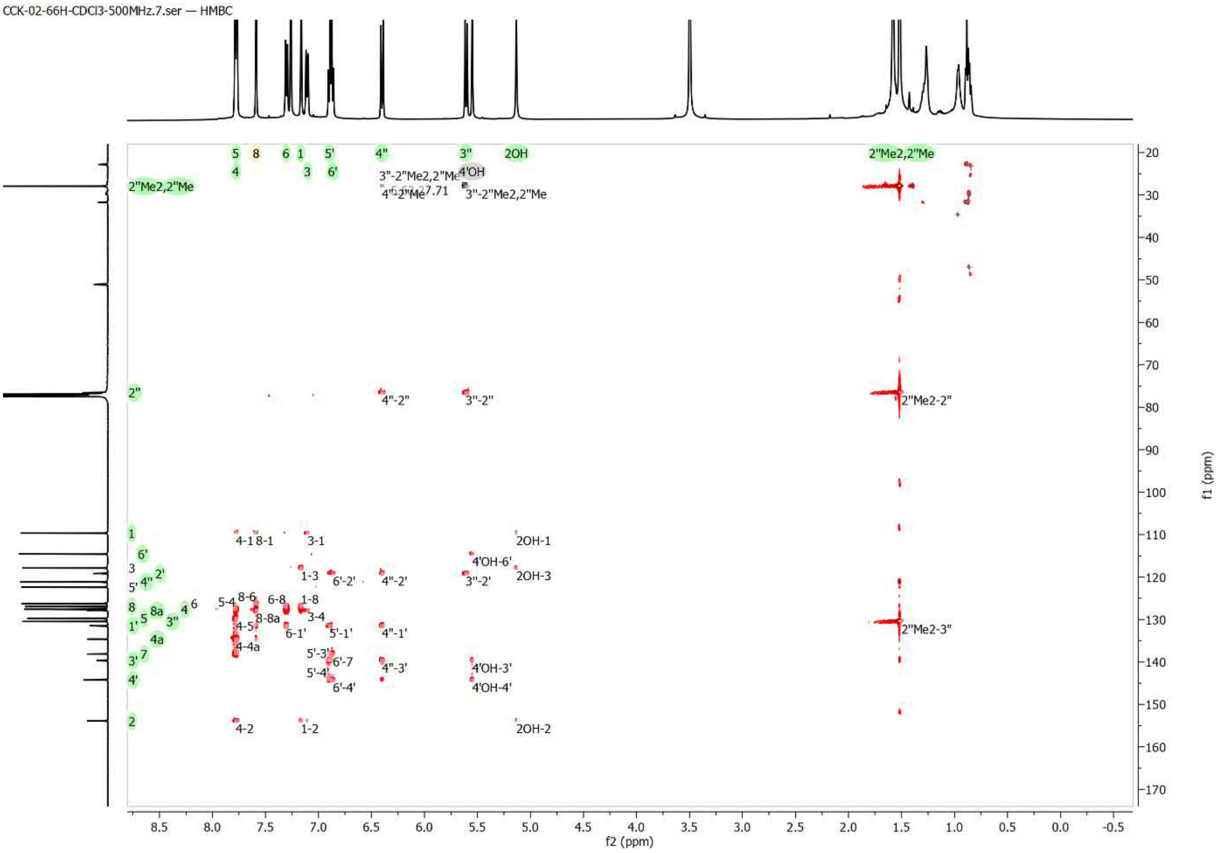

Figure S33. HMBC (500 MHz, $CDCl_3$, 25°C) spectrum of usambarin D (**4**)

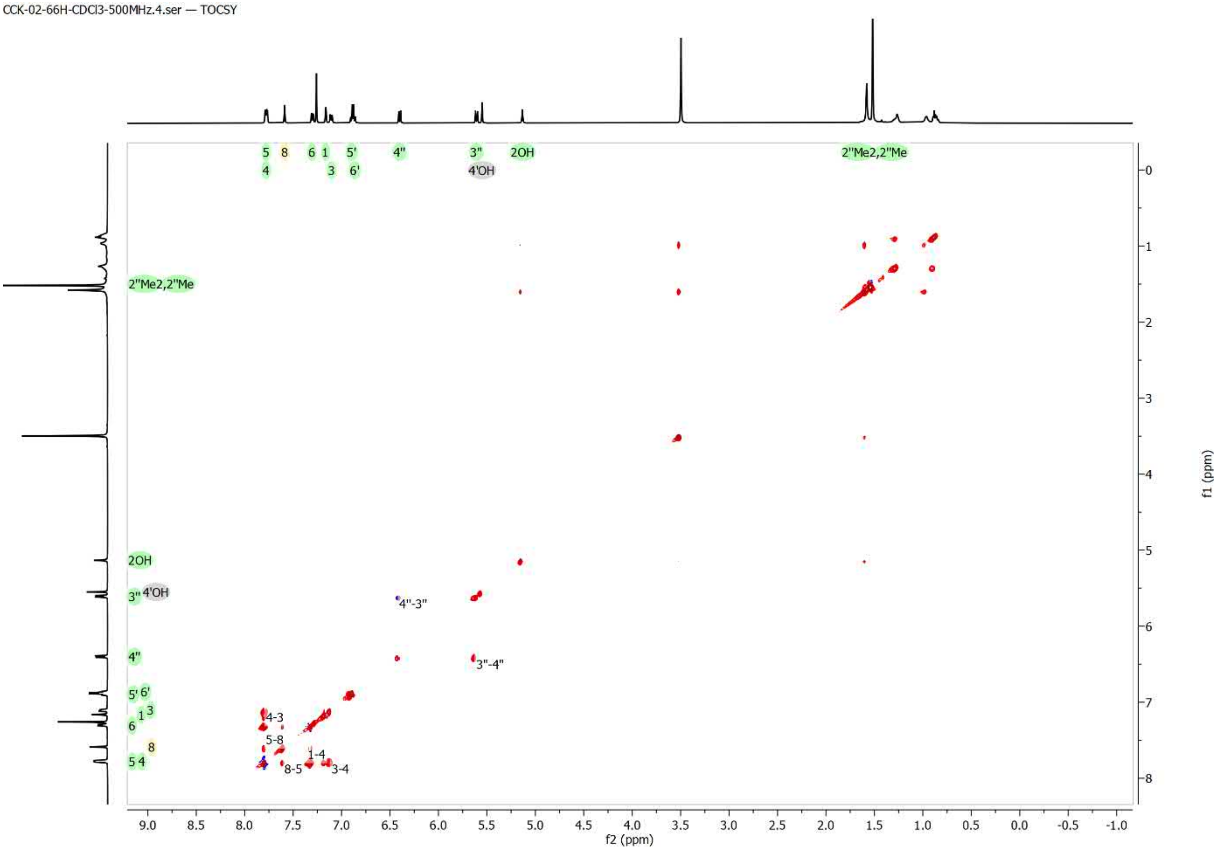

Figure S34. TOCSY (500 MHz, $CDCl_3$, 25°C) spectrum of usambarin D (**4**)

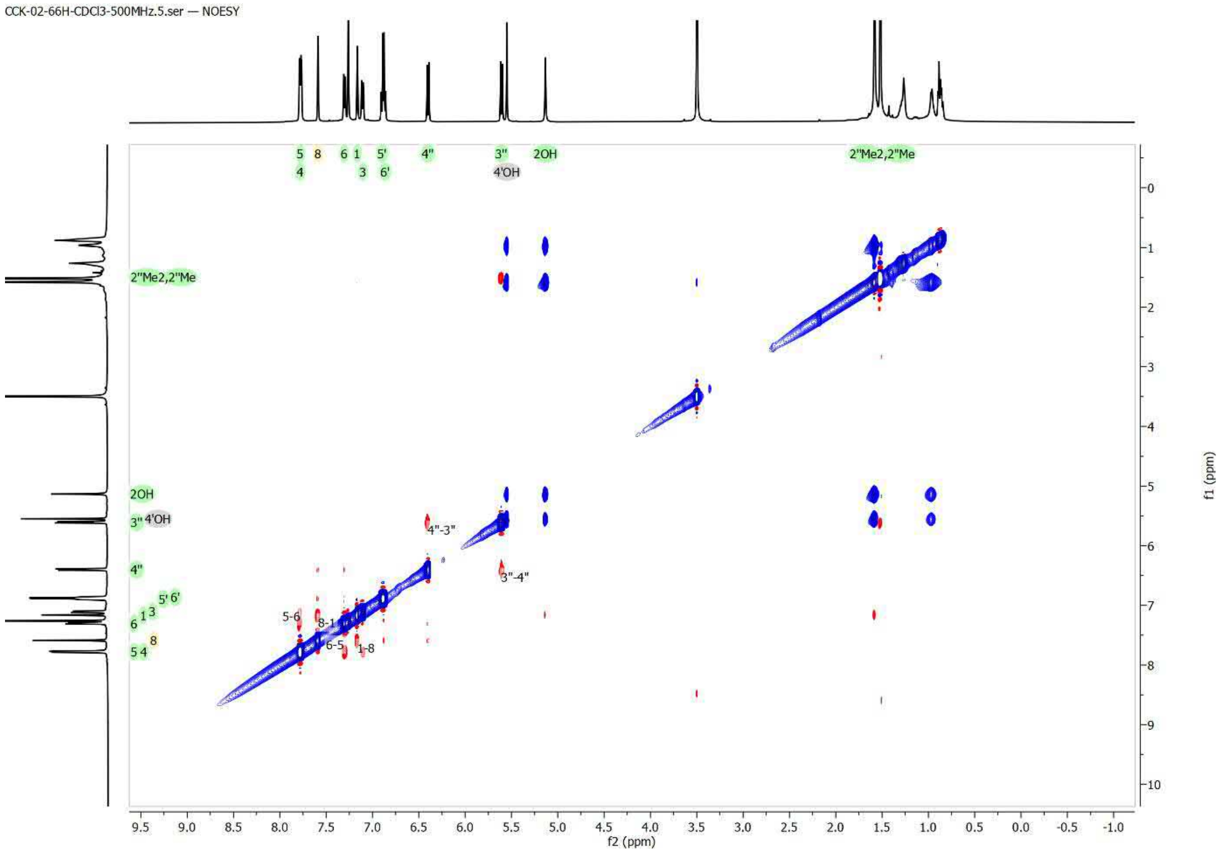

Figure S35. NOESY (500 MHz, $CDCl_3$, 25°C) spectrum of usambarin D (**4**)

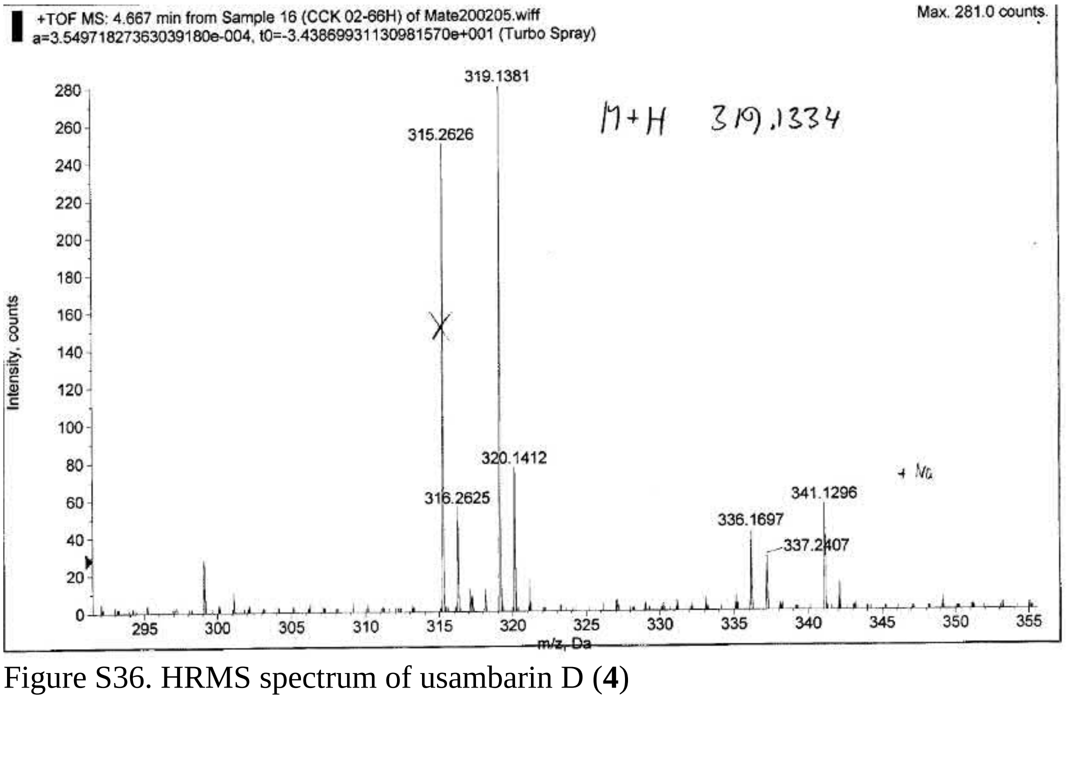

Figure S36. HRMS spectrum of usambarin D (**4**)

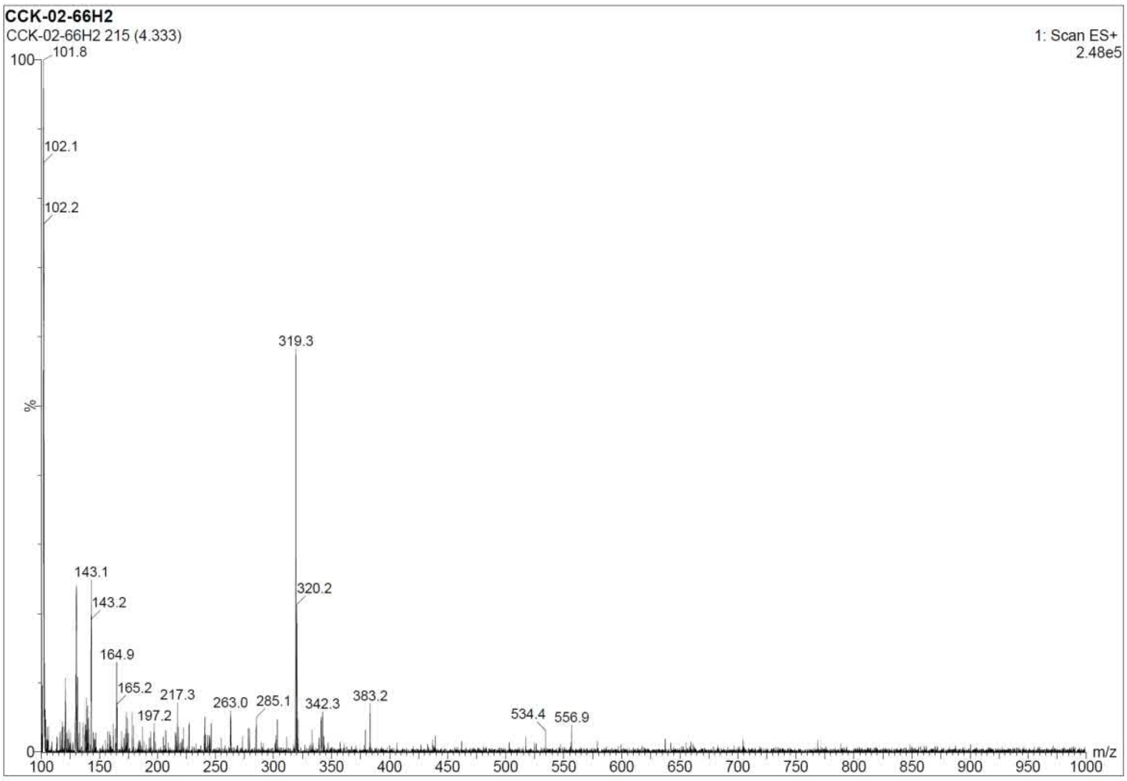

Figure S37. ESI-MS spectrum of usambarin D (**4**)

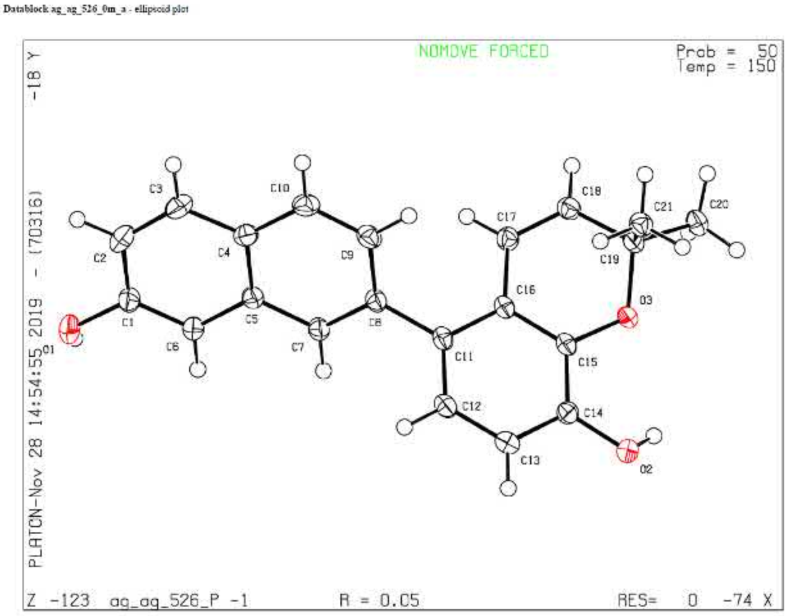

Figure S38. X-ray structure of usambarin D (**4)**

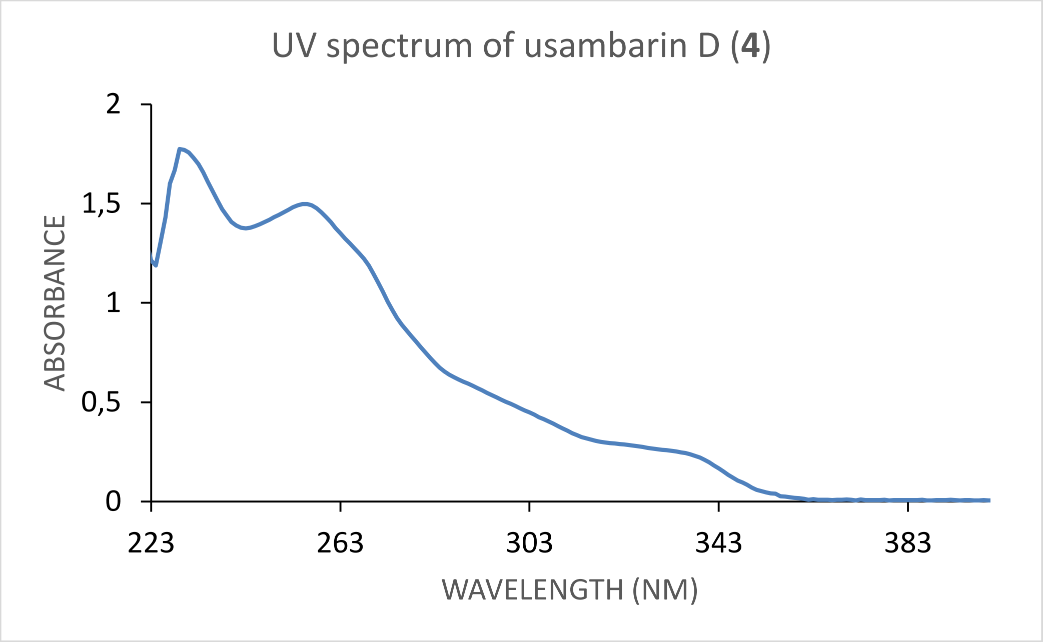

Figure S39. UV spectrum (MeOH) of usambarin D (**4**)

Spectroscopic data of Usambarin E (5)

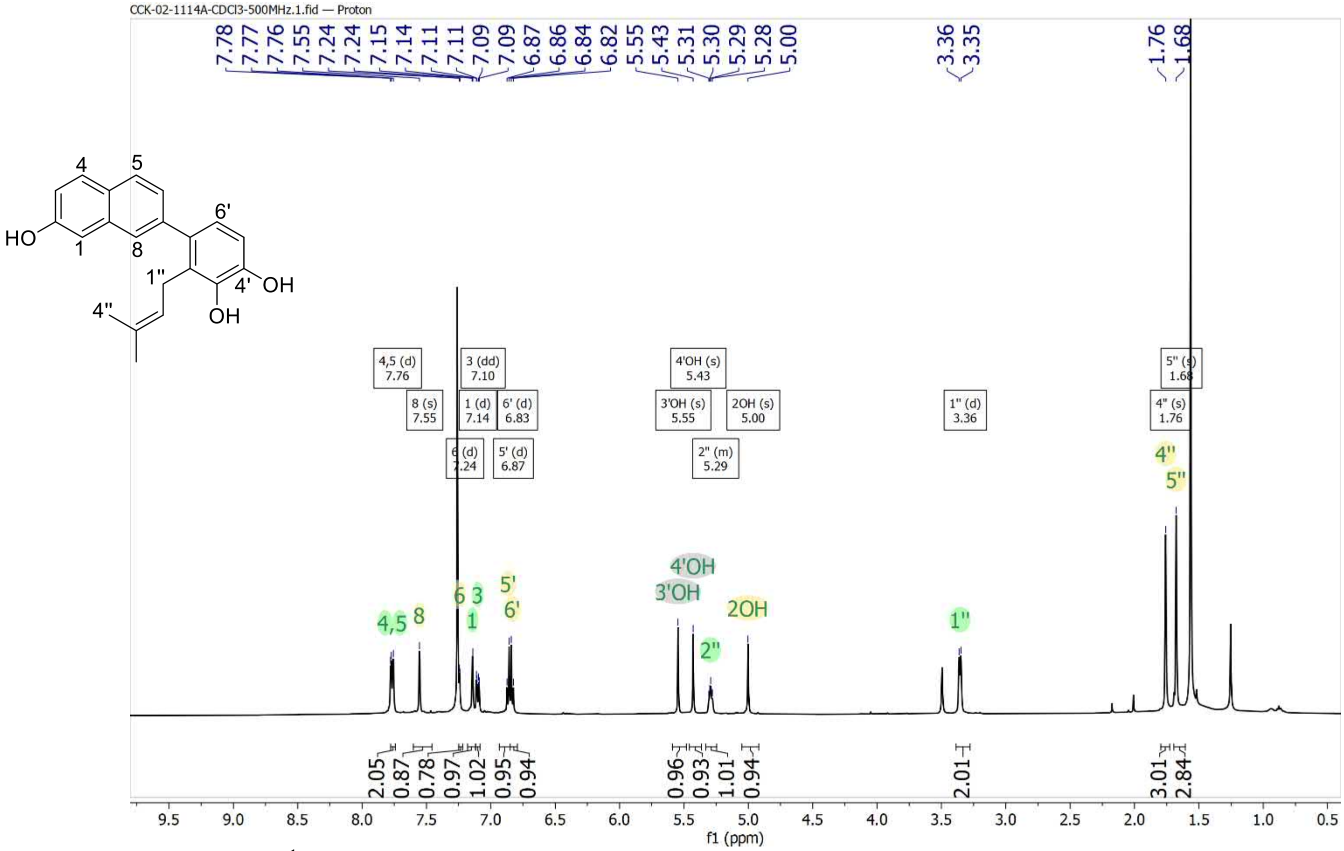

Figure S40. ^{1}H NMR (500 MHz, $CDCl_3$, 25°C) spectrum of usambarin E (**5**)

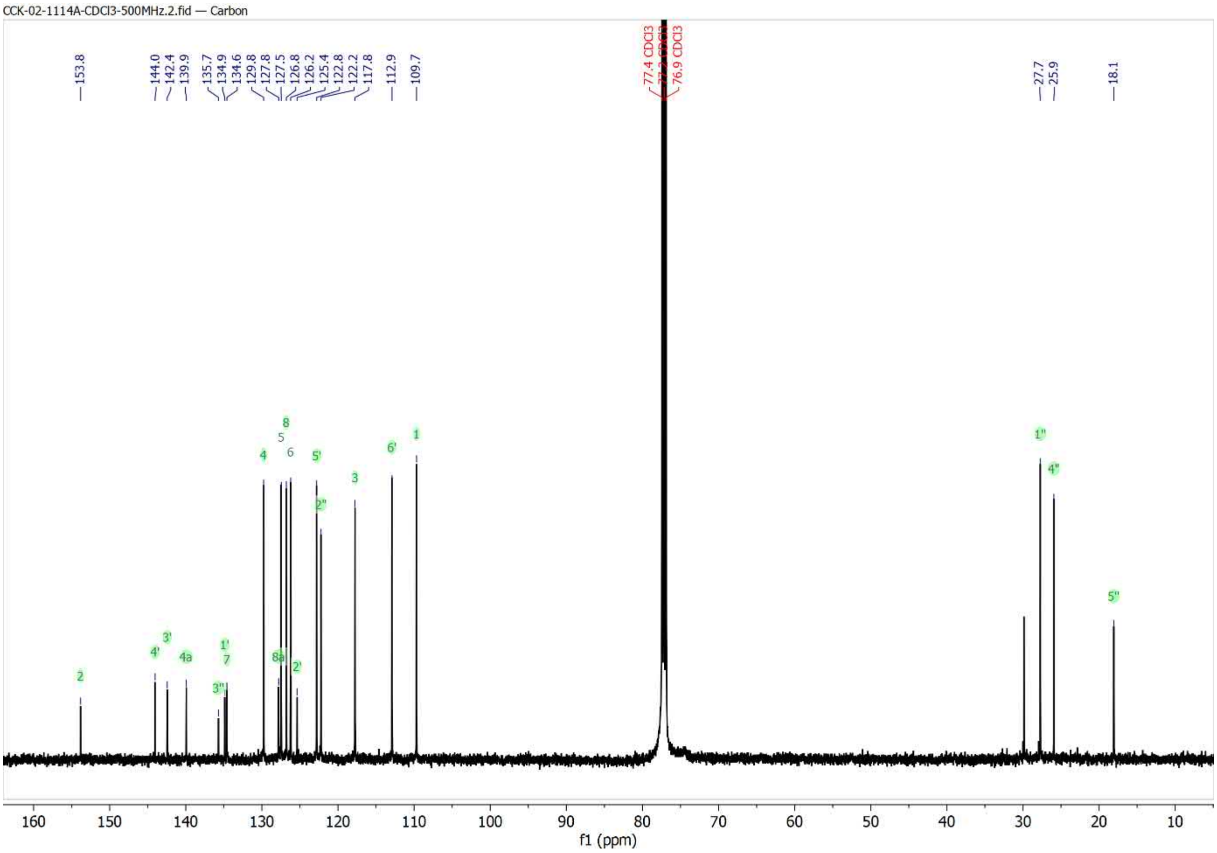

Figure S41. ^{13}C NMR (500 MHz, $CDCl_3$, 25°C) spectrum of usambarin E (**5**)

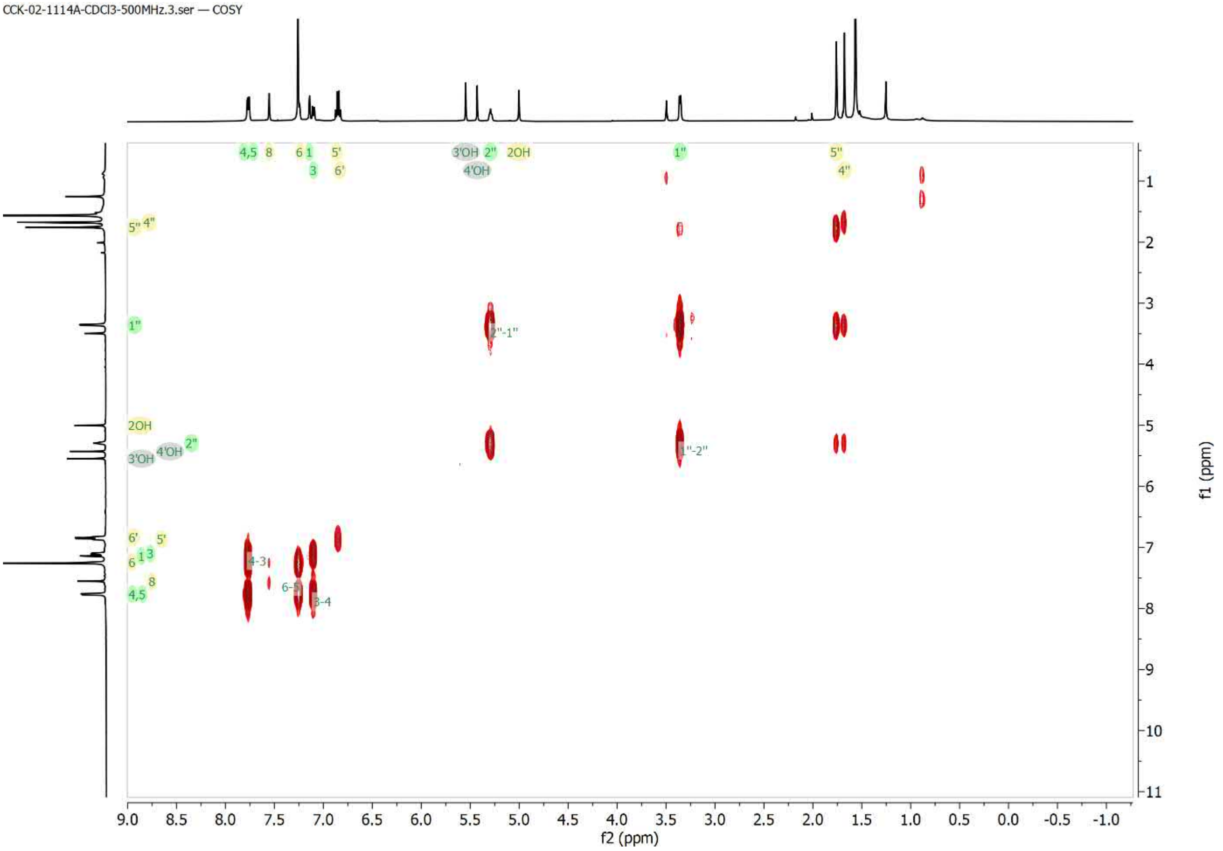

Figure S42. COSY (500 MHz, $CDCl_3$, 25°C) spectrum of usambarin E (**5**)

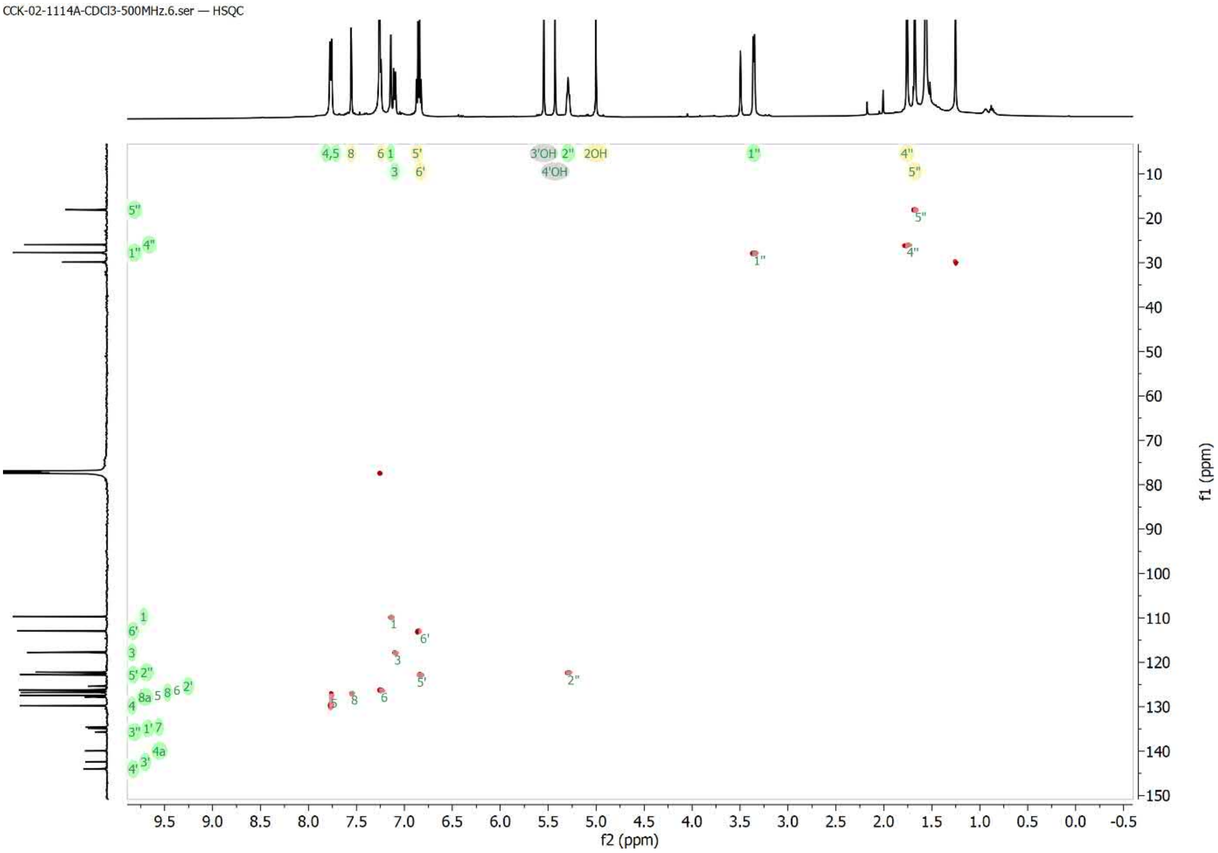

Figure S43. HSQC (500 MHz, $CDCl_3$, 25°C) spectrum of usambarin E (**5**)

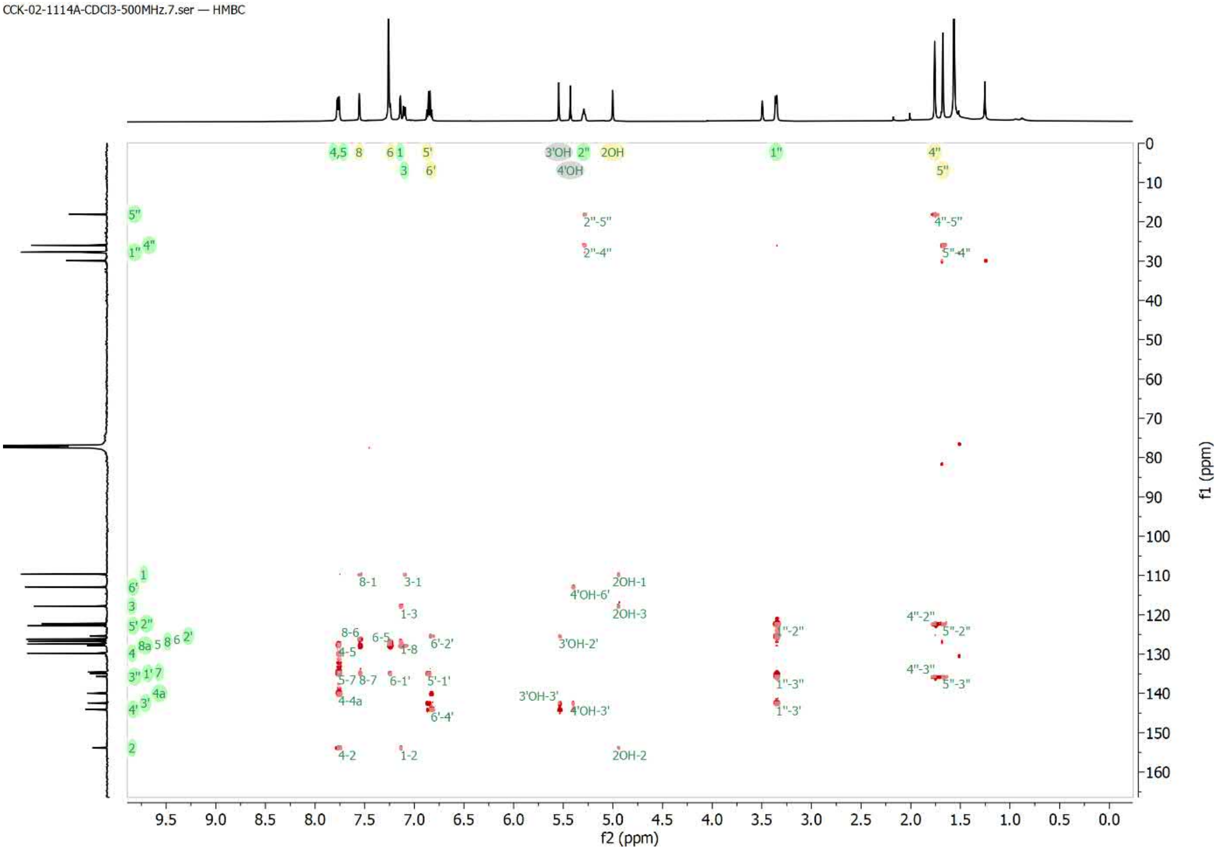

Figure S44. HMBC (500 MHz, $CDCl_3$, 25°C) spectrum of usambarin E (**5**)

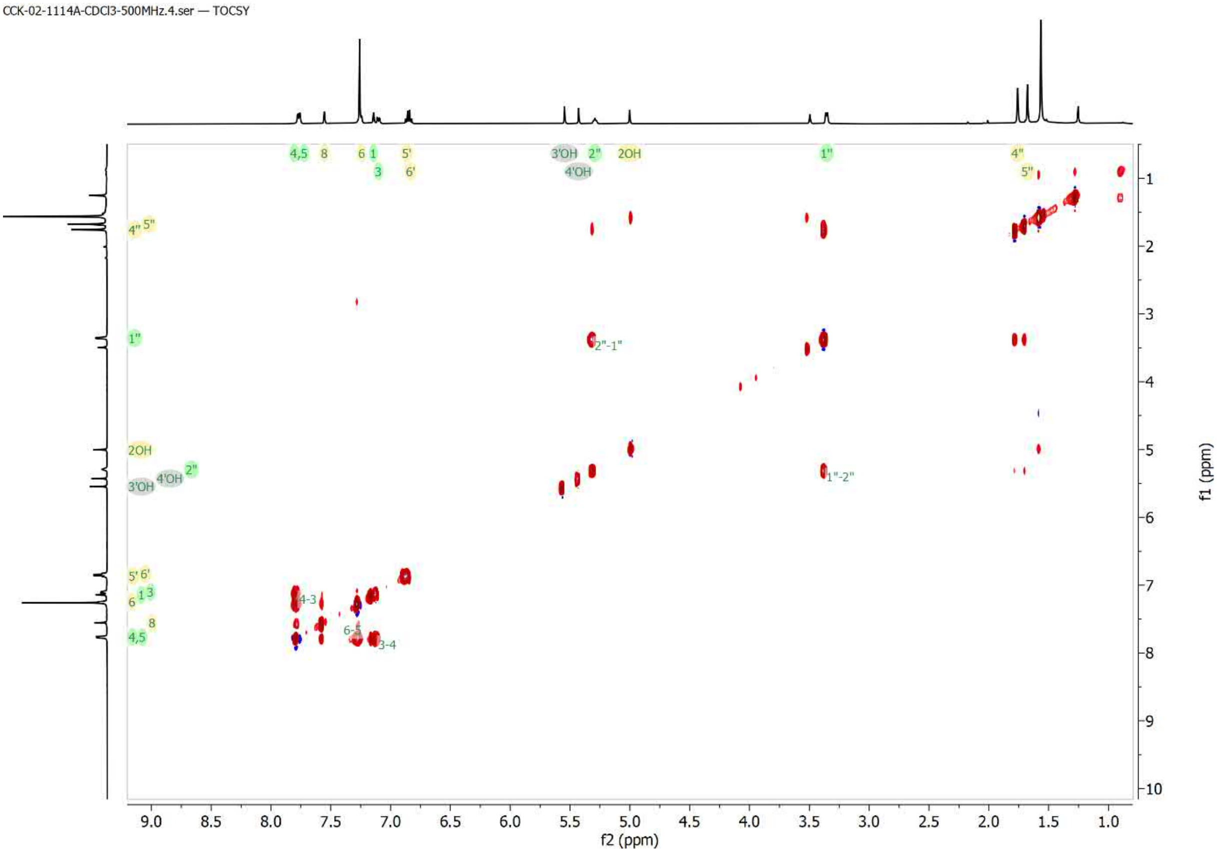

Figure S45. NOESY (500 MHz, $CDCl_3$, 25°C) spectrum of usambarin E (**5**)

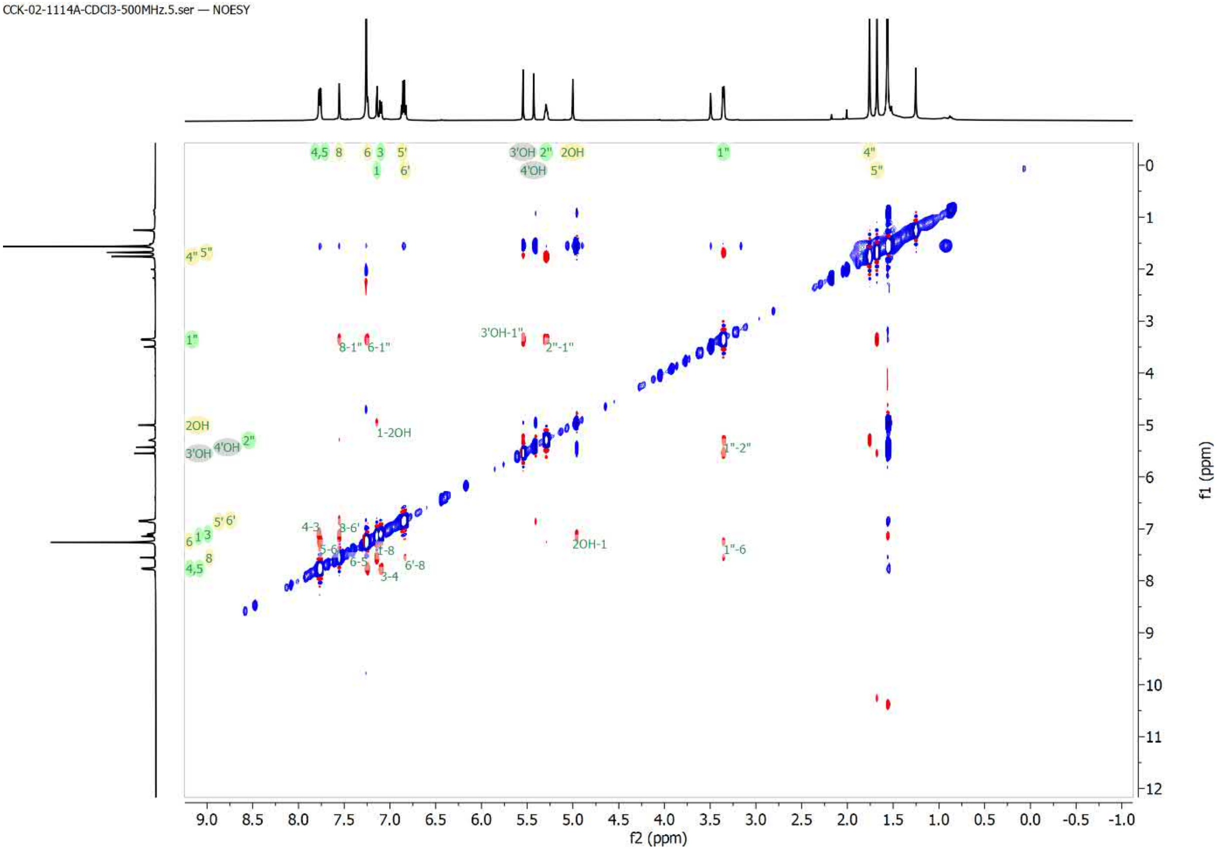

Figure S46. TOCSY (500 MHz, $CDCl_3$, 25°C) spectrum of usambarin E (**5**)

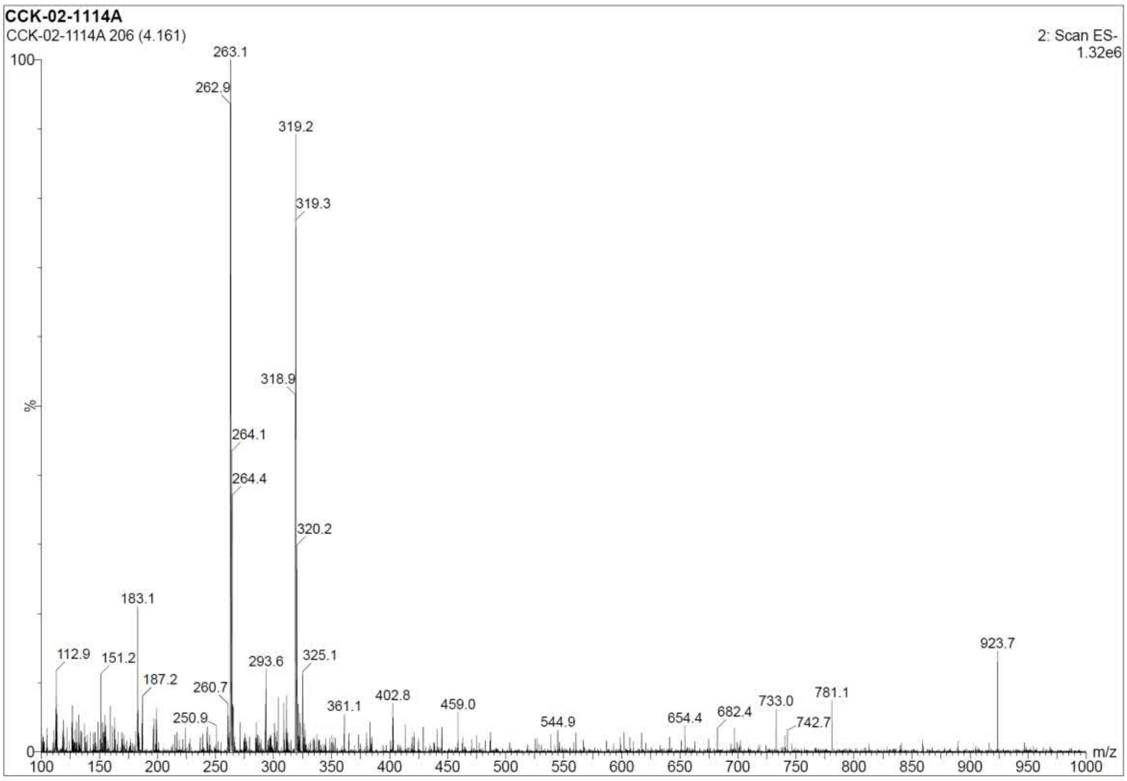

Figure S47. ESI-MS spectrum of usambarin E (**5**)

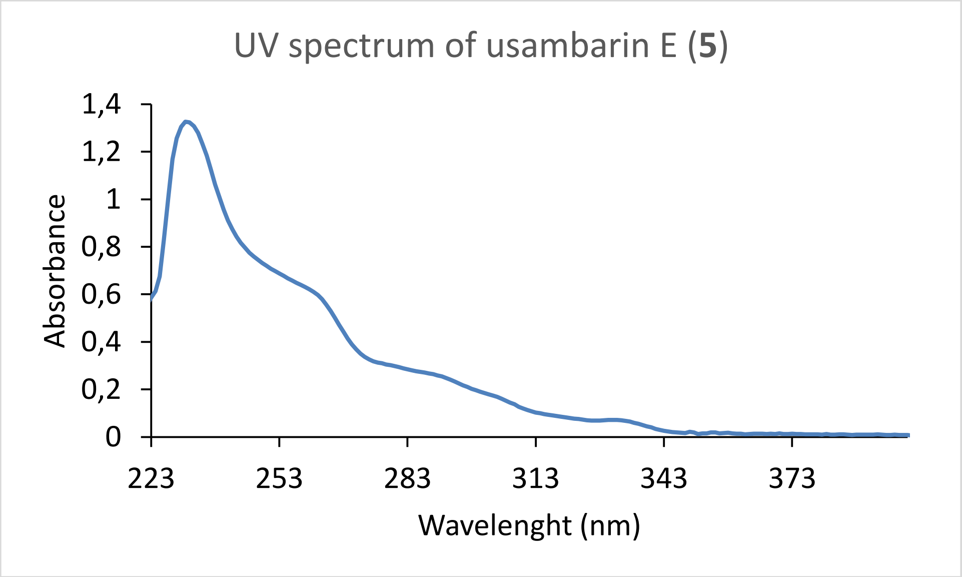

Figure S48. UV spectrum (MeOH) of usambarin E (**5**)

Spectroscopic data of Usambarin F (6)

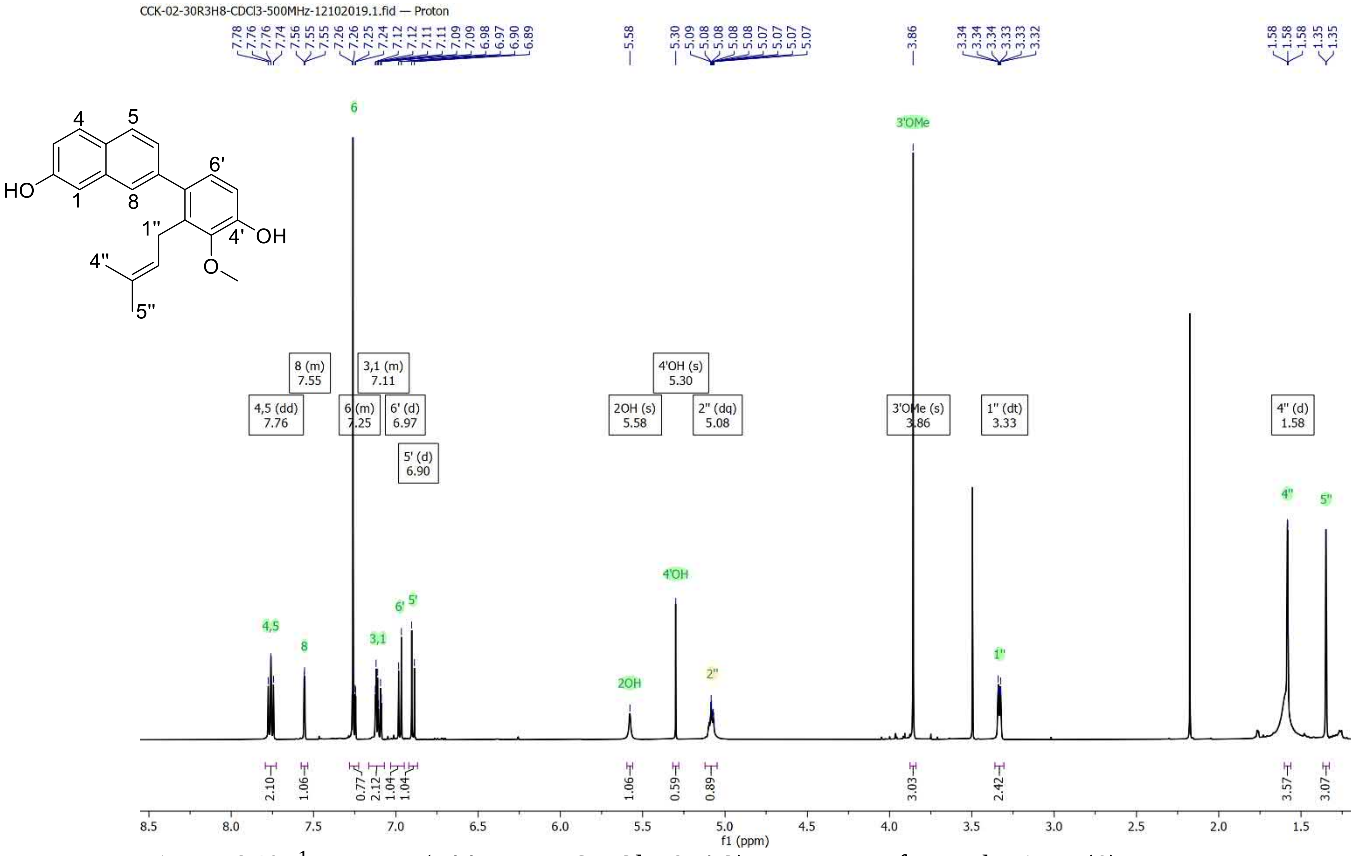

Figure S49. ^{1}H NMR (500 MHz, $CDCl_3$, 25°C) spectrum of usambarin F (**6**)

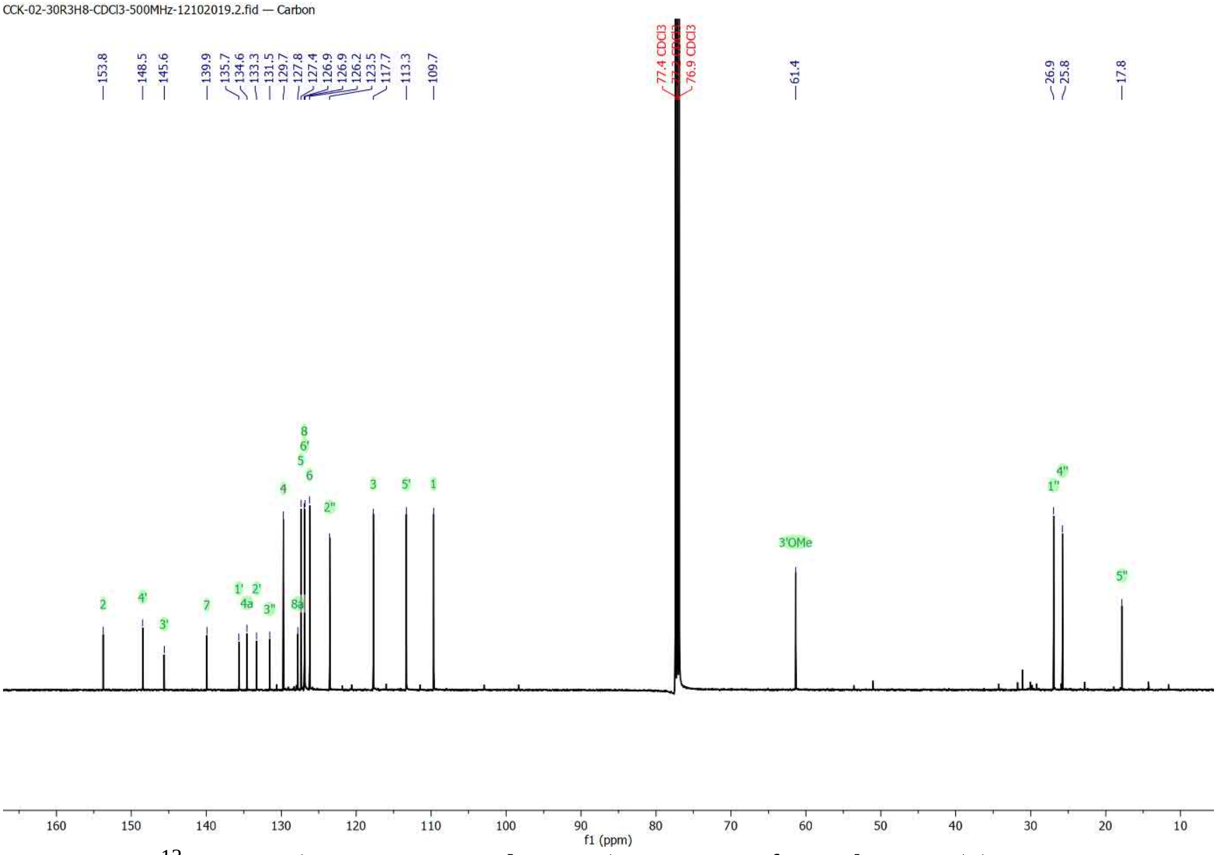

Figure S50. ^{13}C NMR (500 MHz, $CDCl_3$, 25°C) spectrum of usambarin F (**6**)

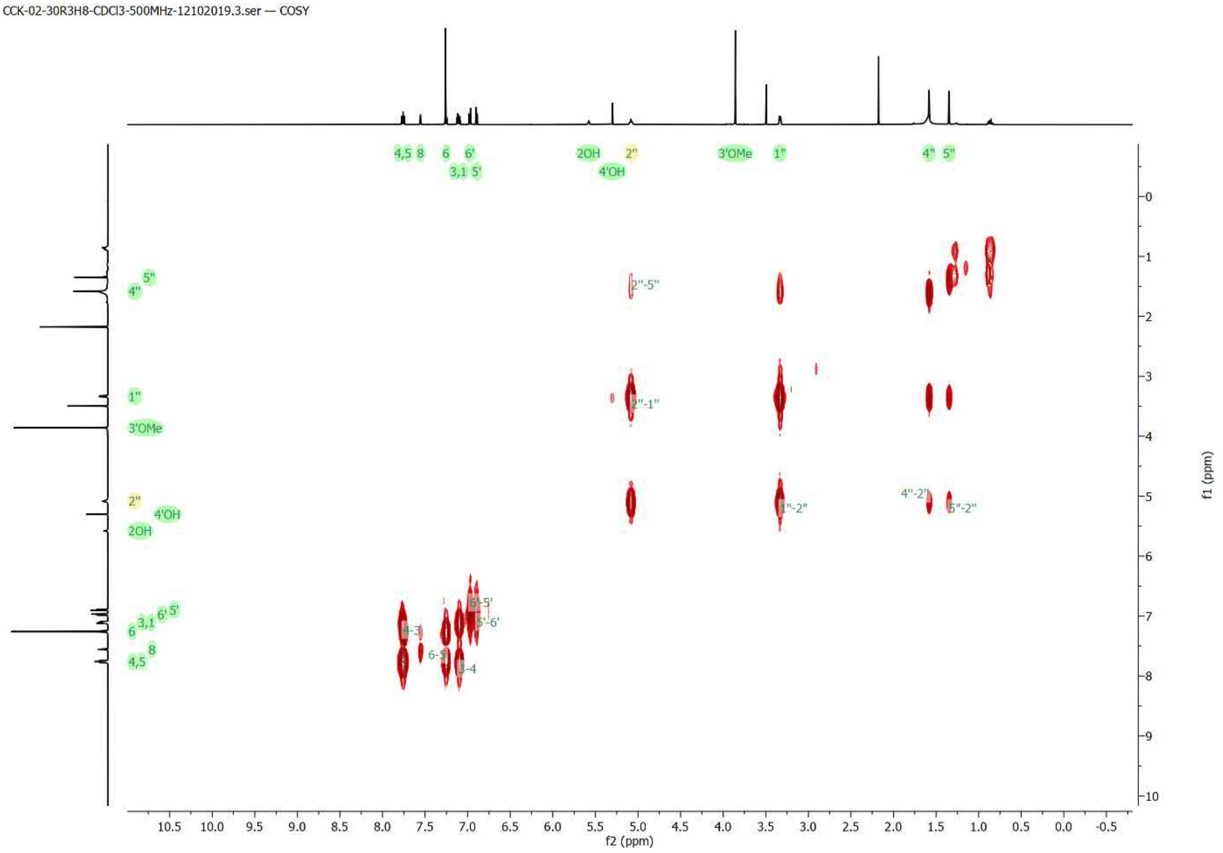

Figure S51. COSY (500 MHz, $CDCl_3$, 25°C) spectrum of usambarin F (**6**)

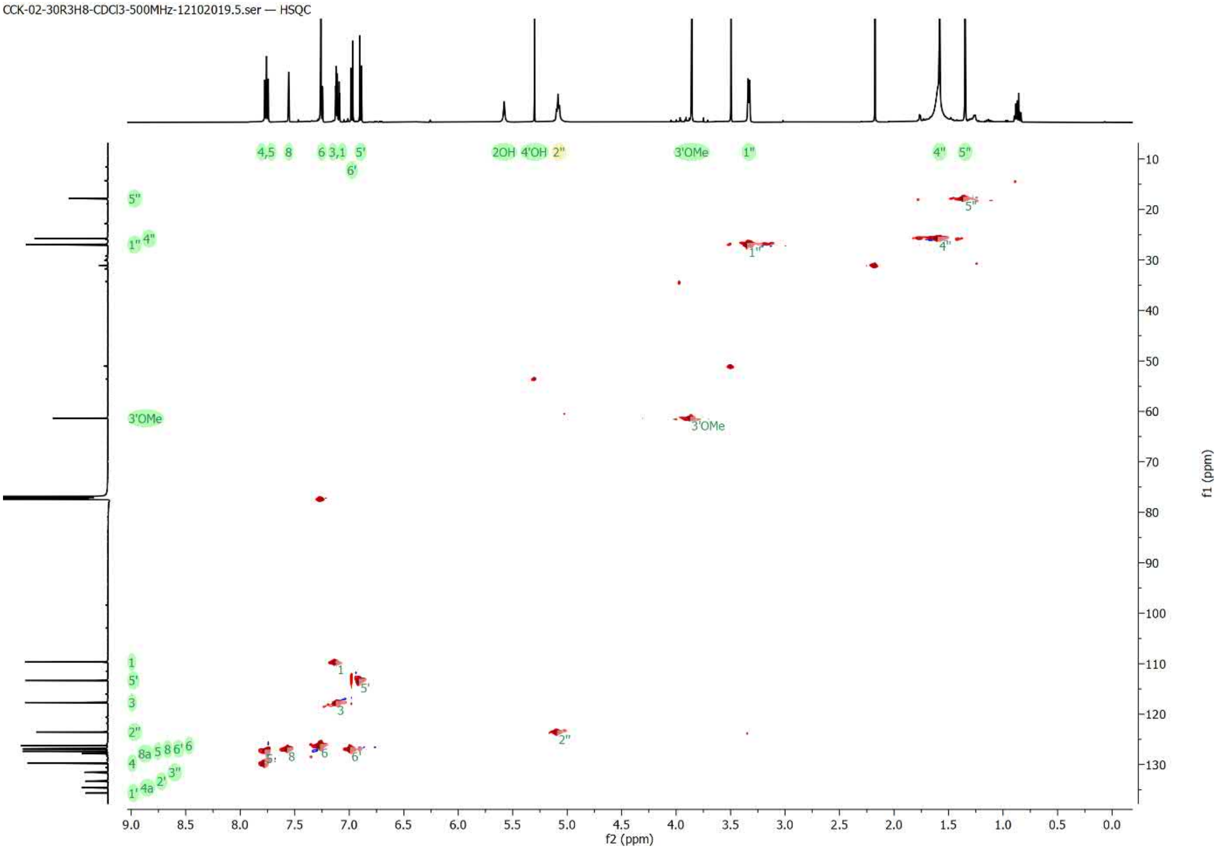

Figure S52. HSQC (500 MHz, $CDCl_3$, 25°C) spectrum of usambarin F (**6**)

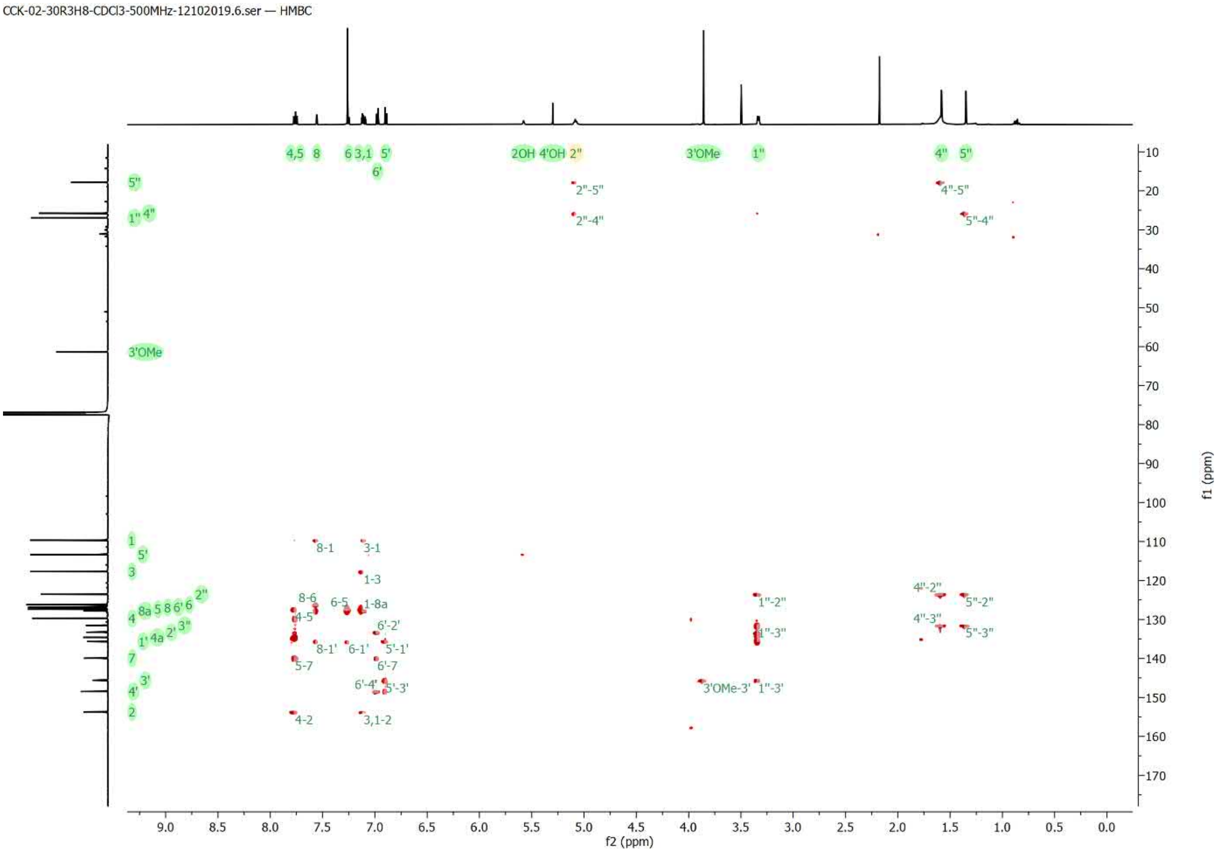

Figure S53. HMBC (500 MHz, $CDCl_3$, 25°C) spectrum of usambarin F (**6**)

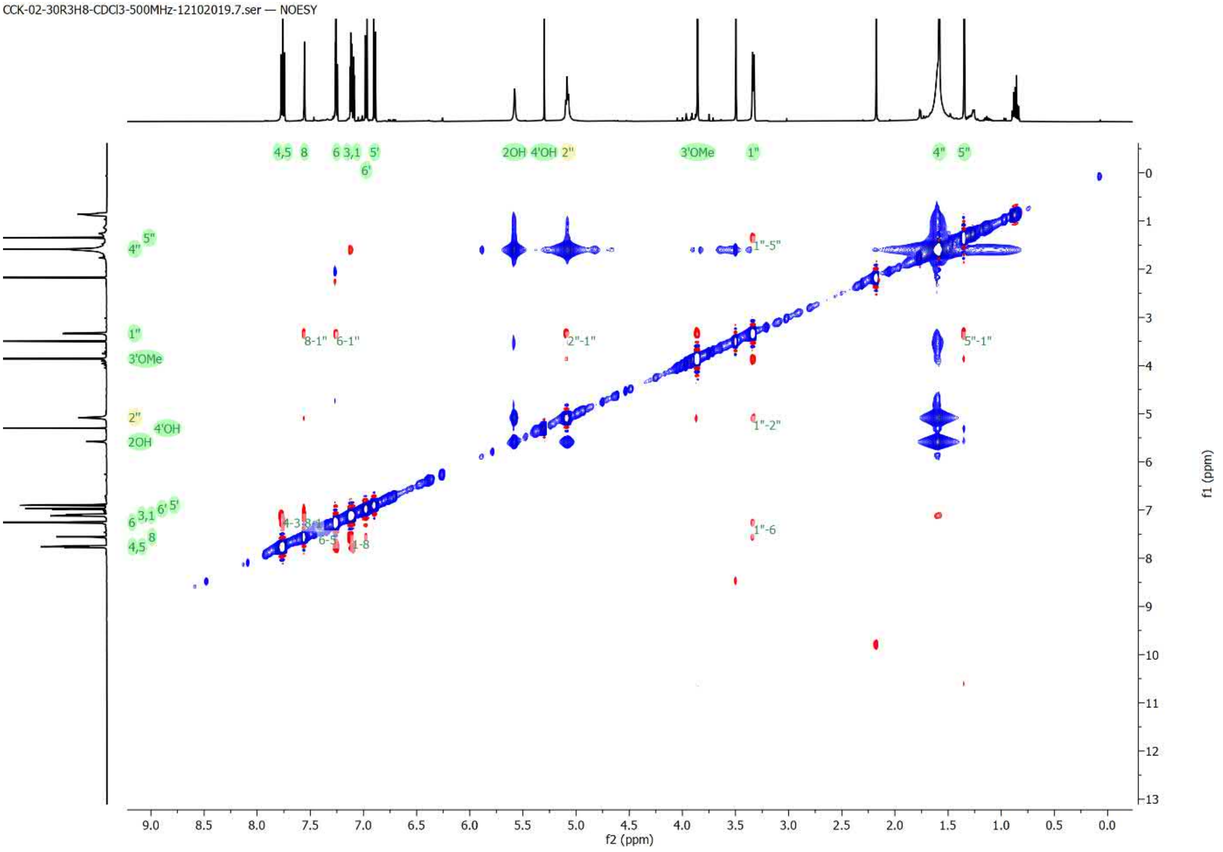

Figure S54. NOESY (500 MHz, $CDCl_3$, 25°C) spectrum of usambarin F (**6**)

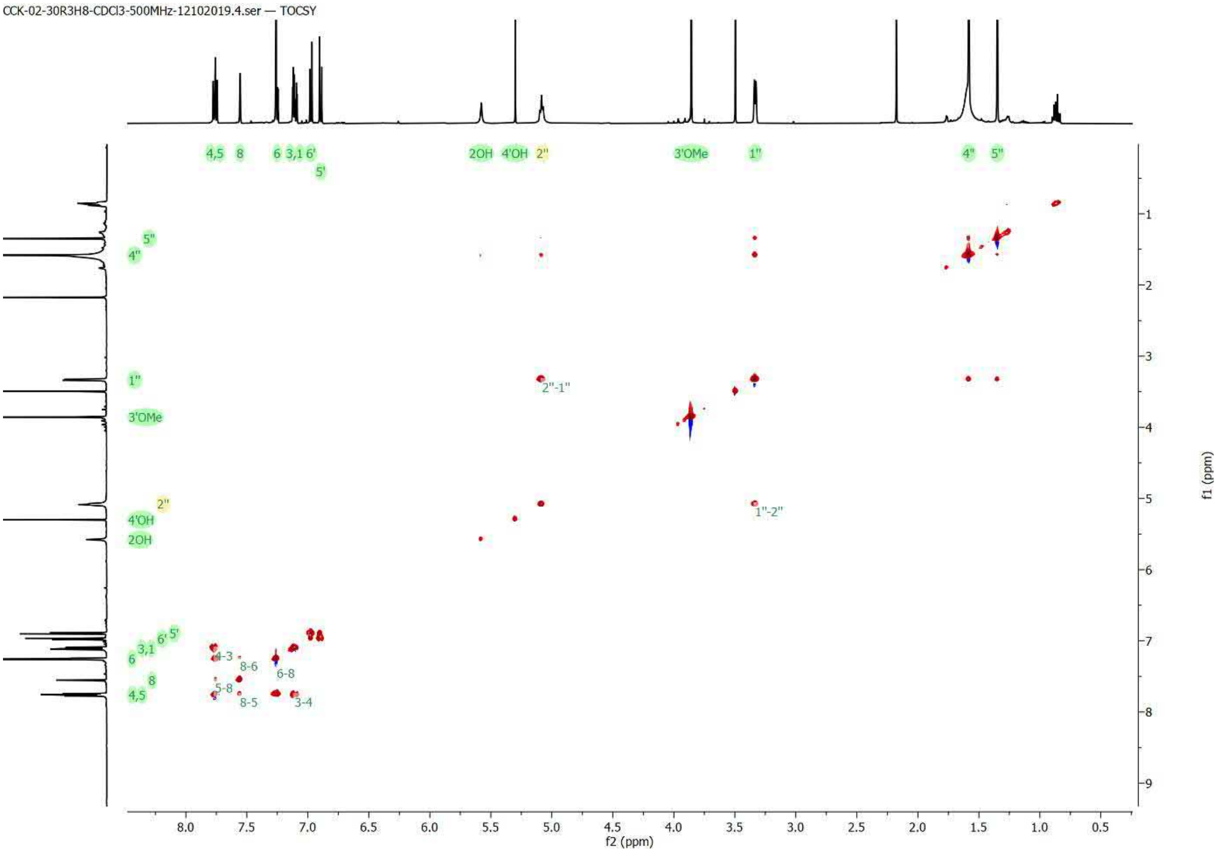

Figure S55.TOCSY (500 MHz, $CDCl_3$, 25°C) spectrum of usambarin F (**6**)

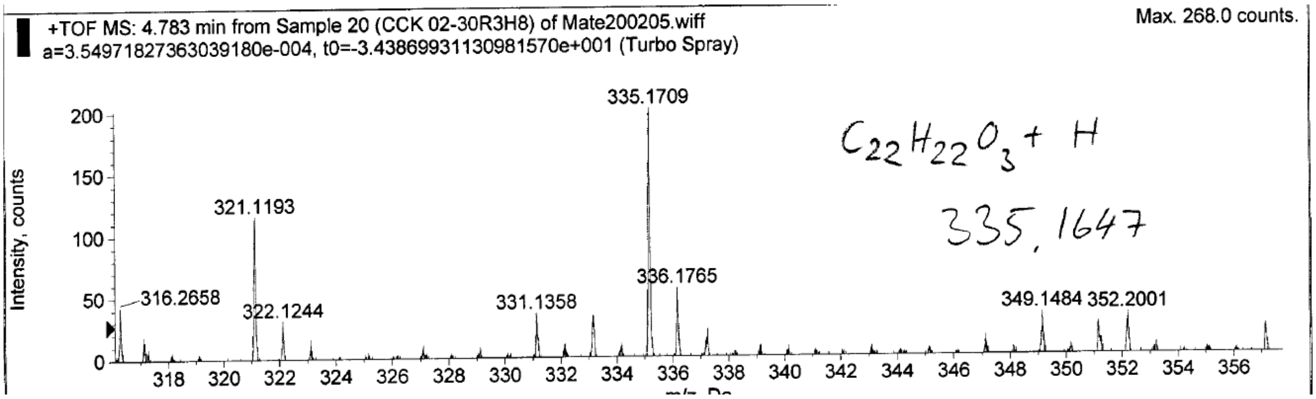

Figure S56. HRMS spectrum of usambarin F (**6**)

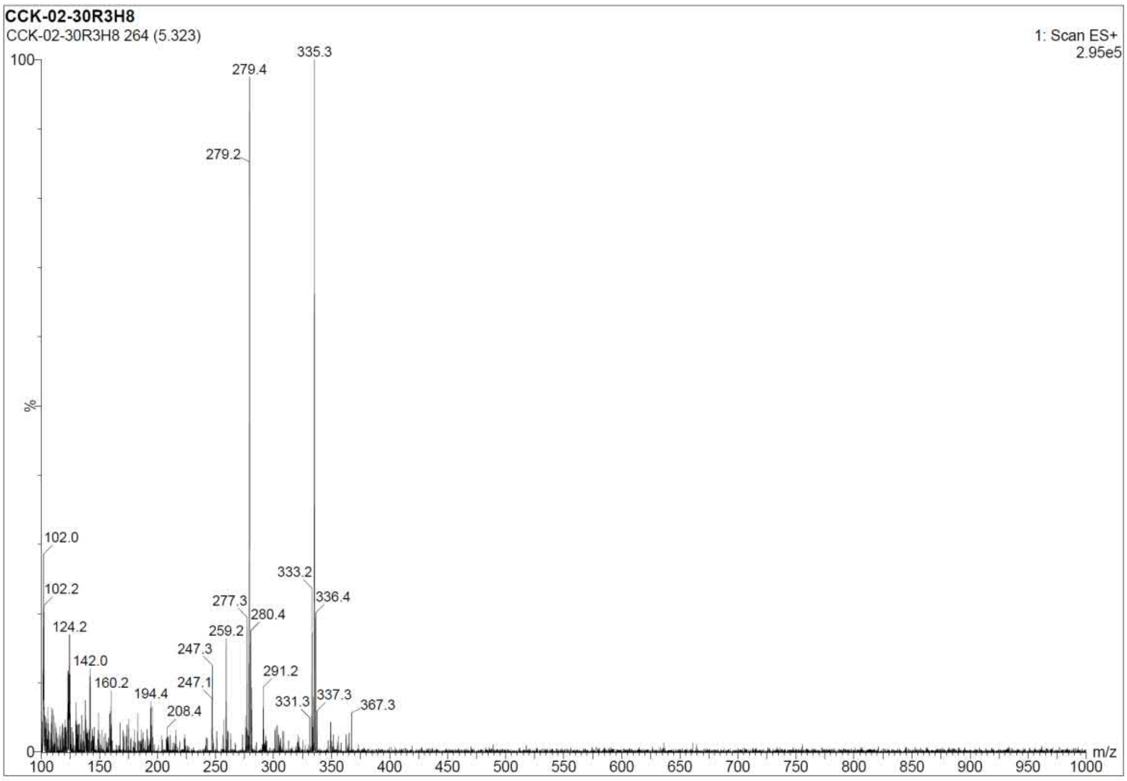

Figure S57. ESI-MS spectrum of usambarin F (**6**)

Spectroscopic data of Usambarin G (7)

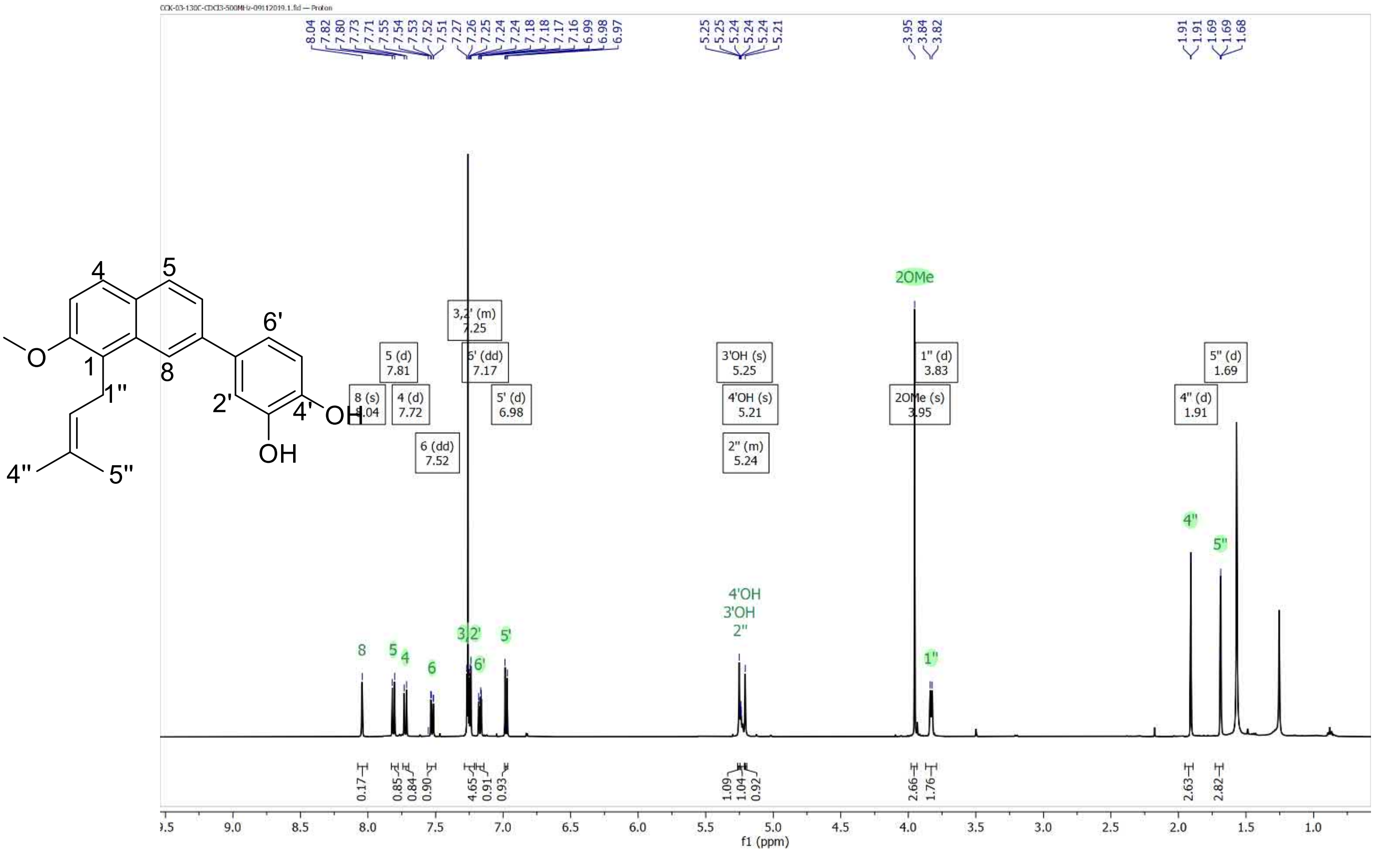

Figure S58. ^{1}H NMR (500 MHz, $CDCl_3$, 25°C) spectrum of usambarin G (**7**)

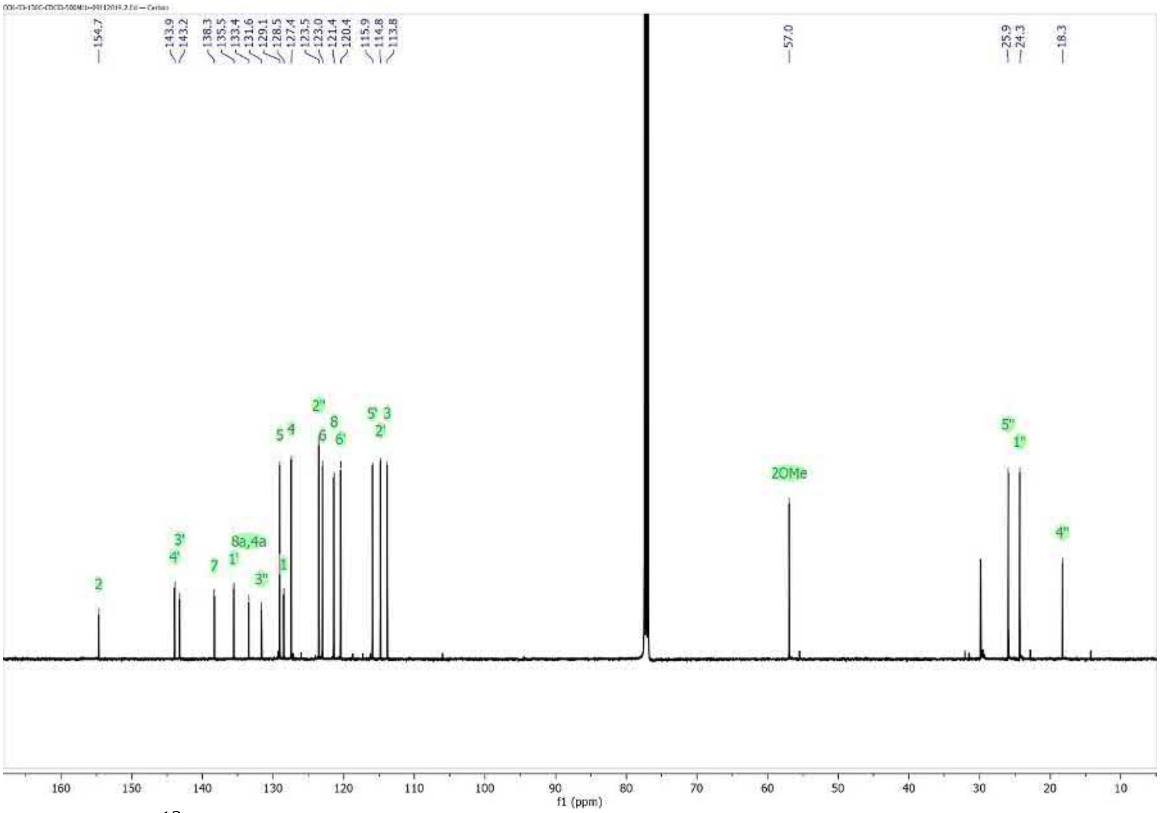

Figure S59. ^{13}C NMR (500 MHz, $CDCl_3$, 25°C) spectrum of usambarin G (**7**)

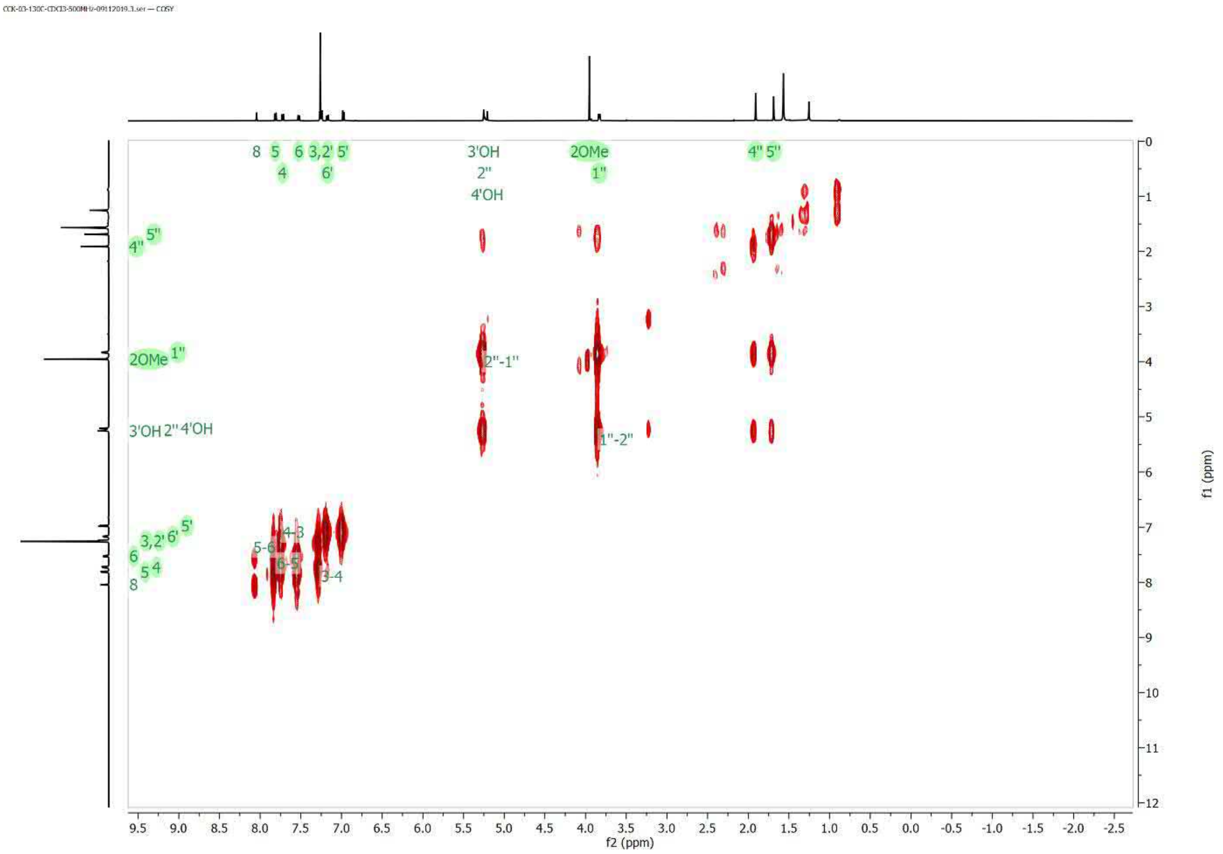

Figure S60. COSY (500 MHz, $CDCl_3$, 25°C) spectrum of usambarin G (**7**)

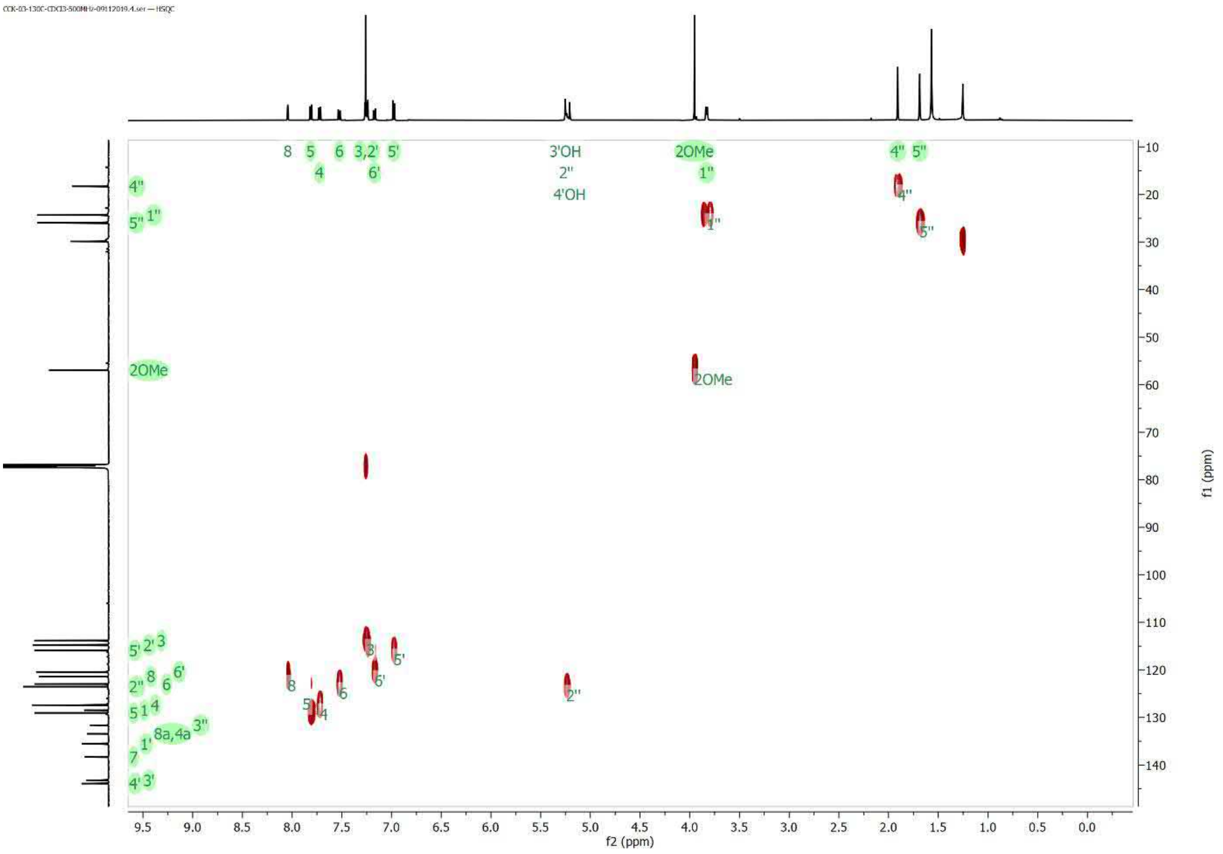

Figure S61. HSQC (500 MHz, $CDCl_3$, 25°C) spectrum of usambarin G (**7**)

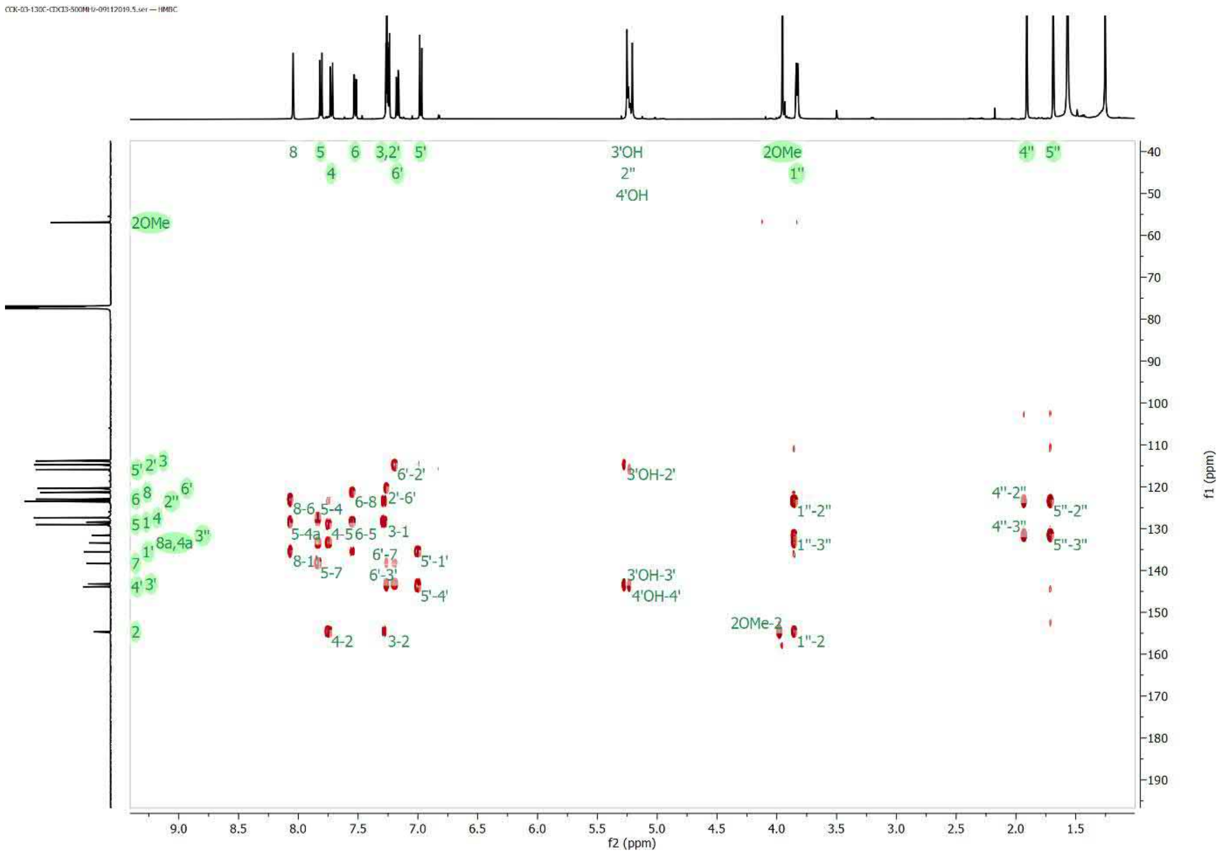

Figure S62. HMBC (500 MHz, $CDCl_3$, 25°C) spectrum of usambarin G (**7**)

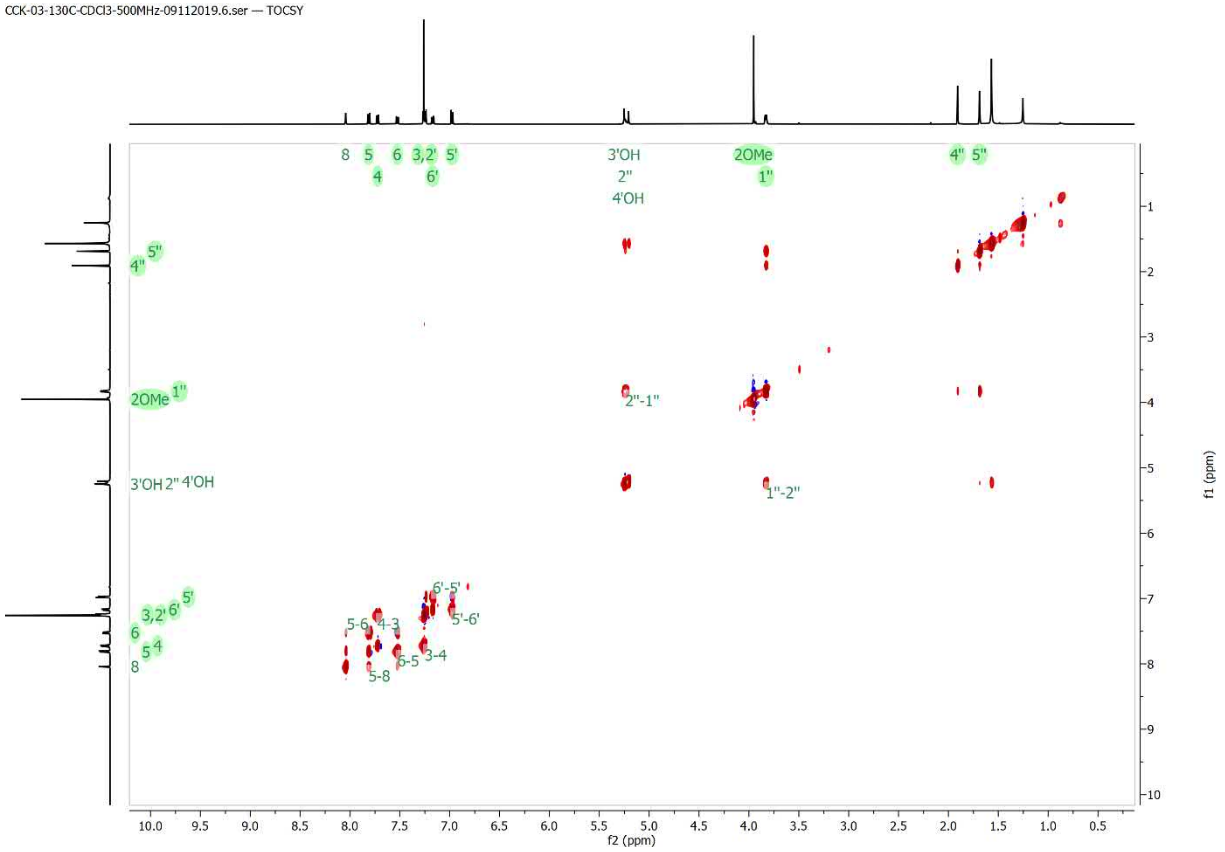

Figure S63. TOCSY (500 MHz, $CDCl_3$, 25°C) spectrum of usambarin G (**7**)

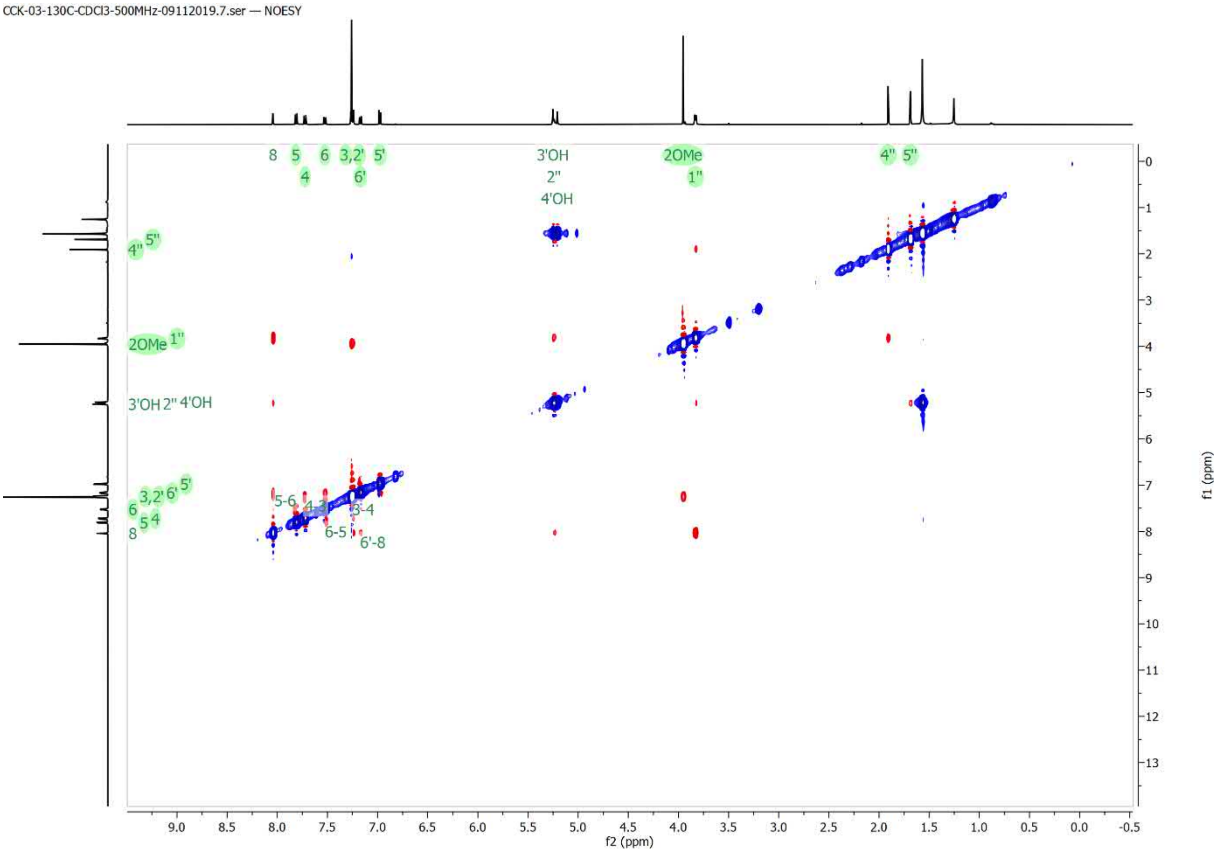

Figure S64. NOESY (500 MHz, $CDCl_3$, 25°C) spectrum of usambarin G (**7**)

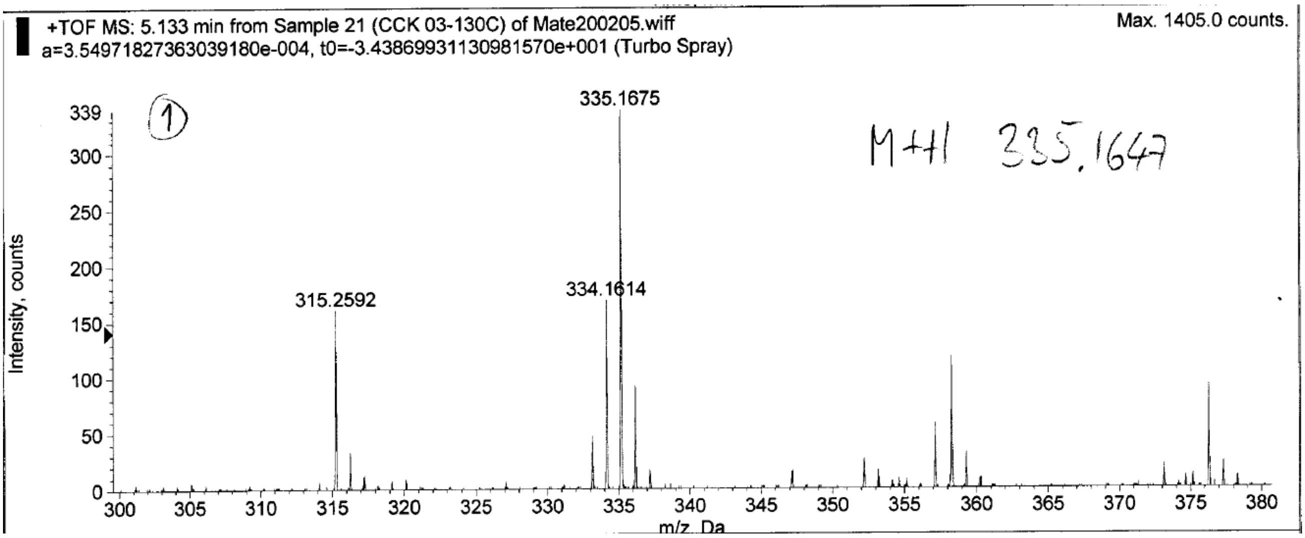

Figure S65. HRMS spectrum of usambarin G (**7**)

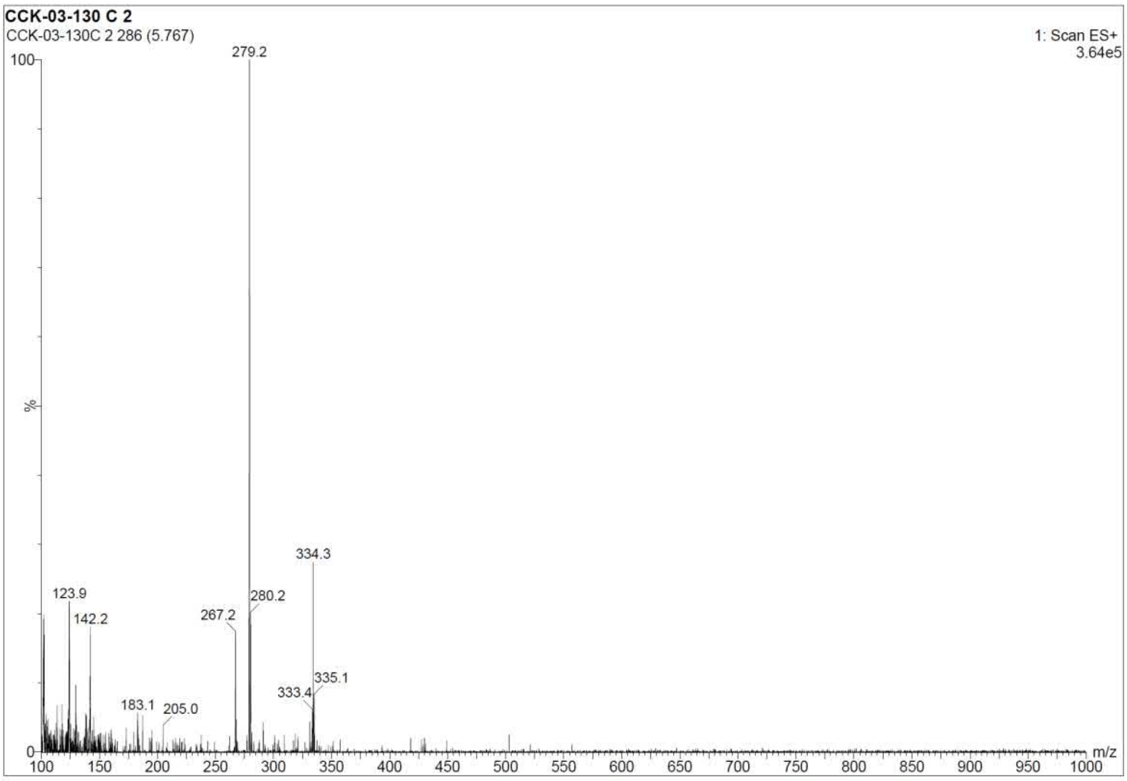

Figure S66. ESI-MS spectrum of usambarin G (**7**)

Spectroscopic data of Usambarin H (8)

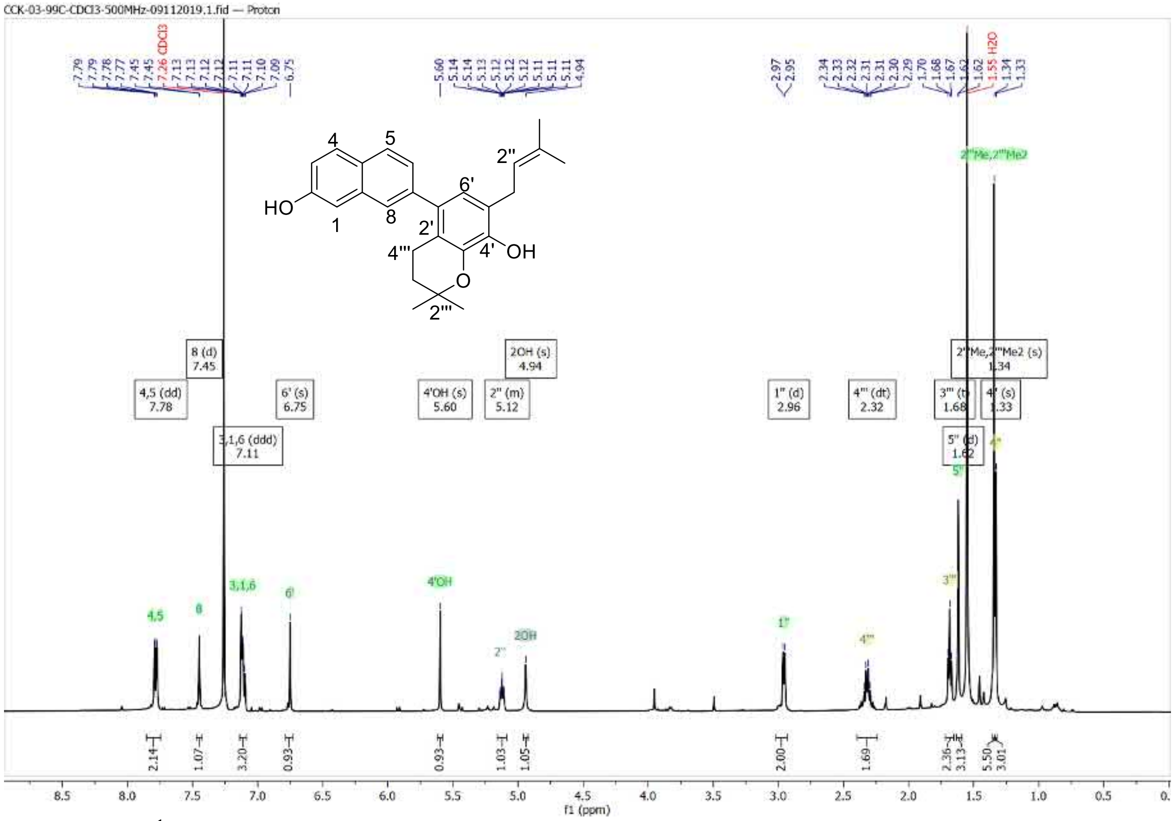

Figure S67. ^{1}H NMR (500 MHz, $CDCl_3$, 25°C) spectrum of usambarin H (**8**)

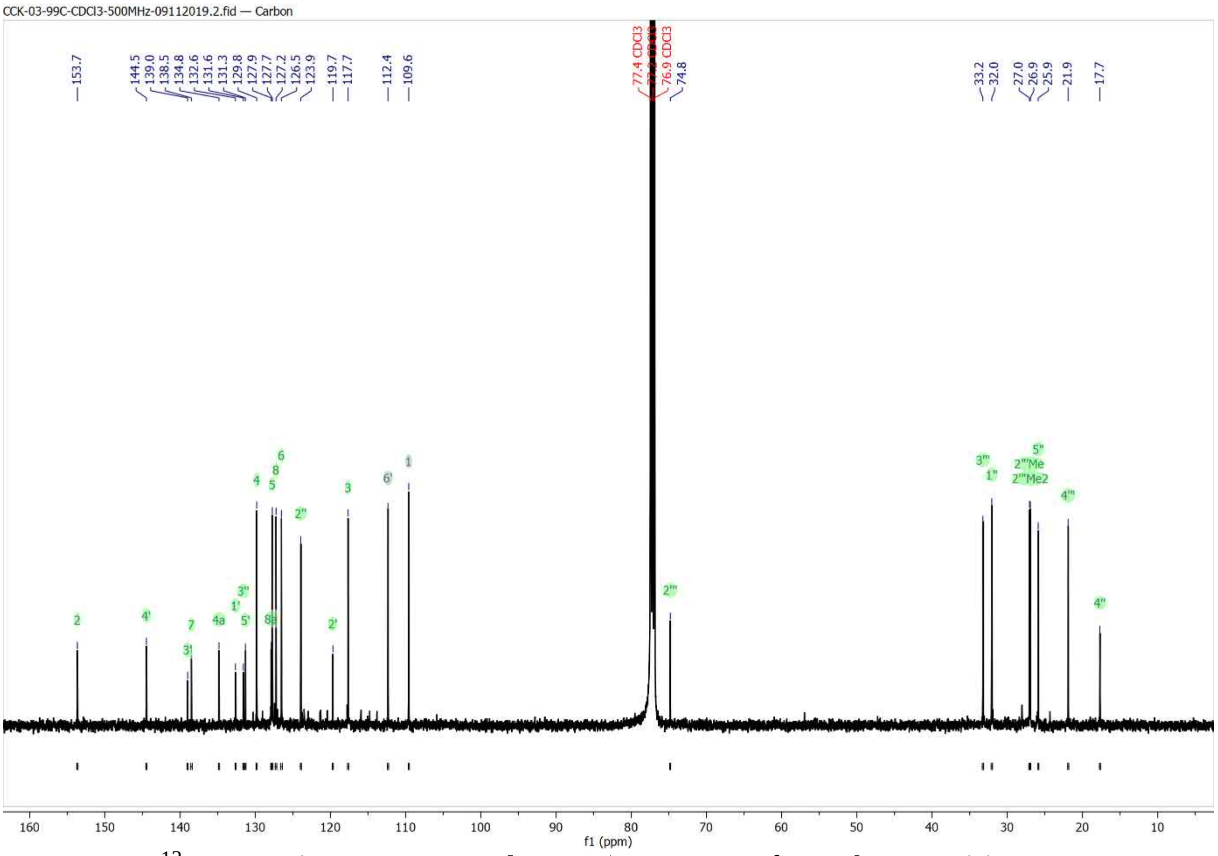

Figure S68. ^{13}C NMR (500 MHz, $CDCl_3$, 25°C) spectrum of usambarin H (**8**)

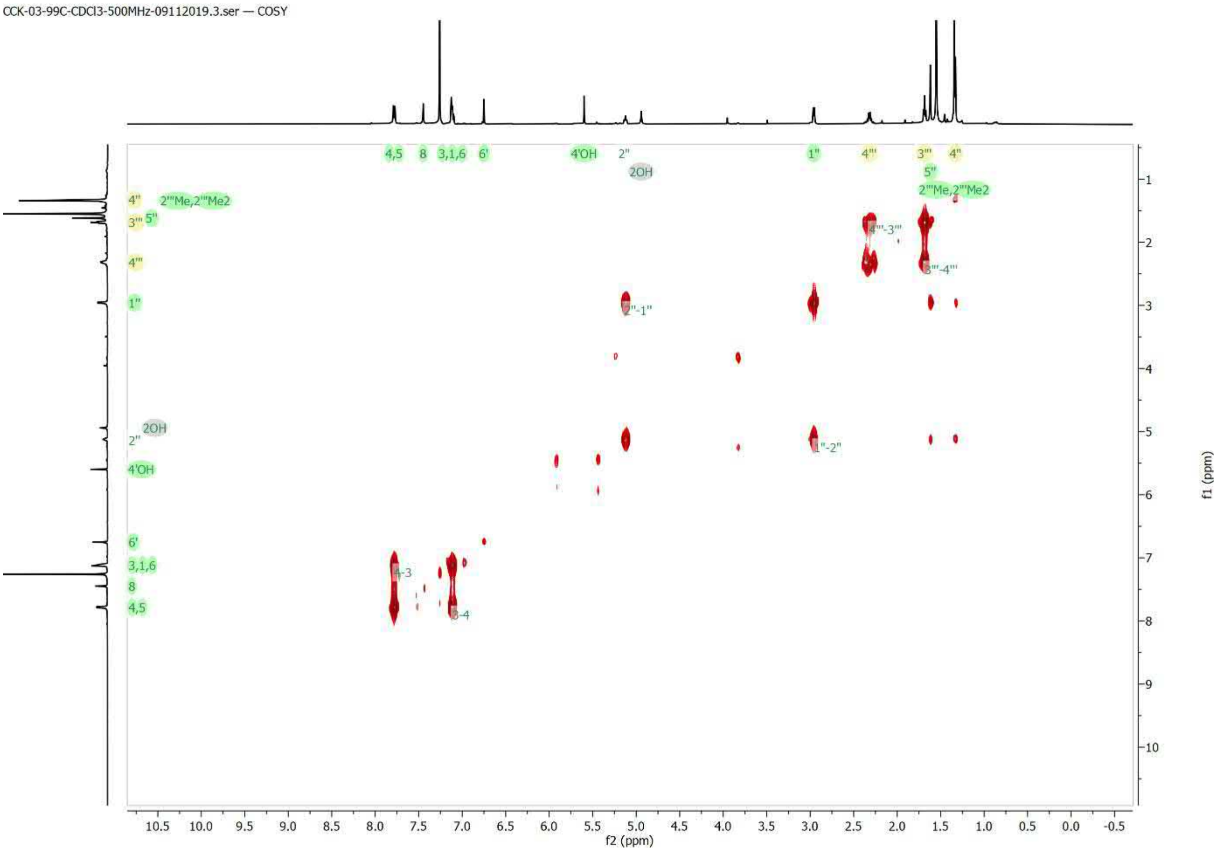

Figure S69. COSY (500 MHz, $CDCl_3$, 25°C) spectrum of usambarin H (**8**)

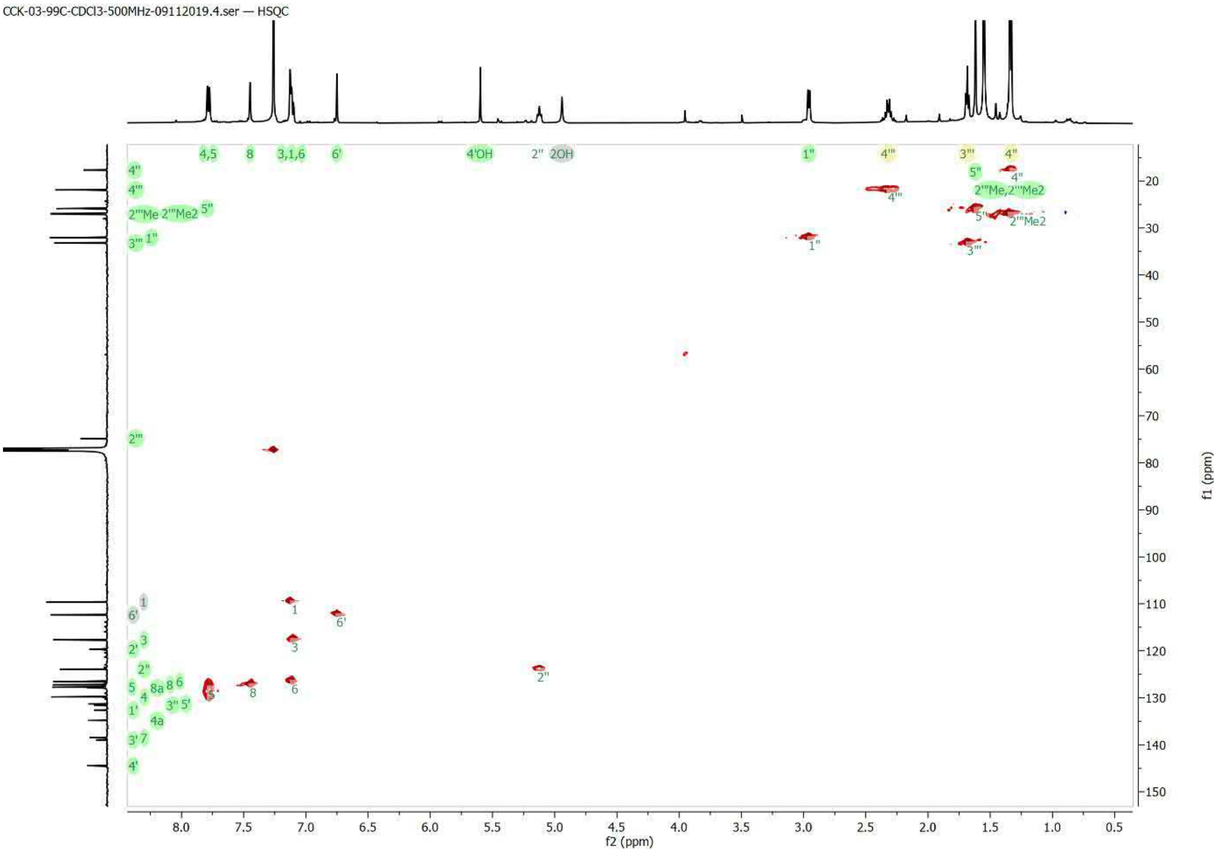

Figure S70. HSQC (500 MHz, $CDCl_3$, 25°C) spectrum of usambarin H (**8**)

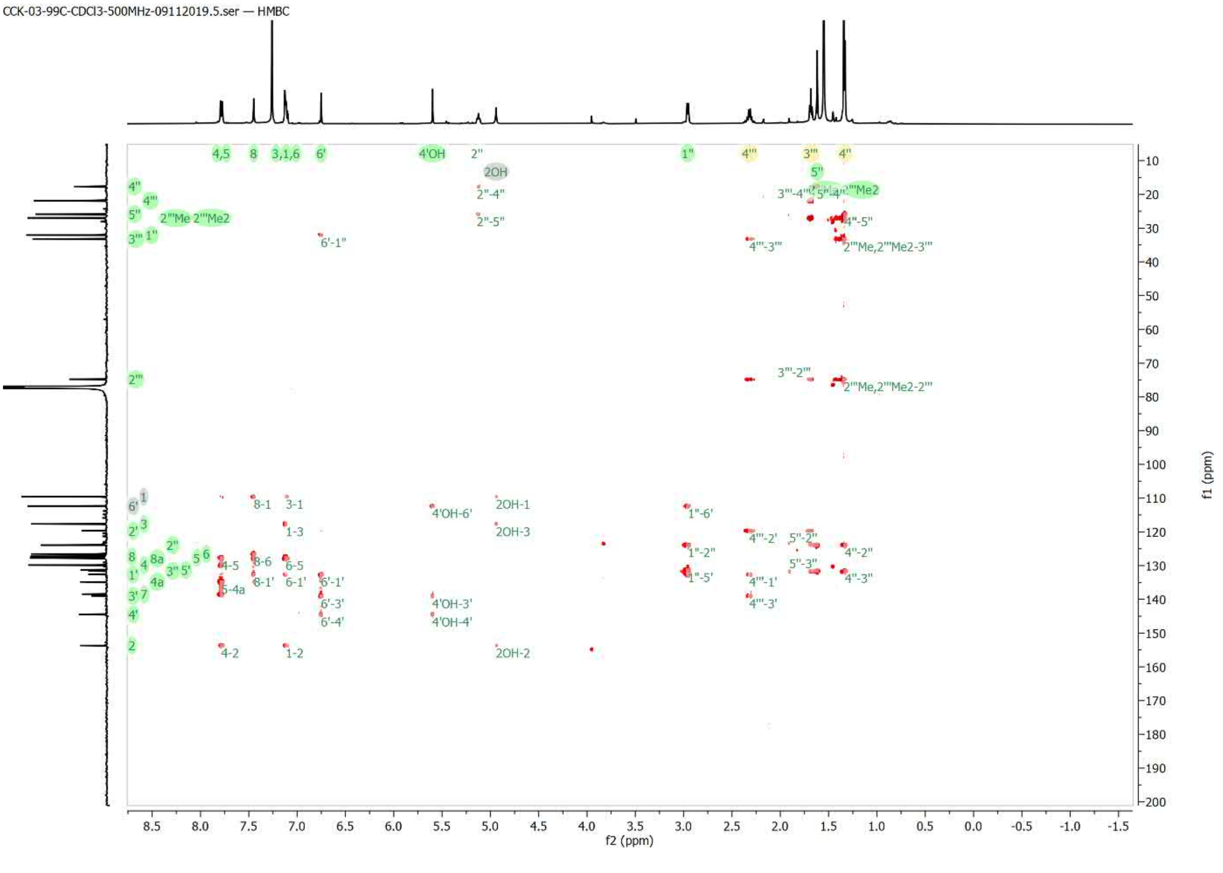

Figure S71. HMBC (500 MHz, $CDCl_3$, 25°C) spectrum of usambarin H (**8**)

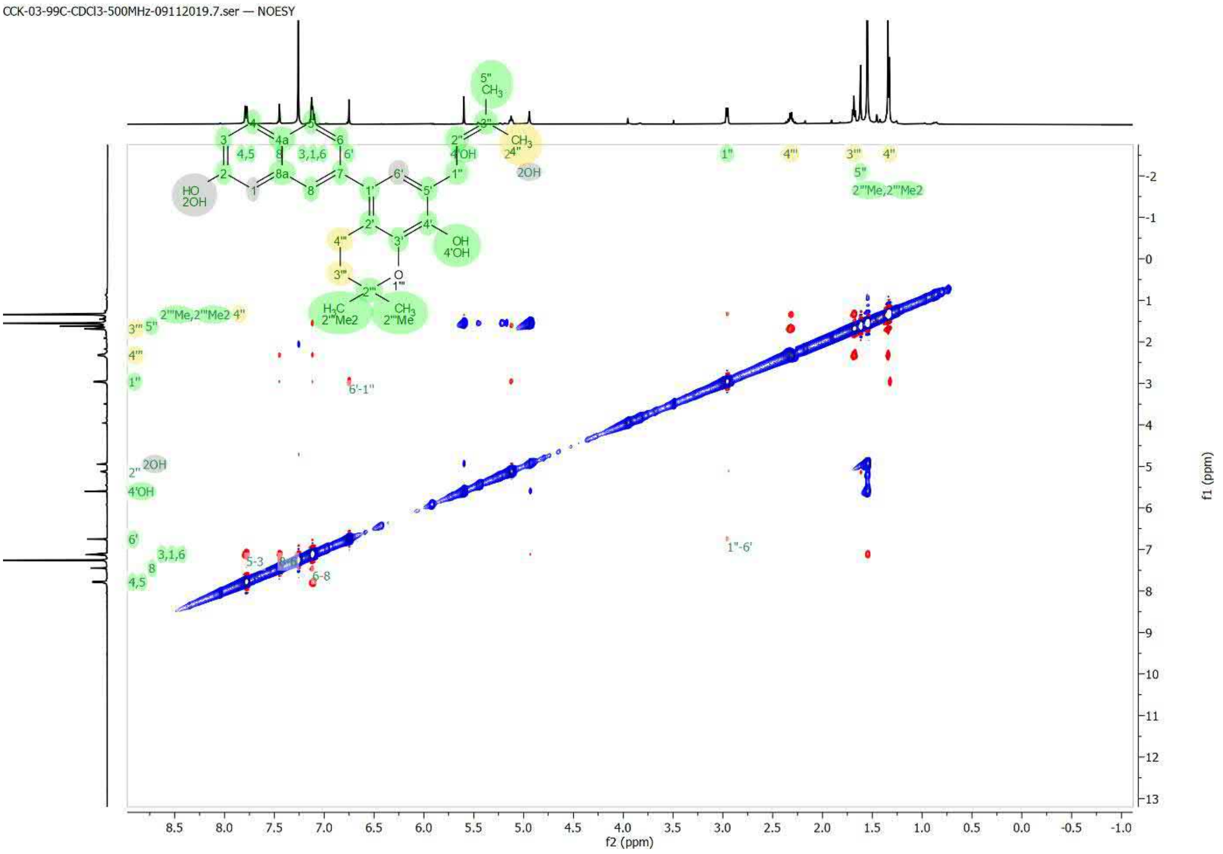

Figure S72. NOESY (500 MHz, $CDCl_3$, 25°C) spectrum of usambarin H (**8**)

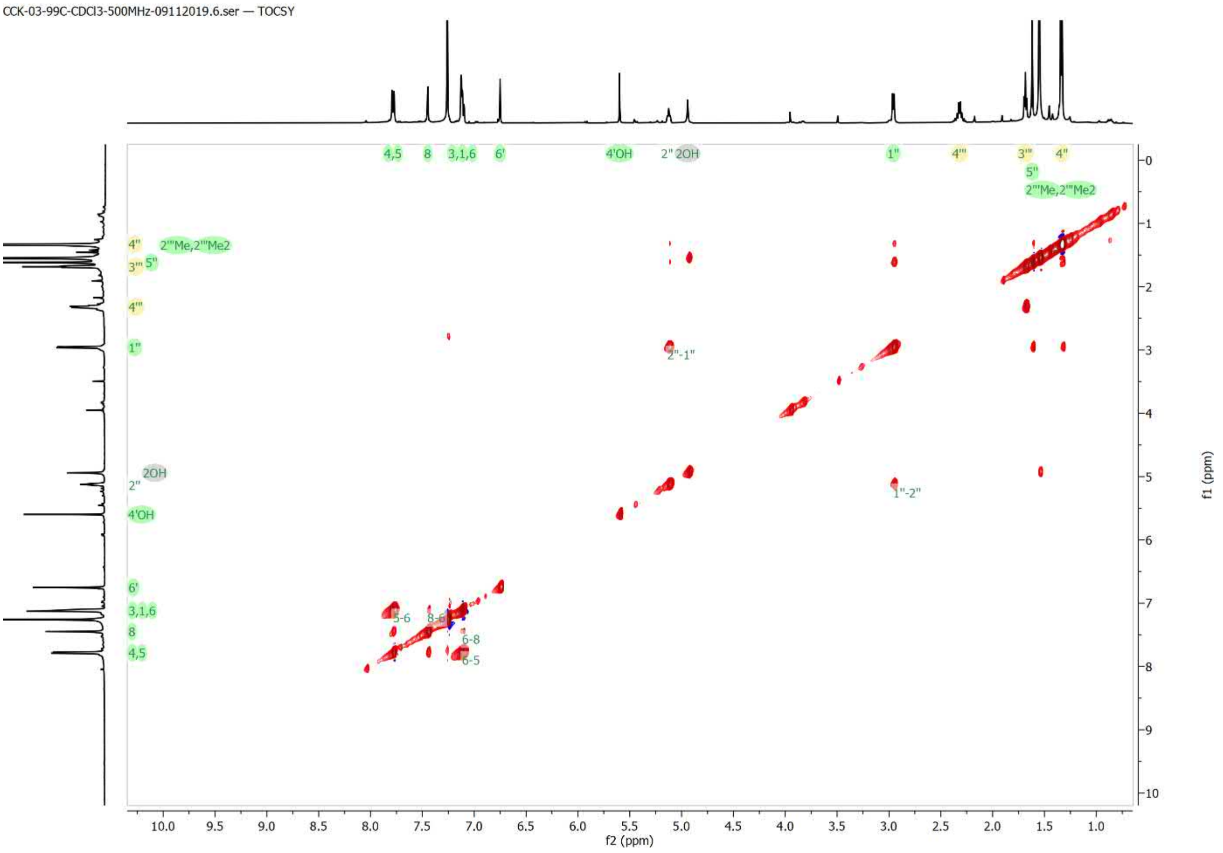

Figure S73. TOCSY (500 MHz, $CDCl_3$, 25°C) spectrum of usambarin H (**8**)

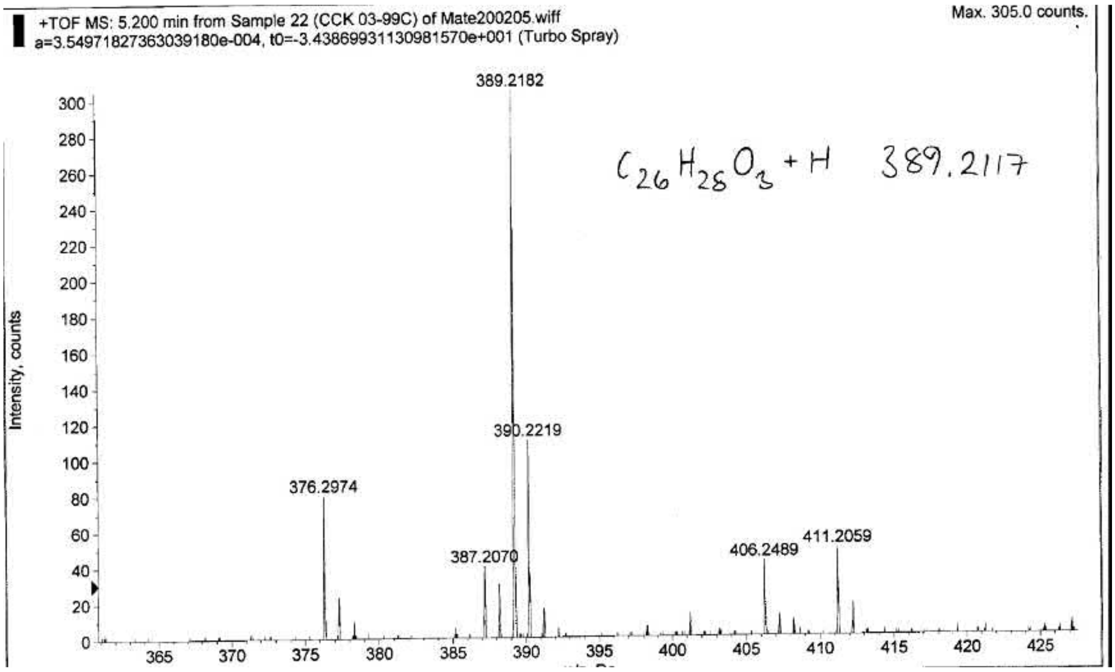

Figure S74. HRMS spectrum of usambarin H (**8**)

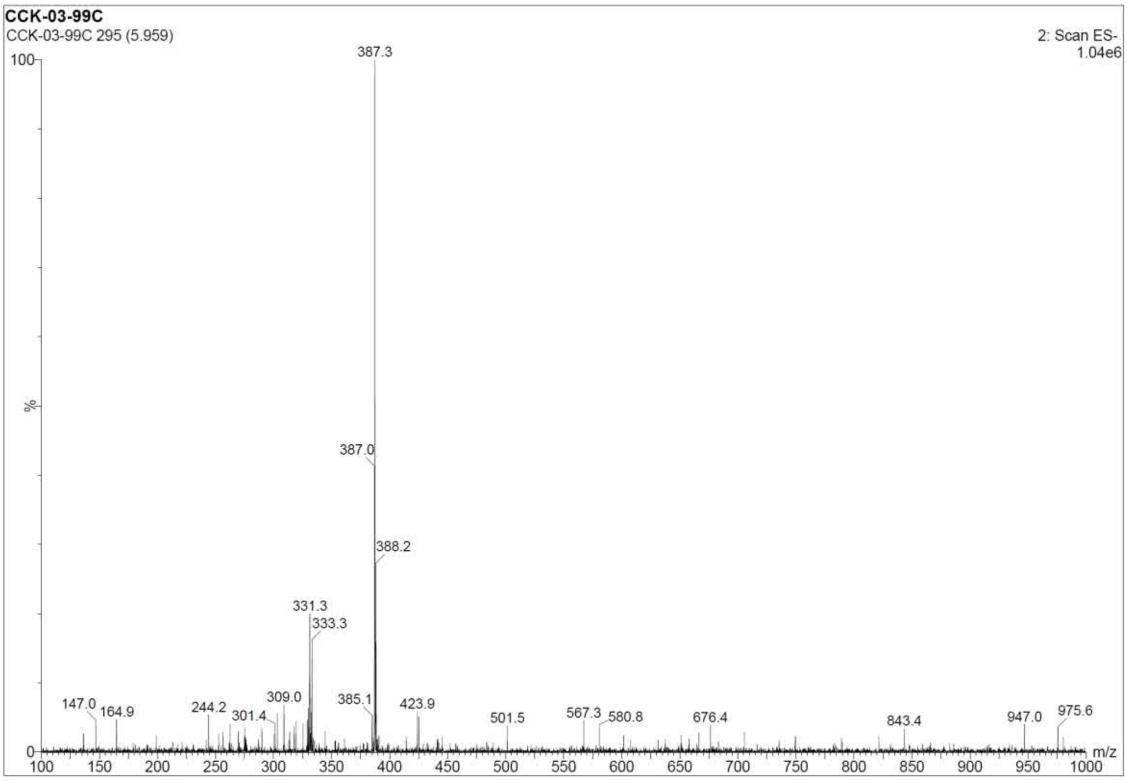

Figure S75. ESI-MS spectrum of usambarin H (**8**)

Spectroscopic data of Usambarin J (9)

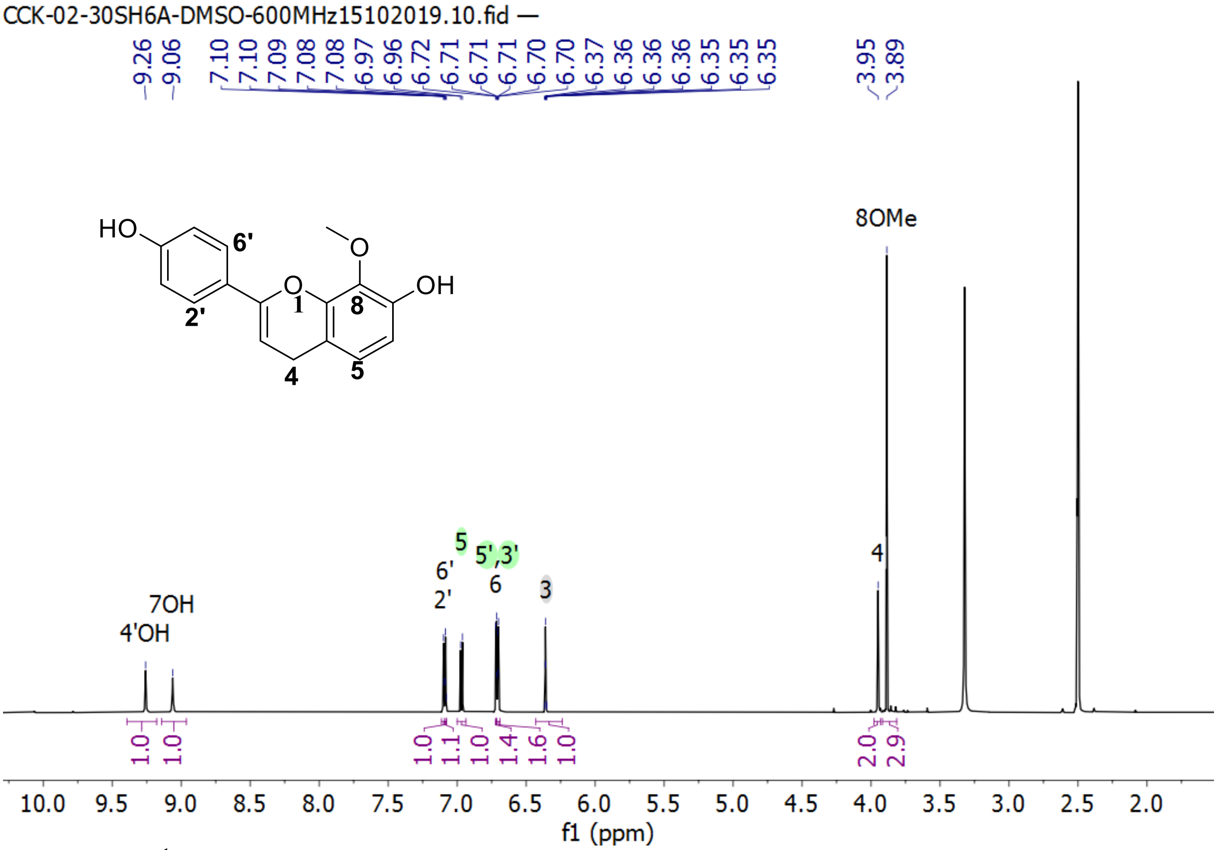

Figure S76. ^{1}H NMR (600 MHz, DMSO, 25°C) spectrum of usambarin J (**9**)

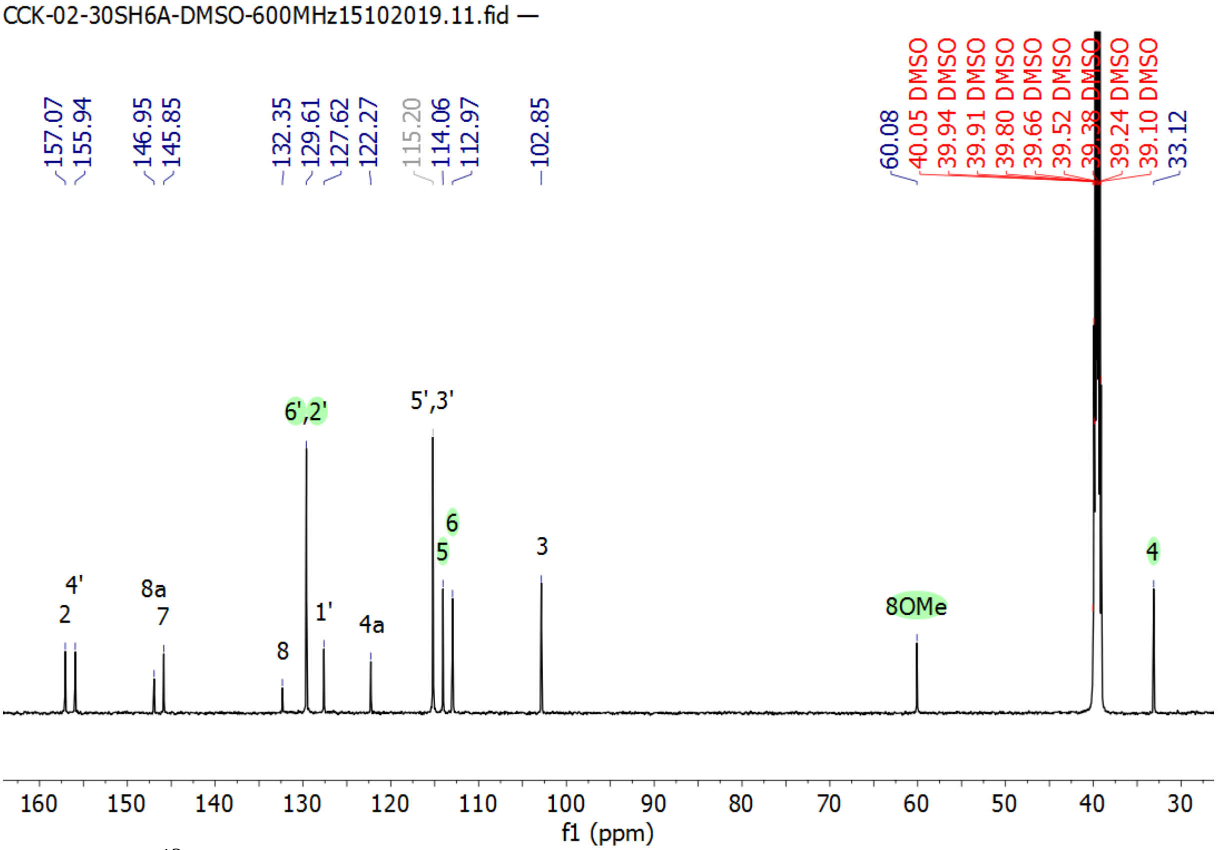

Figure S77. ^{13}C NMR (600 MHz, DMSO, 25°C) spectrum of usambarin J (**9**)

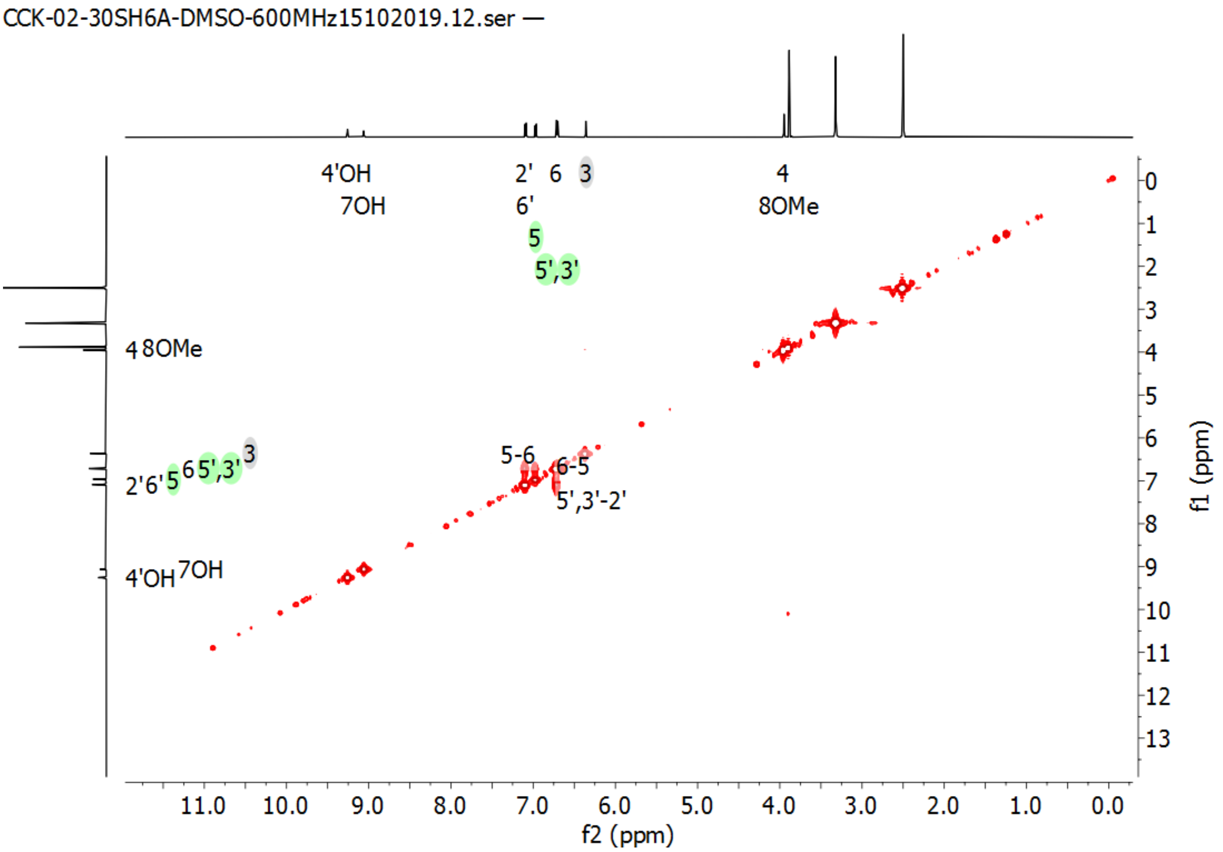

Figure S78. COSY (600 MHz, DMSO, 25°C) spectrum of usambarin J (**9**)

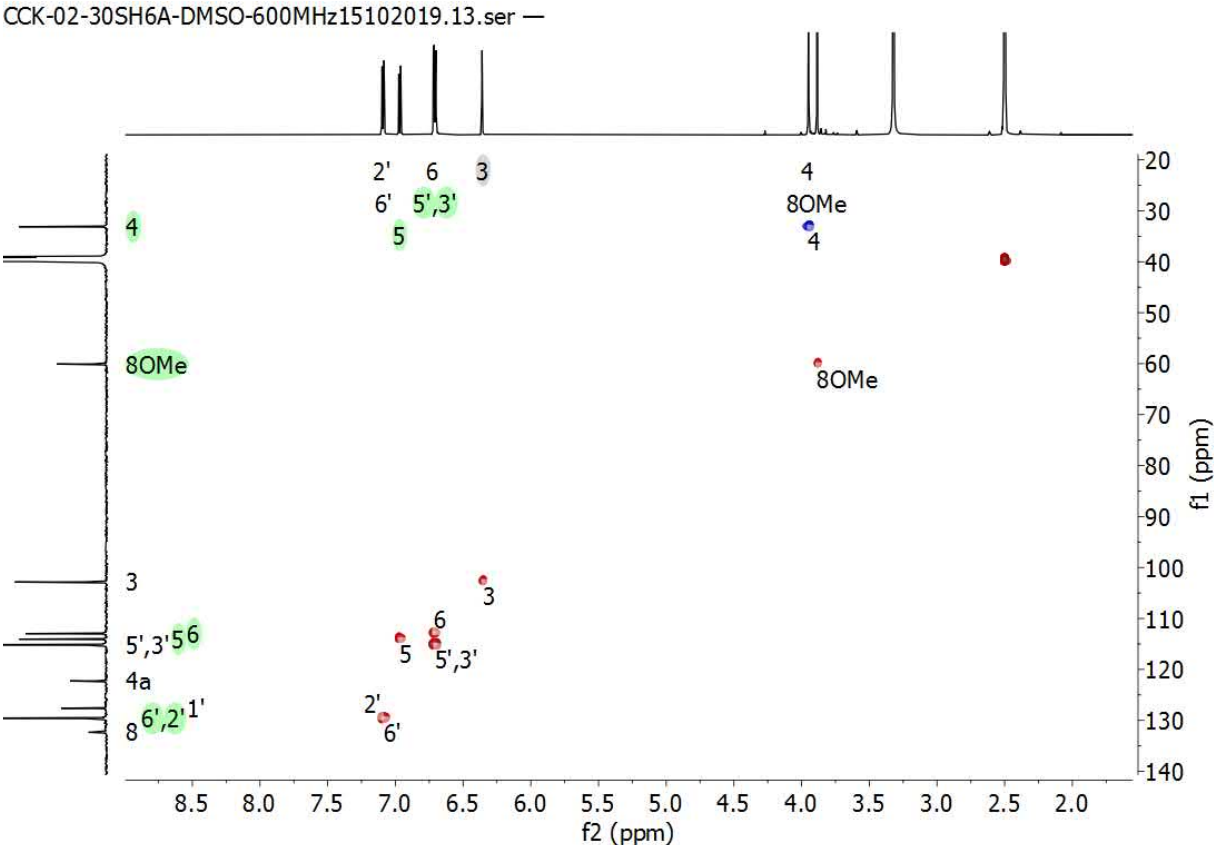

Figure S79. HSQC (600 MHz, DMSO, 25°C) spectrum of usambarin J (**9**)

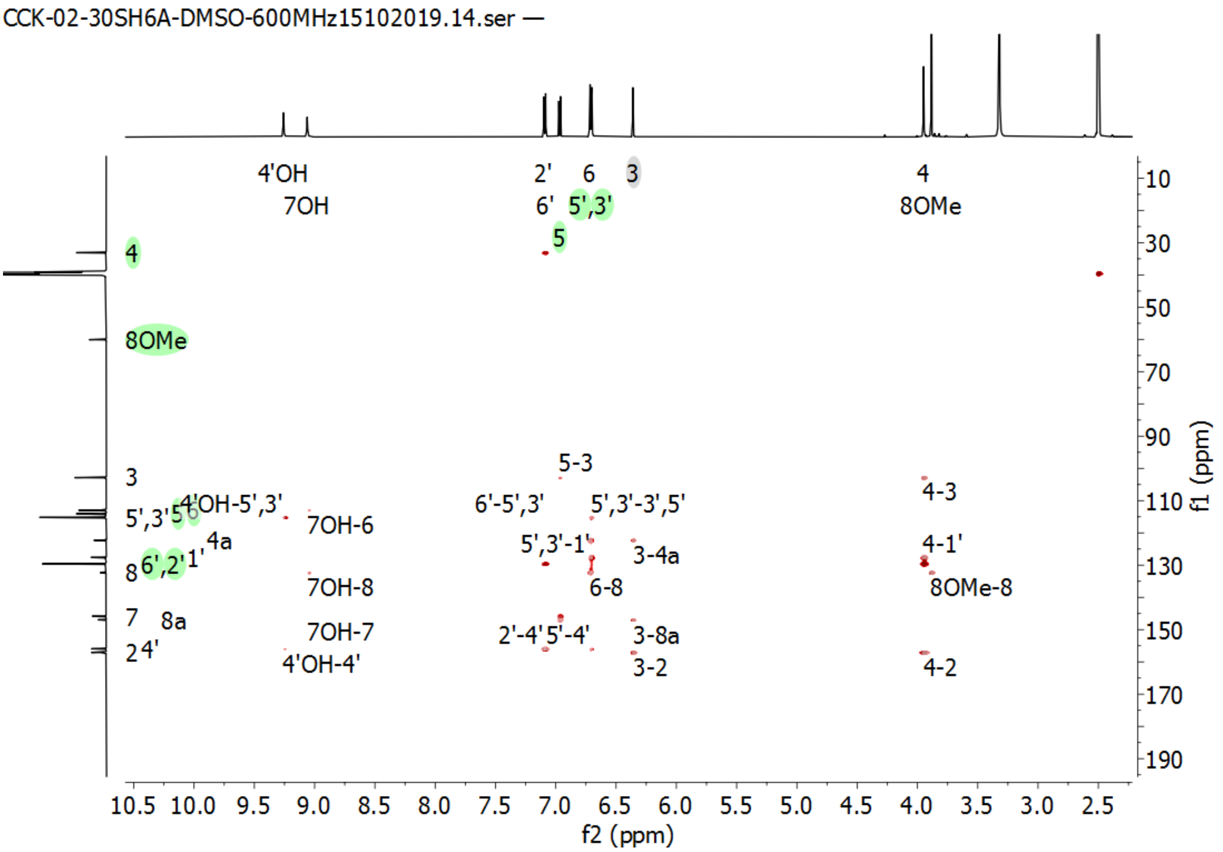

Figure S80. HMBC (600 MHz, DMSO, 25°C) spectrum of usambarin J (**9**)

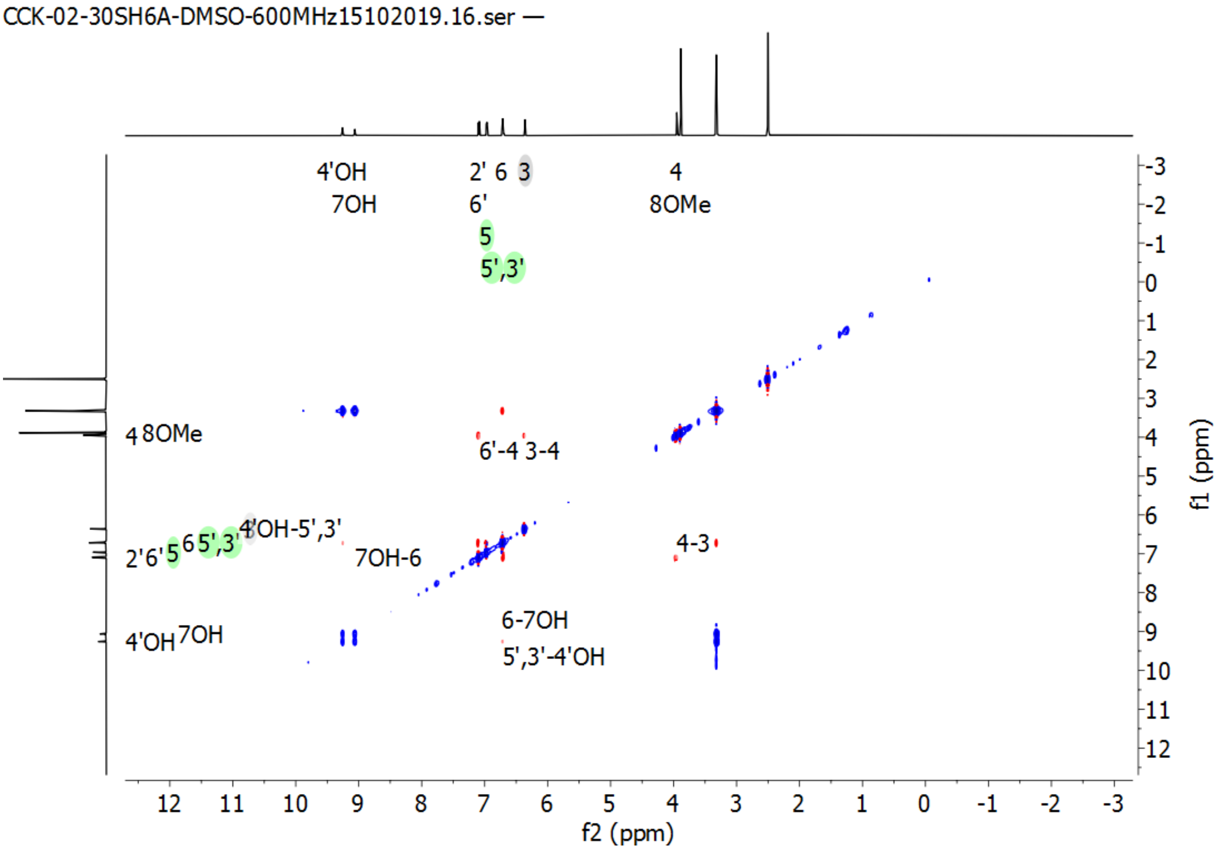

Figure S81. NOESY (600 MHz, DMSO, 25°C) spectrum of usambarin J (**9**)

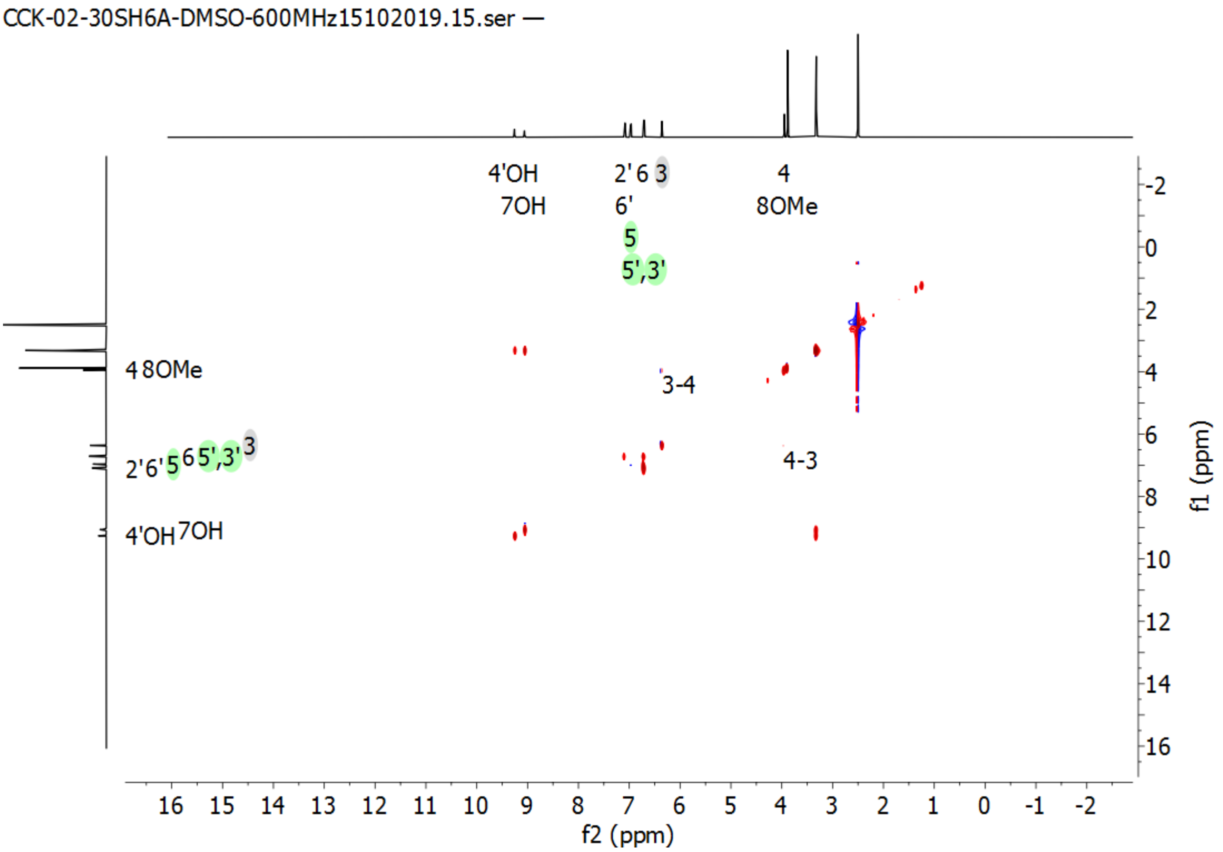

Figure S82. TOCSY (600 MHz, DMSO, 25°C) spectrum of usambarin J (**9**)

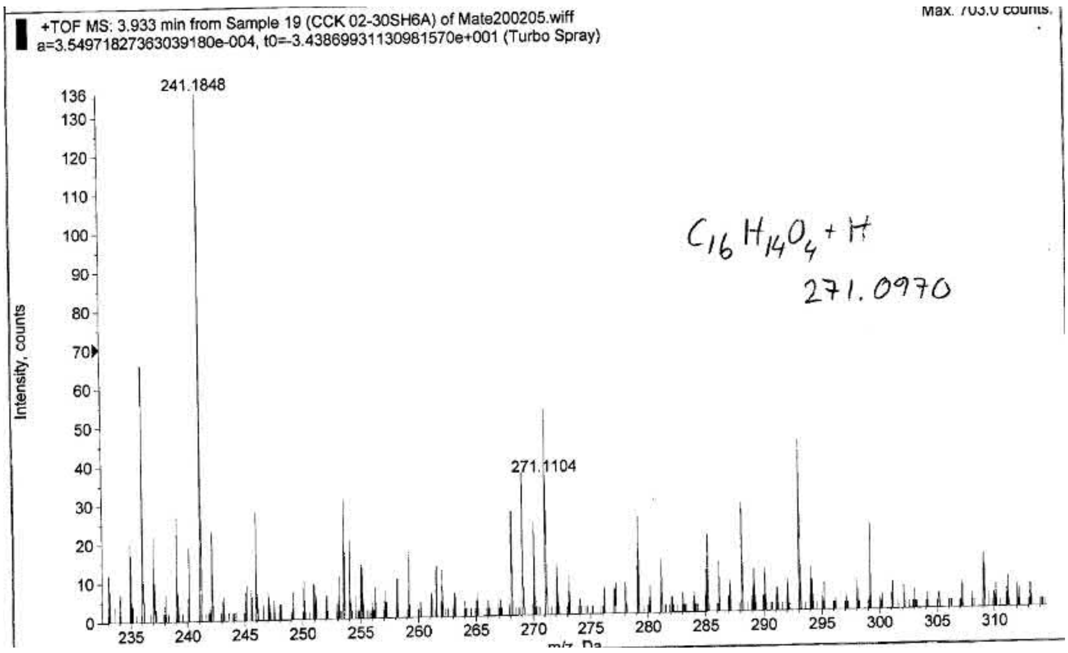

Figure S83. HRMS spectrum of usambarin J (**9**)

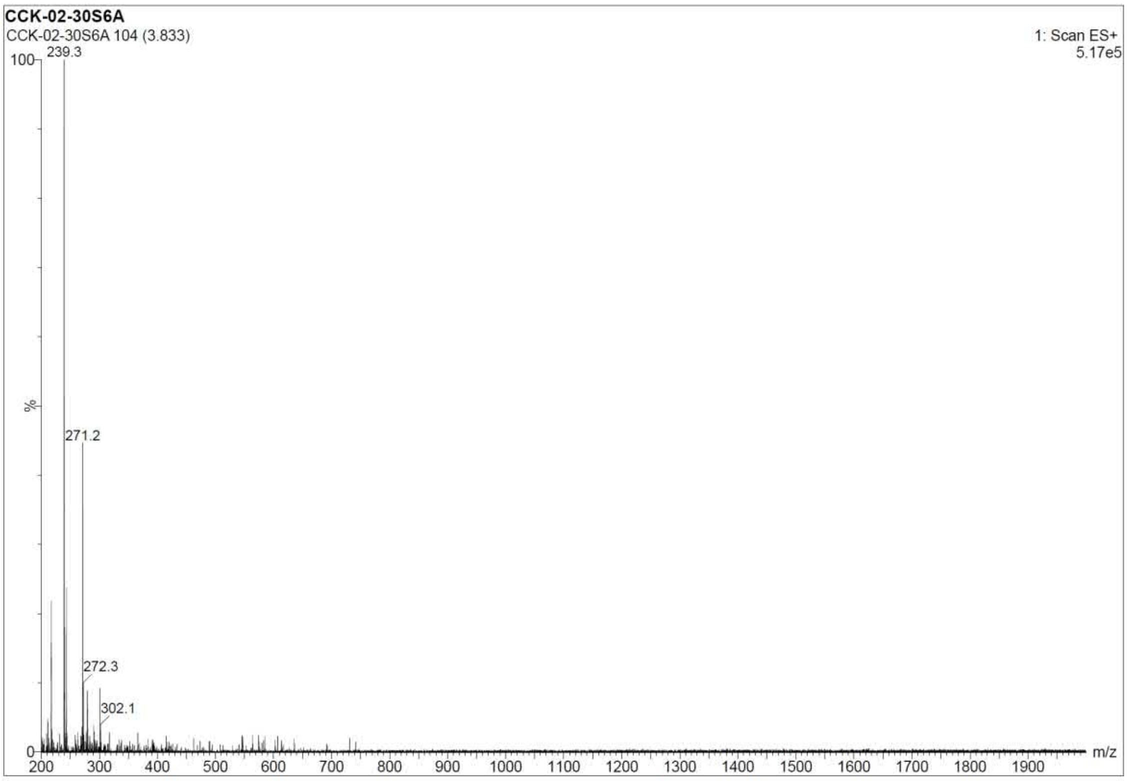

Figure S84.ESI-MS spectrum of usambarin J (**9**)

Spectroscopic data of Usambarin K (10)

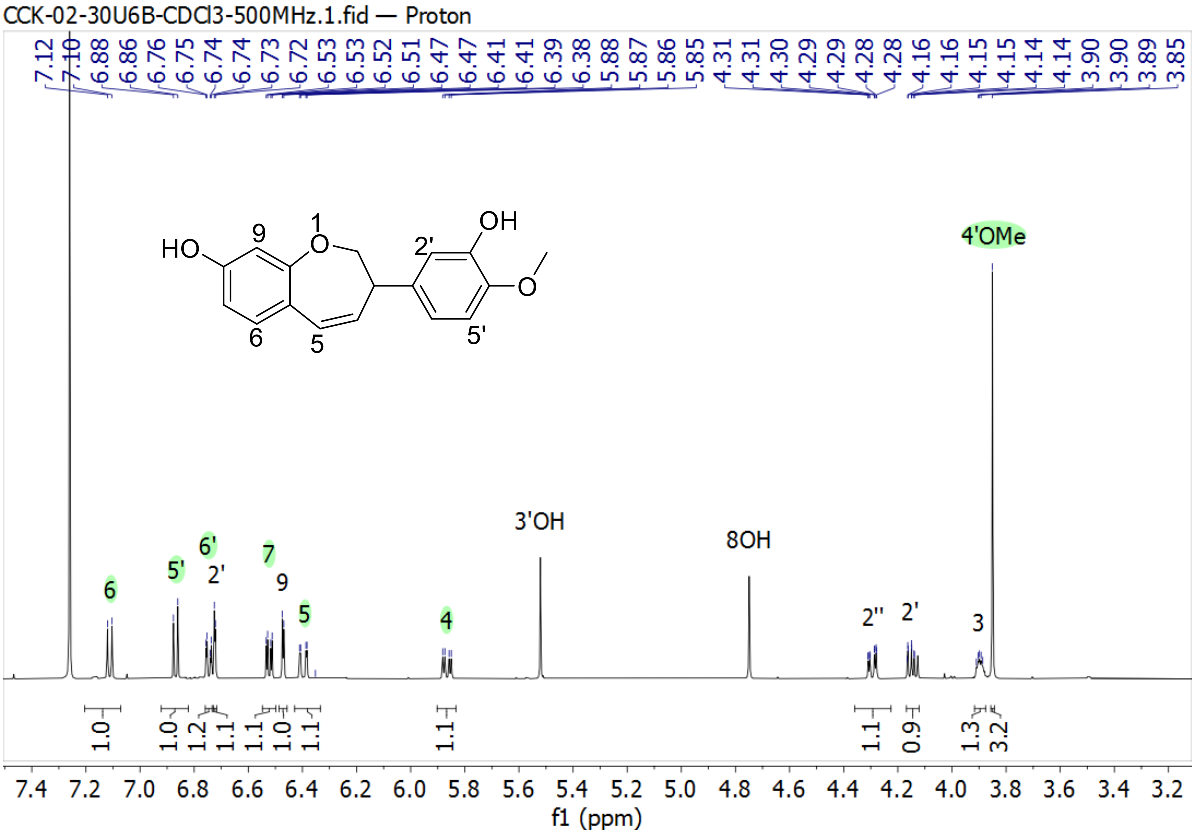

Figure S85. ^{1}H NMR (500 MHz, $CDCl_3$, 25°C) spectrum of usambarin K (**10**)

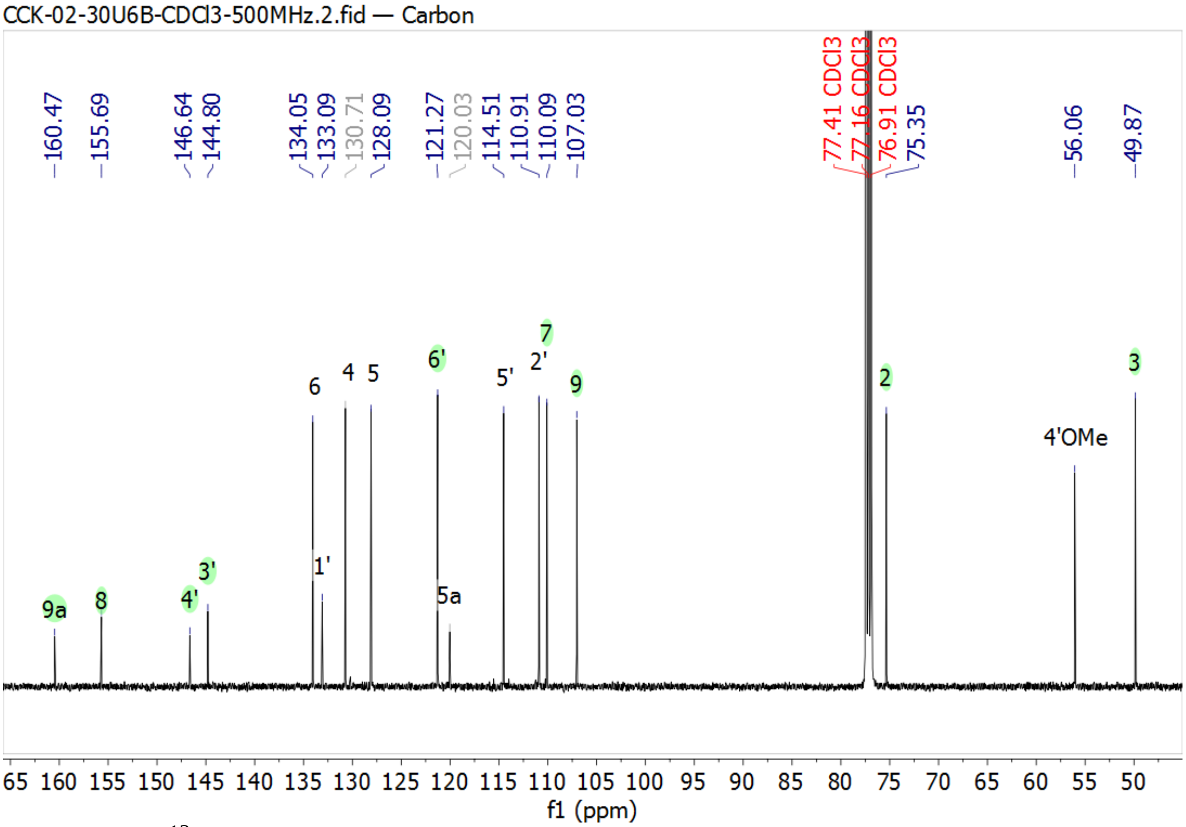

Figure S 86. ^{13}C NMR (500 MHz, $CDCl_3$, 25°C) spectrum of usambarin K (**10**)

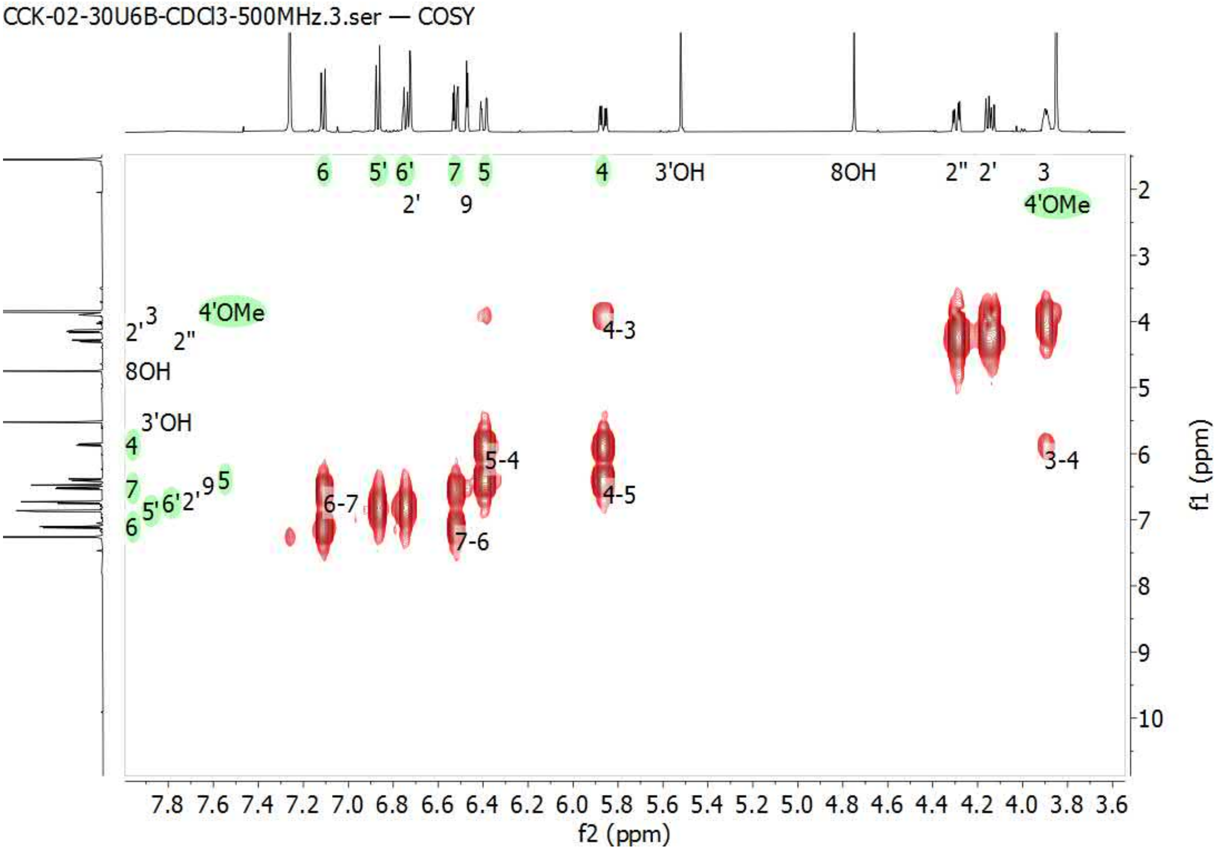

Figure S87. COSY (500 MHz, $CDCl_3$, 25°C) spectrum of usambarin K (**10**)

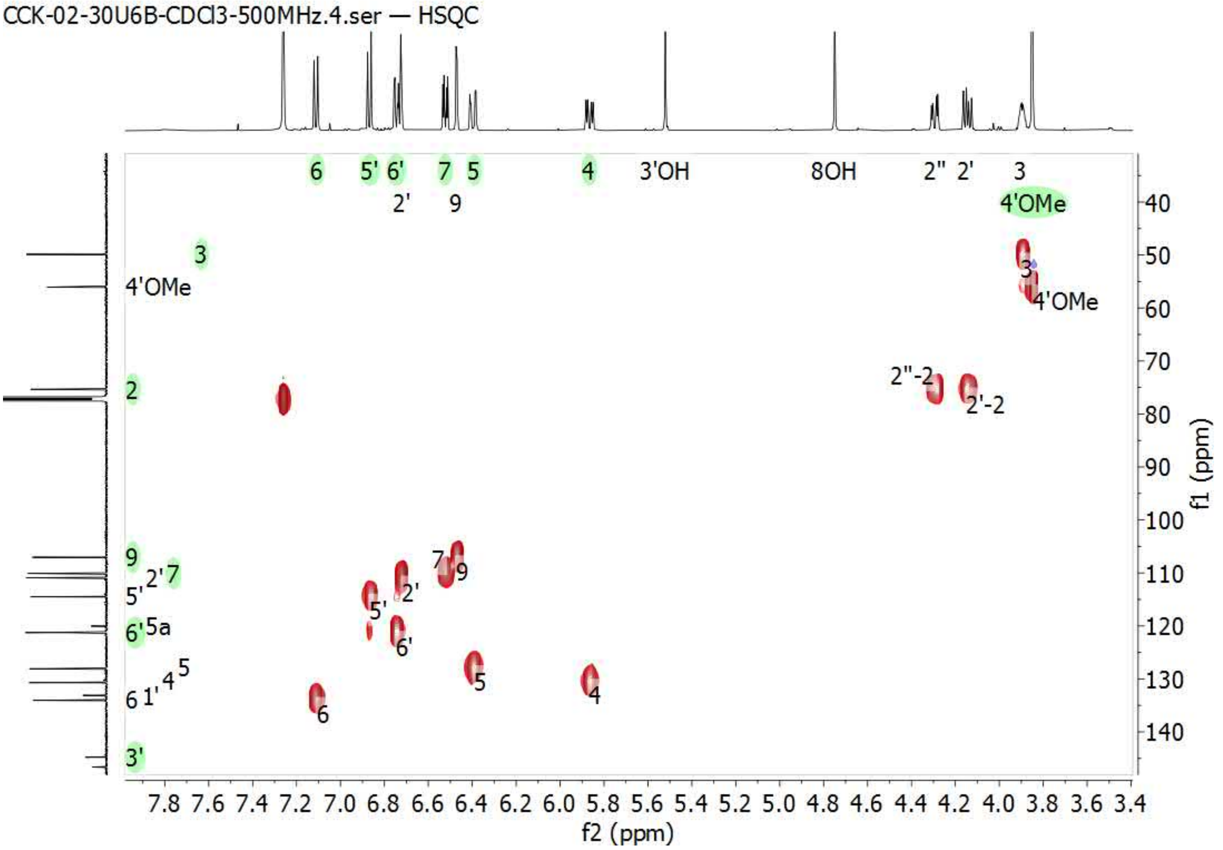

Figure S88. HSQC (500 MHz, $CDCl_3$, 25°C) spectrum of usambarin K (**10**)

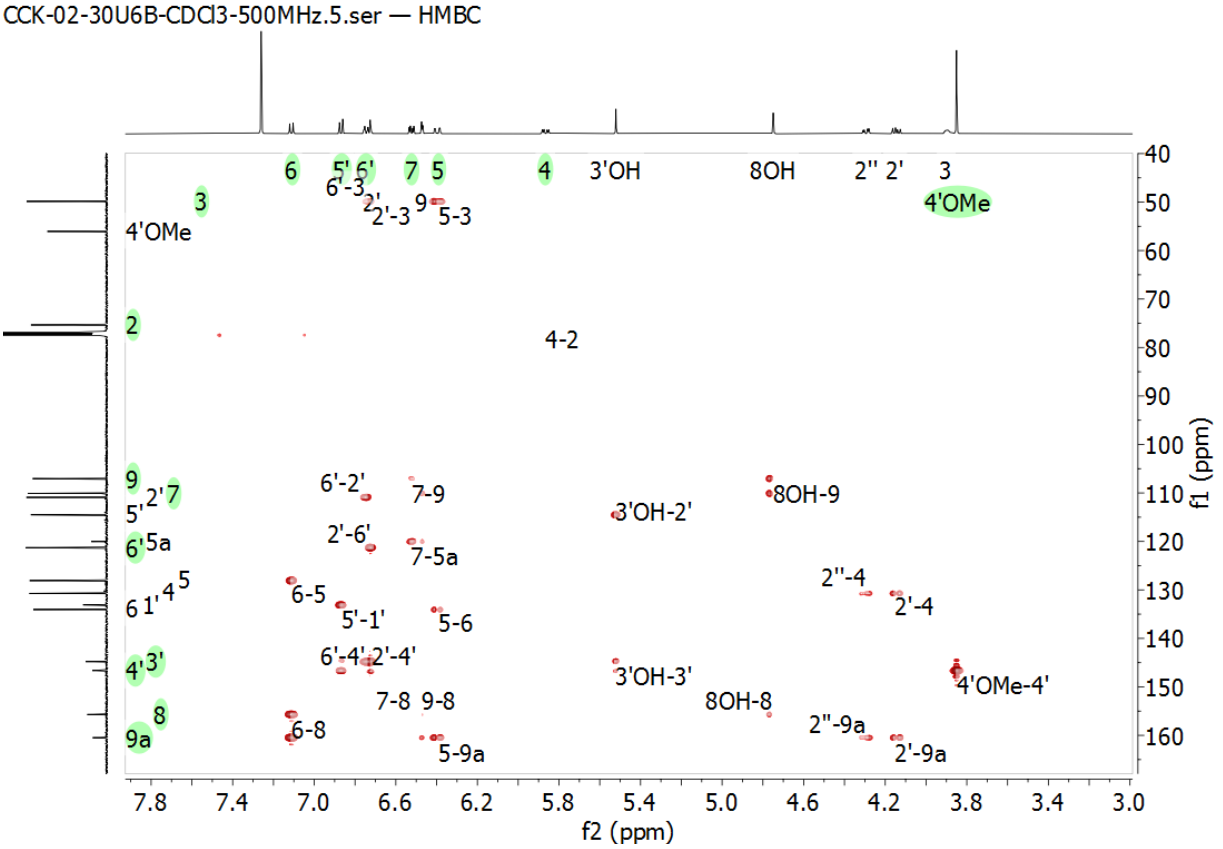

Figure S89. HMBC (500 MHz, $CDCl_3$, 25°C) spectrum of usambarin K (**10**)

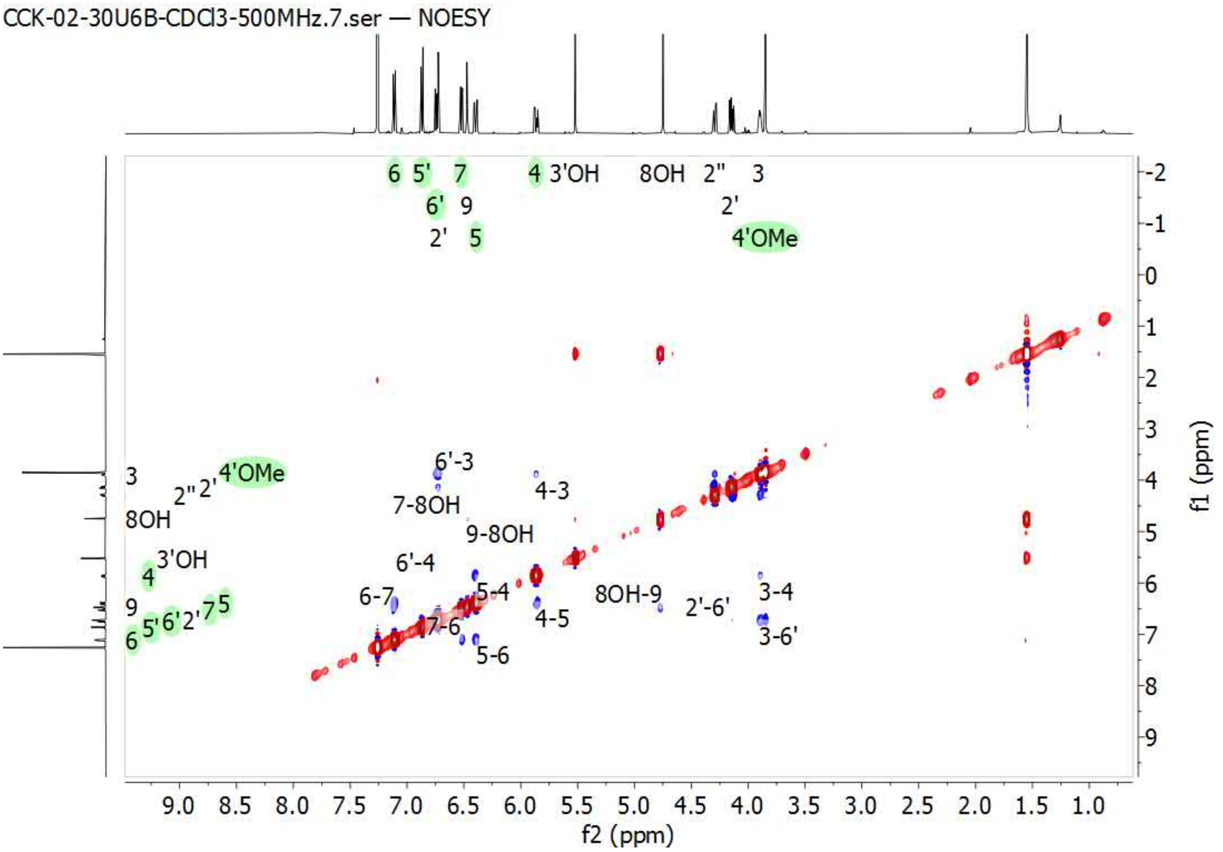

Figure S90. NOESY (500 MHz, $CDCl_3$, 25°C) spectrum of usambarin K (**10**)

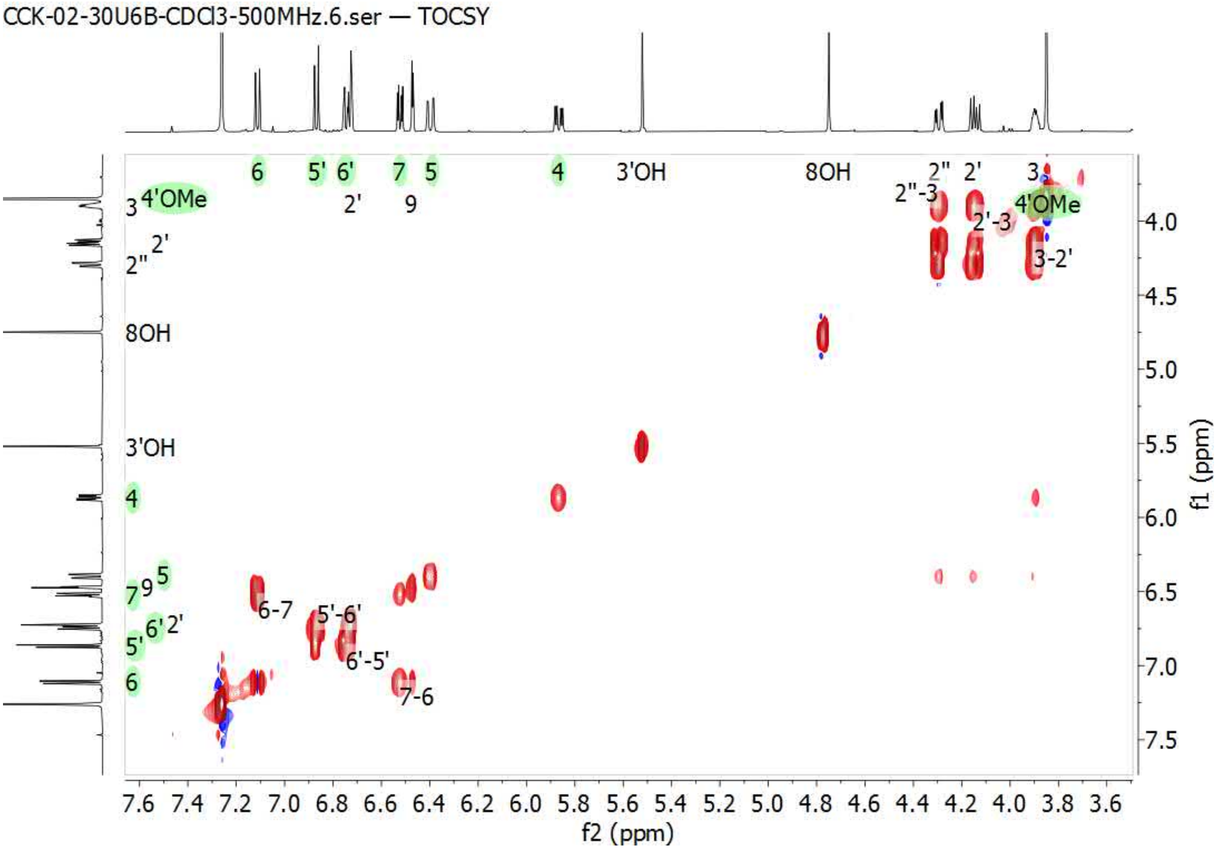

Figure S91. TOCSY (500 MHz, $CDCl_3$, 25°C) spectrum of usambarin K (**10**)

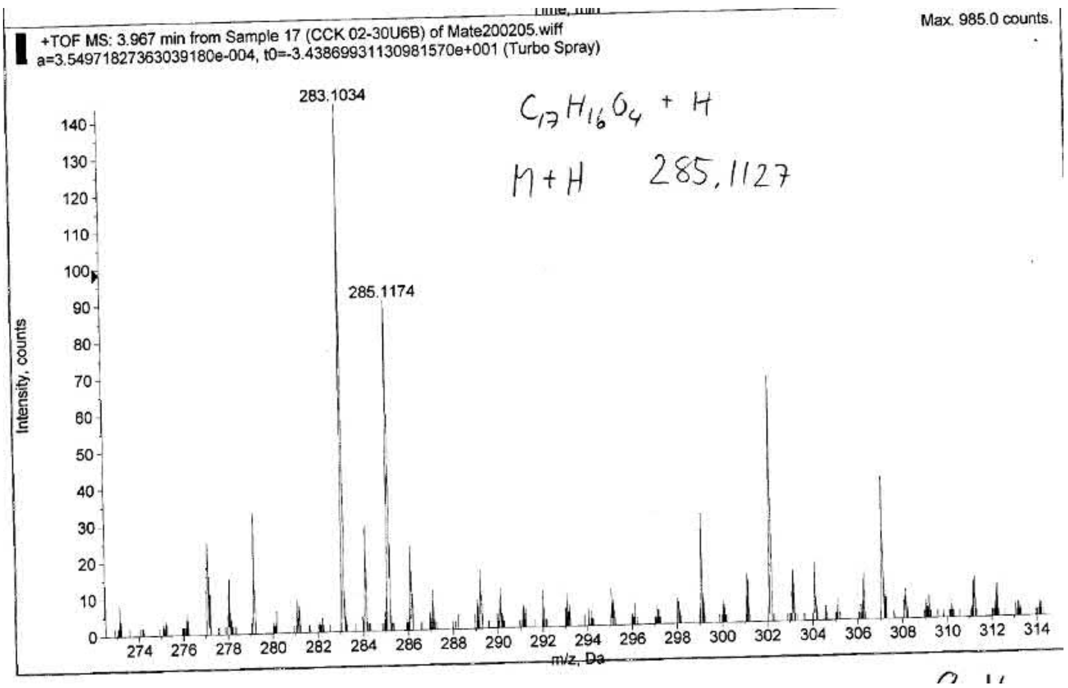

Figure S92. HRMS spectrum of usambarin K (**10**)

Physical and spectroscopic data

Compound **1**, white crystal ($CDCl_3$); UV (MeOH)) λ_{max} (log ε) 270 nm (4.0), 310 nm (4.1); ^{1}H NMR (DMSO-d_6, 600 MHz) δ_H 9.35 (s, 8-OH), 8.00 (1H, *d*, *J* = 8.9 Hz, H-4), 7.89 (1H, *d*, *J* = 8.4 Hz, H-6), 7.76 (1H, *d*, *J* = 8.4 Hz, H-5), 7.59 (1H, *d*, *J* = 2.6 Hz, H-1), 7.39 (1H, s, H-7), 7.23 (1H, *dd*, *J* = 8.9, 2.6 Hz, H-3), 5.35 (1H, m, H-2'), 3.98 (3H, s, 2-OMe), 3.83 (3H, s, 9-OMe), 3.69 (1H, *d*, *J* = 7.5 Hz, H-1'), 1.96 (3H, m, H-4'), 1.69 (1H, *d*, *J* = 1.6 Hz, H-5'); ^{13}C NMR (DMSO-d_6, 150 MHz) δ_C 157.9 (C, C-2), 150.7 (C, C-11a), 147.8 (C, C-10a), 147.3 (C, C-8), 145.5 (C, C-9), 131.5 (C, C-3'), 127.3 (C, C-4a), 122.8 (CH, C-5), 122.1 (CH, C-2'), 119.8 (C, C-6a), 119.3 (C, C-6b), 118.9 (C, C-10), 103.9 (CH, C-7), 98.7 (CH, C-1), 60.5 (CH_3, 9-OMe), 55.3 (CH_3, 2-OMe), 24.9 (CH_3, C-5'), 23.3 (CH_2, C-1'), 17.7 (CH_3, C-4'); HREIMS $[M+1]^+$ m/z 363.1396 (calcd for $C_{23}H_{22}O_4$, 363.1391).

Compound **2**, white solid; UV (MeOH) λ_{max} (log ε) 273 nm (4.0), 321 nm (4.0); ^{1}H NMR (Methanol-d_4, 500 MHz) δ 7.84 (1H, d, *J* = 8.8 Hz, H-4), 7.68 (1H, d, *J* = 8.4 Hz, H-6), 7.63 (1H, d, *J* = 8.5 Hz, H-5), 7.59 (1H, d, *J* = 2.4 Hz, H-1), 7.29 (1H, s, H-7), 7.10 (1H, dd, *J* = 8.8, 2.5 Hz, H-3), 5.44 (1H, *m*, H-2'), 3.89 (3H, s, 9-OMe), 3.75 (2H, d, *J* = 7.4 Hz, H-1'), 1.99 (3H, d, *J* = 1.4 Hz, H-5'), 1.73 (3H, d, *J* = 1.5 Hz, H-4');^{13}C NMR (Methanol-d_4, 125 MHz) δ 157.3

(C, C-2), 152.7 (C, C-11a), 150.0 (C, C-10a), 148.3 (C, C-8), 146.7 (C, C-9), 133.1 (C, C-3'), 131.3 (C, C-4), 128.8 (C, C-4a), 124.0 (C, C-6a), 123.9 (CH, C-5), 123.4 (CH, C-2'), 121.6 (C, C-11b), 121.1 (C, C-6b), 120.6 (C, C-10), 116.0 (CH, C-6), 104.4 (CH, C-7), 103.2 (CH, C-1), 61.6 (CH_3, 9-OMe), 26.0 (CH_3, C-4'), 24.6 (CH_2, C-1'), 18.2 (CH_3, C-5'); HREIMS $[M+1]^+$ m/z 349.1440 (calcd for $C_{22}H_{20}O_4$, 349.1434).

Compound **3**, white amorphous solid; UV (MeOH) λ_{max} (log ε) 270 nm (4.0), 350 nm (4.1); ^{1}H NMR (Chloroform-*d*, 500 MHz) δ 7.85 (1H, d, *J* = 8.9 Hz, H-4), 7.73 (1H, d, *J* = 8.3 Hz, H-6), 7.67 (1H, s, H-1), 7.64 (1H, d, *J* = 3.1 Hz, H-5), 7.36 (1H, s, H-7), 7.16 (1H, dd, *J* = 9.0, 2.6 Hz, H-3), 7.06 (1H, d, *J* = 9.8 Hz, H-4'), 5.81 (1H, d, *J* = 9.9 Hz, H-3'), 5.45 (1H, s, 8-OH), 4.03 (3H, s, 2-OMe), 1.56 (6H, d, *J* = 2.0 Hz, 2'Me, 2'Me_2); ^{13}C NMR (Chloroform-*d*, 125 MHz) δ 158.3 (C, C-2), 151.6 (C, C-11a), 146.1 (C, C-10a), 141.8 (C, C-9), 138.7 (C, C-8), 130.8 (C, C-3'), 130.2 (C, C-4), 127.8 (C, C-6a), 123.0 (CH, C-5), 122.4 (C, C-11b), 120.5 (C, C-6b), 118.2 (CH, C-3), 117.8 (C, C-4a), 116.7 (CH, C-4'), 115.8 (CH, C-6), 106.8 (C, C-10), 104.6 (CH, C-7), 78.0 (C, C-2'), 55.7 (CH_3, 2-OMe), 27.9 ($(CH_3)_2$, C-2' $(Me)_2$); HREIMS $[M+1]^+$ m/z 347.1283 (calcd for $C_{22}H_{18}O_4$, 347.1278).

Compound **4**, white crystal; UV (MeOH) λ_{max} (log ε) 230 nm (3.9), 255 nm (3.9); ^{1}H NMR (Chloroform-*d*, 500 MHz) δ_H 7.78 (1H, dd, *J* = 8.5, 3.4 Hz, H-4), 7.78 (1H, dd, *J* = 8.5, 3.4 Hz, H-5), 7.59 (1H, s, H-8), 7.30 (1H, dd, *J* = 8.5, 1.8 Hz, H-6), 7.16 (1H, d, *J* = 2.5 Hz, H-1), 7.11 (1H, dd, *J* = 8.8, 2.4 Hz, H-3), 6.90 (1H, d, *J* = 8.3 Hz, H-5'), 6.87 (1H, d, *J* = 8.3 Hz, H-6'), 6.40 (1H, d, *J* = 10.0 Hz, H-4''), 5.61 (1H, d, *J* = 10.1 Hz, H-3''), 5.55 (1H, s, 4'OH), 5.13 (1H, s, 2-OH), 1.52 (6H, s, 2''Me, 2''Me_2); ^{13}C NMR (Chloroform-*d*, 125 MHz) δ_C 153.9 (C, C-2), 144.2 (C, C-4'), 139.6 (C, C-3'), 138.1 (C, C-7), 134.6 (C, C-8a), 131.5 (C, C-1'), 130.5 (CH, C-3''), 129.7 (CH, C-4), 127.9 (C, C-4a), 127.6 (CH, C-5), 126.9 (C, C-8), 126.2 (CH, C-6), 122.4 (CH, C-5'), 121.1 (CH, C-4''), 119.2 (C, C-2'), 117.9 (CH, C-3), 114.6 (CH, C-6'), 109.7 (CH, C-1), 76.5 (C, C-2''), 27.9 ($(CH_3)_2$, C-2''-$(Me)_2$); HREIMS $[M+1]^+$ m/z 319.1334 (calcd for $C_{21}H_{18}O_3$, 319.1329).

Compound **5**, white solid; UV(MeOH) λ_{max} (log ε) 234 nm (3.9), 262 nm 3.9), 290sh nm (4.0); ^{1}H NMR (Chloroform-*d*, 500 MHz) δ_H 7.76 (1H, *d*, *J* = 3.0 Hz, H-5), 7.76 (1H, *d*, *J* = 8.6 Hz, H-4), 7.55 (1H, *s*, H-8), 7.24 (1H, d, *J* = 1.8 Hz, H-6), 7.14 (1H, *d*, *J* = 2.5 Hz, H-1), 7.10 (1H, *dd*, *J* = 8.8, 2.6 Hz, H-3), 6.86 (1H, *d*, *J* = 8.2 Hz, H-6'), 6.83 (1H, *d*, *J* = 8.2 Hz, H-5'), 5.55

(1H, *s*, 3'-OH), 5.43 (1H, *s*, 4'-OH), 5.29 (1H, *m*, H-2''), 5.00 (1H, *s*, 2-OH), 3.36 (2H, *d*, *J* = 6.8 Hz, H-1''), 1.76 (3H, *s*, H-5''), 1.68 (3H, *s*, H-4''); ^{13}C NMR (Chloroform-*d*, 126 MHz) δ_C 153.8 (C, C-2), 144.0 (C, C-4'), 142.4 (C, C-3'), 139.9 (C, C-4a), 135.7 (C, C-3''), 134.9 (C, C-1'), 134.6 (C, C-7), 129.8 (CH, C-4), 127.7 (CH, C-8a), 127.5 (C, C-5) 126.8 (CH, C-8), 126.2 (CH, C-6) 125.4 (C, C-2'), 122.8 (CH, C-5'), 122.2 (CH, C-2''), 117.8 (CH, C-3), 112.9 (CH, C-6'), 109.7 (CH, C-1), 27.7 (CH_2, C-1''), 25.9 (CH_3, C-4''), 18.1 (CH_3, C-5''); EIMS $[M-1]^+$ m/z 319.2 (calcd for $C_{21}H_{20}O_3$, 321.1412).

Compound **6**, white solid; UV(MeOH), λ_{max} (log ε) 234 nm (3.9); ^{1}H NMR (Chloroform-*d*, 500 MHz) δ_H 7.78 (1H, *dd*, *J* = 8.5, 2.6 Hz, H-4), 7.78 (1H, *dd*, *J* = 7.4, 2.6 Hz, H-5), 7.55 (1H, *m*, H-8), 7.25 (1H, *m*, H-6), 7.12 (1H, *d*, *J* = 2.5 Hz, H-1), 7.10 (1H, *dd*, *J* = 8.7, 2.6 Hz, H-3), 6.97 (1H, *d*, *J* = 8.3 Hz, H-6'), 6.90 (1H, *d*, *J* = 8.2 Hz, H-5'), 5.58 (1H, *s*, 2-OH), 5.30 (1H, *s*, 4'-OH), 5.08 (1H, *m*, H-2''), 3.86 (3H, *s*, 3'-OMe), 3.33 (2H, *dt*, *J* = 6.6, 1.3 Hz, H-1''), 1.58 (3H, *d*, *J* = 1.4 Hz, H-4''), 1.35 (3H, *d*, *J* = 1.4 Hz, H-5''); ^{13}C NMR (Chloroform-*d*, 126 MHz) δ_C 153.8 (C, C-2), 148.5 (C, C-4'), 145.6 (C, C-3'), 139.9 (C, C-7), 135.7 (C, C-1'), 134.6 (C, C-4a), 133.3 (C, C-2'), 131.5 (C, C-3''), 129.7 (CH, C-4), 127.8 (C, C-8a), 127.4 (CH, C-5), 126.9 (CH, C-8), 126.9 (CH, C-6'), 123.5 (CH, C-2''), 117.7 (CH, C-3), 113.3 (CH, C-5'), 109.7 (CH, C-1), 61.4 (C, OMe-3'), 26.9 (CH_2, C-1''), 25.8 (CH_3, C-4''), 17.8 (CH_3, C-5''); HREIMS $[M+1]^+$ m/z 335.1647 (calcd for $C_{22}H_{22}O_3$, 335.1569).

Compound **7**, white solid; UV(MeOH), λ_{max} (log ε) 231 nm (3.9), 257 nm (3.9); ^{1}H NMR (Chloroform-*d*, 500 MHz) δ_H 8.04 (1H, *s*, H-8), 7.81 (1H, *d*, *J* = 8.5 Hz, H-5), 7.72 (1H, *d*, *J* = 8.9 Hz, H-4), 7.52 (1H, *dd*, *J* = 8.5, 1.7 Hz, H-6), 7.25 (1H, *m*, H-3), 7.25 (1H, *m*, H-2'), 7.17 (1H, *dd*, *J* = 8.2, 2.1 Hz, H-6'), 6.98 (1H, *d*, *J* = 8.2 Hz, H-5'), 5.25 (1H, *m*, 3'-OH), 5.23 (1H, *m*, H-2''), 5.21 (1H, *m*, 4'-OH), 3.95 (3H, *s*, 2-OMe), 3.83 (2H, *d*, *J* = 6.8 Hz, H-1''), 1.91 (3H, *d*, *J* = 1.3 Hz ,H- 4''), 1.69 (3H, *d*, *J* = 1.5 Hz, H-5''); ^{13}C NMR (Chloroform-*d*, 125 MHz) δ_C 154.7 (C, C-2), 143.9 (C, C-4'), 143.2 (C, C-3'), 138.3 (C, C-7), 135.5 (C, C-1'), 133.4 (C, C-4a), 133.4, (C, C-8a), 131.6 (C, C-3''), 129.1 (CH, C-5), 128.5 (C, C-1), 127.4 (CH, C-4), 123.5 (CH, C-2''), 123.0 (CH, C-6), 121.4 (CH, C-8), 120.4 (CH, C-6'), 115.9 (CH, C-5'), 114.8 (CH, C-2'), 113.8 (CH, C-3), 57.0 (C, 2-OMe), 25.9 (CH_3, C-5''), 24.3 (CH_2, C-1''), 18.3 (CH_3, C-4''); HREIMS $[M+1]^+$ m/z 335.1647 (calcd for $C_{22}H_{22}O_3$, 335.1569).

Compound **8**, white solid; ^{1}H NMR (500 MHz, Chloroform-*d*) δ_H 7.78 (1H, *dd*, *J* = 8.5, 2.3 Hz, H-4), 7.78 (1H, *dd*, *J* = 8.5, 2.3 Hz, H-5), 7.45 (1H, *d*, *J* = 1.6 Hz, H-8), 7.13 (1H, *d*, *J* = 9.5 Hz,

H-3), 7.11 (1H, *d*, *J* = 5.8 Hz, H-6), 7.10 (1H, *d*, *J* = 2.0 Hz, H-1), 6.75 (1H, *s*, H-6'), 5.60 (1H, *s*, 4'-OH), 5.12 (1H, *m*, H- 2''), 4.94 (1H, *s*, 2-OH), 2.96 (1H, *d*, *J* = 7.4 Hz, H-1''), 2.32 (1H, *dt*, *J* = 9.0, 6.7 Hz, H-4'''), 1.68 (1H, *t*, *J* = 6.8 Hz, H-3'''), 1.62 (1H, *d*, *J* = 1.6 Hz, H-5''), 1.34 (1H, *s*, H-2'''-$(Me)_2$), 1.33 (1H, s, H-4''); ^{13}C NMR (Chloroform-*d*, 125 MHz) δ_C 153.7 (C, C-2), 144.5 (C, C-4'), 139.0 (C, C-3'), 138.5 (C, C-7), 134.8 (C, C-4a), 132.6 (C, C-1'), 131.3 (C, C-5'), 129.8 (CH, C-4), 127.9 (C, C-8a), 127.7 (CH, C-5), 127.2 (CH, C-8), 126.5 (CH, C-6), 123.9 (CH, C-2''), 119.7 (C, C-2'), 117.7 (CH, C-3) 112.4 (CH, C-6'), 109.6 (CH, C-1), 74.8 (C, C-2'''), 33.2 (CH_2, C-3'''), 32.0 (CH_2, C-1''), 27.0 ($(CH_3)_2$, C-2'''-$(Me)_2$), 25.9 (CH_3, C-5''), 21.9 (CH_2, C-4'''), 17.7 (CH_3, C-4''); HREIMS $[M+1]^+$ m/z 389.2117 (calcd for $C_{26}H2_8O_3$, 389.2038).

Compound **9**, white solid; ^{1}H NMR (DMSO-d_6, 600 MHz) δ_H 9.26 (1H, *s*, 4'-OH), 9.06 (1H, *s*, 7-OH), 7.10 (2H, *d*, *J* = 2.0 Hz, H-2'/6'), 6.97 (1H, *d*, *J* = 8.3 Hz, H-5), 6.72 (1H, *d*, *J* = 8.3 Hz, H-6), 6.71 (2H, *dd*, *J* = 3.9, 2.5 Hz, H-3'/5'), 6.35 (1H, *d*, *J* = 1.0 Hz, H-3), 3.95 (2H, *s*, H-4), 3.89 (3H, *s*, 8-OMe); ^{13}C NMR (DMSO-d_6, 150 MHz) δ 157.1 (C, C-2), 155.9 (C, C-4'), 147.0 (C, C-8a), 145.9 (C, C-7), 132.4 (C, C-8), 129.6 (CH, C-2', 6'), 127.6 (C, C-1'), 122.3 (C, C-4a), 114.1 (CH, C-5), 113.0 (CH, C-6), 102.9 (CH, C-3), 60.1 (CH_3, 8-OMe), 33.1 (CH_2, C-4); HREIMS $[M+1]^+$ m/z 271.0970 (calcd for $C_{16}H_{14}O_4$, 271.0892).

Compound **10**, white solid; ^{1}H NMR (Chloroform-*d*, 500 MHz) δ_H 7.11 (1H, *d*, *J* = 8.3 Hz, H-6), 6.87 (1H, *d*, *J* = 8.0 Hz, H-5'), 6.75 (1H, *dd*, *J* = 8.0, 2.0 Hz, H-6'), 6.72 (1H, d, *J* = 2.0 Hz, H-2'), 6.52 (1H, *dd*, *J* = 8.4, 2.6 Hz, H-7), 6.47 (1H, *d*, *J* = 2.6 Hz, H-9), 6.40 (1H, *dd*, *J* = 11.8, 2.0 Hz, H-5), 5.87 (1H, *dd*, *J* = 11.8, 4.0 Hz, H-4), 4.29 (1H, *ddd*, *J* = 11.7, 3.2, 1.0 Hz, H-2^{α}), 4.14(1H, *d*, *J* = 11.8, 6.7 Hz, H-2^{β}), 3.90 (1H, *dd*, *J* = 5.5, 3.2 Hz, H-3), 3.85 (3H, *s*, 4'-OMe); ^{13}C NMR (Chloroform-*d*, 125 MHz) δ 160.5 (C, C-9a), 155.7 (C, C-8), 146.6 (C, C-3'), 144.8 (C, C-4'), 134.1(CH, C-6), 133.1 (C, C-1'), 130.7 (CH, C-4), 128.1 (CH, C-5), 121.3 (CH, C-6'), 114.5 (CH, C-5'), 110.9 (C, C-2'), 110.1 (CH, C-7), 107.0 (CH, C-9), 75.4 (CH_2, C-2), 56.1 (CH_3, 4'-OMe), 49.9 (CH, C-3); HREIMS $[M+1]^+$ m/z 285.1127 (calcd for $C_{17}H_{16}O_4$, 285.1049).

Compound **11**, white crystal; UV (MeOH) λ_{max} (log *ε*) 234 nm (3.7); ^{1}H NMR (Chloroform-*d*, 500 MHz) δ_H 7.80 (1H, *d*, *J* = 9.6 Hz, H-4), 7.70 (1H, *d*, *J* = 2.3 Hz, H-2'), 7.68 (1H, *s*, H-5), 7.48 (1H, *s*, H-8), 6.83 (1H, *dd*, *J* = 2.3, 1.0 Hz, H-3'), 6.38 (1H, *d*, *J* = 9.6 Hz, H-3); ^{13}C NMR

(Chloroform-*d*, 125 MHz) δ_C 161.2 (C, C-2), 156.6 (C, C-7), 152.2 (C, C-8a), 147.1 (CH, C-2'), 144.2 (CH, C-4), 125.0 (C, C-6), 120.0 (CH, C-5), 115.6 (C-4a), 106.5 (CH, C-3'), 100.0 (CH, C-8); EIMS $[M+1]^+$ m/z 186.04;

Compound **12**, white crystal; UV (MeOH) λ_{max} (log ε) 234 nm (3.6); ; ^{1}H NMR (Chloroform-*d*, 500 MHz) δ_H 8.16 (1H, *d*, *J* = 9.8 Hz, H-4), 7.59 (1H, *d*, *J* = 2.4 Hz, H-2'), 7.14 (1H, *s*, H-8), 7.02 (1H, *d*, *J* = 2.0 Hz, H-3'), 6.28 (1H, *d*, *J* = 9.7 Hz, H-3), 4.27 (3H, *s*, 5-OMe); ^{13}C NMR (Chloroform-*d*, 126 MHz) δ_C 161.7 (C, C-2), 158.9 (C, C-7), 153.1 (C, C-8a), 150.0 (C, C-5) 145.2 (CH, C-2'), 139.7 (CH, C-4), 113.0 (CH, C-3), 113.0 (C, C-6), 106.8 (C, C-4a), 105.4 (CH, C-3'), 94.3 (CH, C-8), 60.5 (CH_3, C-5-OMe); EIMS $[M+1]^+$ m/z 216.04.

Single Crystal X-ray Diffraction Analysis

Table S1. Crystal data and structure refinement of usambarin A (1).

Identification code	ag_ag_536_0m_a	
Empirical formula	C23 H22 O4	
Formula weight	362.40	
Temperature	150(2) K	
Wavelength	0.71073 Å	
Crystal system	Monoclinic	
Space group	P 1 21/c 1	
Unit cell dimensions	a = 19.86(5) Å	α= 90°.
	b = 4.565(11) Å	β= 111.26(4)°.
	c = 20.96(5) Å	γ = 90°.
Volume	1771(7) $Å^3$	
Z	4	
Density (calculated)	1.359 Mg/m^3	
Absorption coefficient	0.092 mm^{-1}	
F(000)	768	
Crystal size	0.22 x 0.16 x 0.06 mm^3	
Theta range for data collection	1.973 to 24.998°.	
Index ranges	-23<=h<=23, -5<=k<=5, -24<=l<=24	
Reflections collected	15879	
Independent reflections	3131 [R(int) = 0.2298]	
Completeness to theta = 24.998°	100.0 %	
Absorption correction	Semi-empirical from equivalents	
Max. and min. transmission	0.7454 and 0.4267	
Refinement method	Full-matrix least-squares on F^2	
Data / restraints / parameters	3131 / 0 / 249	
Goodness-of-fit on F^2	0.978	
Final R indices [I>2sigma(I)]	R1 = 0.0753, wR2 = 0.1536	
R indices (all data)	R1 = 0.1775, wR2 = 0.1948	
Extinction coefficient	n/a	
Largest diff. peak and hole	0.282 and -0.285 e.$Å^{-3}$	

Table S2. Crystal data and structure refinement of usambarin D (4)

Identification code	ag_ag_526_0m_a	
Empirical formula	C21 H18 O3	
Formula weight	318.35	
Temperature	150(2) K	
Wavelength	0.71073 Å	
Crystal system	Triclinic	
Space group	P-1	
Unit cell dimensions	a = 8.6631(7) Å	α= 62.2430(10)°.
	b = 9.8115(8) Å	β= 88.7730(10)°.
	c = 10.5083(9) Å	γ = 88.7780(10)°.
Volume	790.16(11) Å^3	
Z	2	
Density (calculated)	1.338 Mg/m^3	
Absorption coefficient	0.089 mm^{-1}	
F(000)	336	
Crystal size	0.38 x 0.26 x 0.16 mm^3	
Theta range for data collection	2.190 to 30.812°.	
Index ranges	-12<=h<=12, -14<=k<=14, -15<=l<=15	
Reflections collected	24545	
Independent reflections	4921 [R(int) = 0.0302]	
Completeness to theta = 25.242°	99.9 %	
Absorption correction	Semi-empirical from equivalents	
Max. and min. transmission	0.7461 and 0.7196	
Refinement method	Full-matrix least-squares on F^2	
Data / restraints / parameters	4921 / 0 / 219	
Goodness-of-fit on F^2	1.039	
Final R indices [I>2sigma(I)]	R1 = 0.0494, wR2 = 0.1345	
R indices (all data)	R1 = 0.0645, wR2 = 0.1453	
Extinction coefficient	n/a	
Largest diff. peak and hole	0.575 and -0.538 e.Å^{-3}	

Antibacterial activity and cytotoxicity

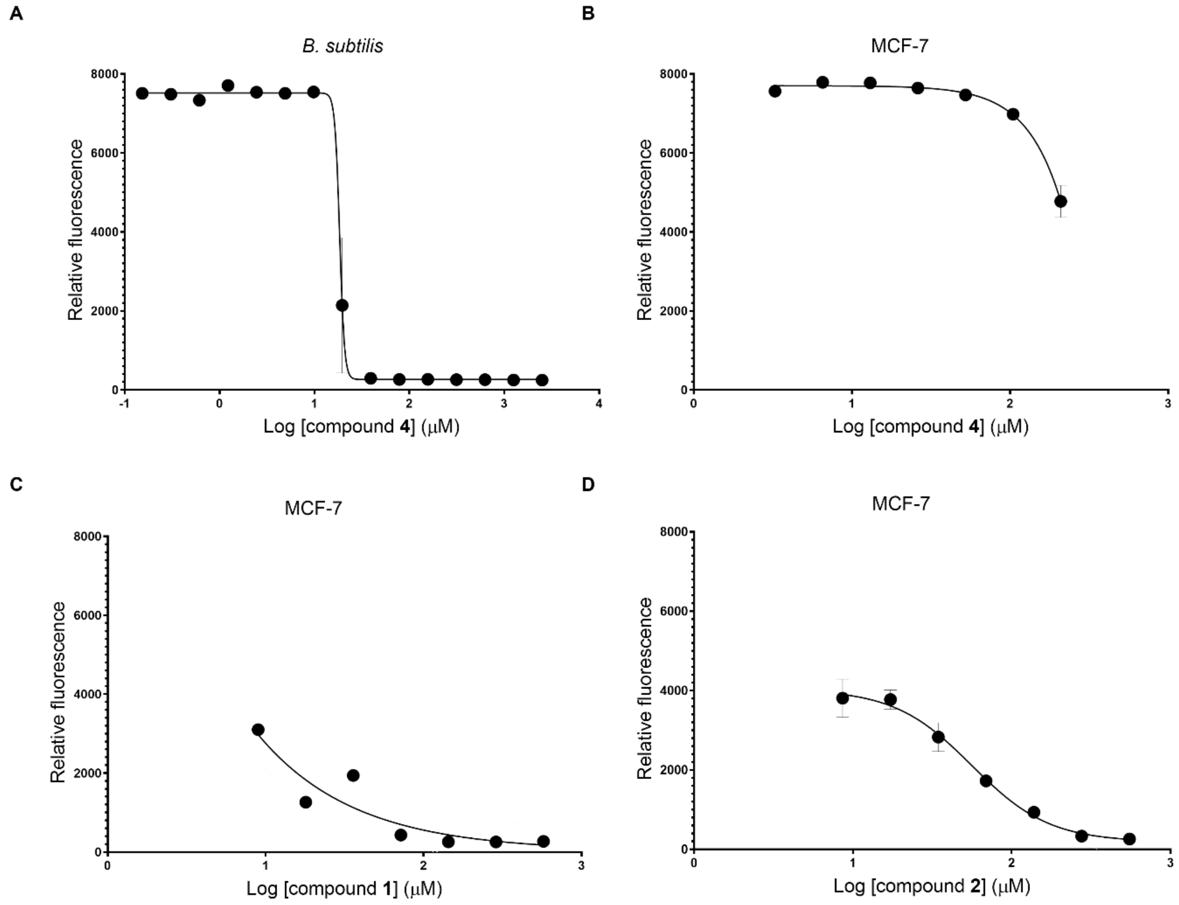

Figure S85. Antibacterial activity and cytotoxicity dose-response curves. Antibacterial activity (**A**) dose-response curves of compound **4** against Gram-positive *Bacillus subtilis* showed EC_{50} = 18 µM (SE range of 16 to 20), EC_{90} = 22 µM (SE range of 18 to 26) and MIC = 9 µM. Cytotoxicity of compounds **4** (**B**), **1** (**C**) and **2** (**D**) against MCF-7 cells presented EC_{50} of 247 µM (SE range of 90 to 380), 65 µM (SE range of 50 to 83) and 92 µM (SE range of 86 to 100), accordingly (see the following **Table S3**).

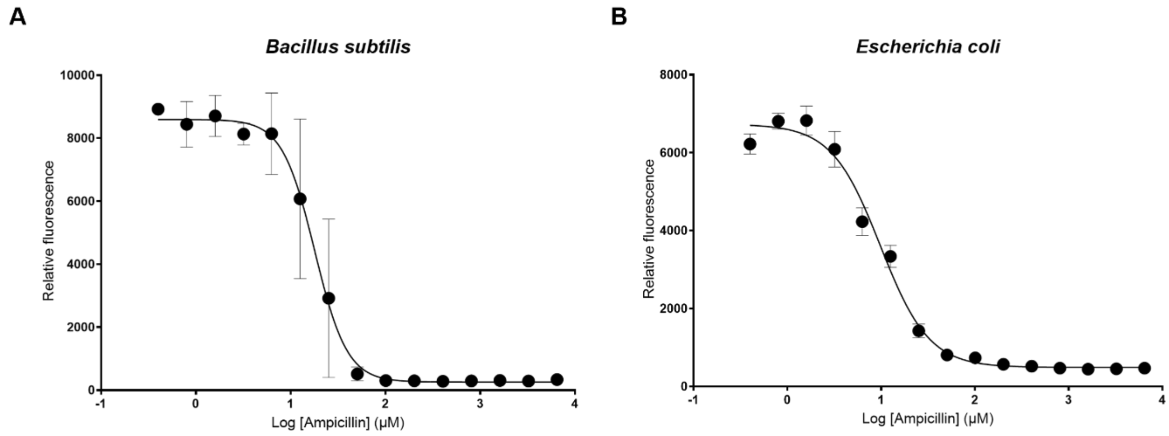

Figure S86. Measurements of the antibacterial activity of Ampicillin, as a positive control antibiotic tested against (**A**) Gram-positive *Bacillus subtilis* and Gram-negative *Escherichia coli* (**B**) following the same assays conditions as for the isolated natural compounds (see experimental section). As previously reported by European Committee on Antimicrobial Susceptibility Testing (Data from the EUCAST MIC distribution website, last accessed November 2020". http://www.eucast.org), Ampicillin showed an EC_{50} = 17.2 µM (SD = 8.910; SE range of 13.490 to 23.778 µM) and an EC_{90} = 160.4 µM (SD = 42.786; SE range of 97.127 to 146.531 µM) against *B. subtilis* and an EC_{50} = 11.5 µM (SD = 0.721; SE range of 11.065 to 11.898 µM) and an EC_{90} = 89.2 µM (SD = 14.807; SE range of 81.493 to 98.591 µM) against *E. coli*.

Table S3. Antibacterial and cytotoxic activities of isolated compounds from *S. usambarensis*.

	E. coli					***B. subtilis***							**Cytotoxicity MCF-7**		
**S/N**	**EC50** (µM)	**SE range** (µM)		**EC90** (µM)	**MIC** (µM)	**EC50** (µM)	**SE range** (µM)		**EC90** (µM)	**SE range** (µM)		**MIC** (µM)	**EC50** (µM)	**SE range** (µM)	
**1**	>6600	-	-	-	>6600	-	-	-	-	-	-	-	65	50	83
**2**	>3400	-	-	-	>3400	-	-	-	-	-	-	-	92	86	100
**4**	-	-	-	-	-	18	16	20	22	18	26	9.0	247	90	380
**5**	>2500			>2500	>2500	189	187	190	230	220	240	106.3	570	560	580
**9**	-	-	-	-	-	400	325	475	540	420	660	>798	>740	-	-
**10**	-	-	-	-	-	6200	6100	6300	8200	8100	8300	>2400	>700	-	-
**12**	5100	5000	5200	-	>5700	-	-	-	-	-	-	-	>1100	-	-
Ampicillin	13.7	19.927	10.568	20.8	-	7.5	6.382	8.933	62.2	49.065	73.343	-	-	-	-
